# (E)-7-Ethylidene-lithocholic Acid (7-ELCA) Is a Potent Dual Farnesoid X Receptor (FXR) Antagonist and GPBAR1 Agonist Inhibiting FXR-Induced Gene Expression in Hepatocytes and Stimulating Glucagon-like Peptide-1 Secretion From Enteroendocrine Cells

**DOI:** 10.3389/fphar.2021.713149

**Published:** 2021-08-13

**Authors:** Alzbeta Stefela, Miroslav Kaspar, Martin Drastik, Thales Kronenberger, Stanislav Micuda, Martin Dracinsky, Blanka Klepetarova, Eva Kudova, Petr Pavek

**Affiliations:** ^1^ Department of Pharmacology and Toxicology, Faculty of Pharmacy in Hradec Kralove, Charles University, Hradec Kralove, Czechia; ^2^ Institute of Organic Chemistry and Biochemistry of the Czech Academy of Sciences, Prague, Czechia; ^3^ Faculty of Sciences, Charles University, Prague, Czechia; ^4^ Department of Physical Chemistry and Biophysics, Faculty of Pharmacy in Hradec Kralove, Charles University, Hradec Kralove, Czechia; ^5^ Department of Internal Medicine VIII, University Hospital of Tübingen, Tübingen, Germany; ^6^ School of Pharmacy, University of Eastern Finland, Faculty of Health Sciences, Kuopio, Finland; ^7^ Department of Pharmacology, Faculty of Medicine in Hradec Kralove, Charles University, Hradec Kralove, Czechia

**Keywords:** G protein-coupled bile acid receptor 1, bile acids, steroid, farnesoid X receptor, metabolism

## Abstract

Bile acids (BAs) are key signaling steroidal molecules that regulate glucose, lipid, and energy homeostasis via interactions with the farnesoid X receptor (FXR) and G-protein bile acid receptor 1 (GPBAR1). Extensive medicinal chemistry modifications of the BA scaffold led to the discovery of potent selective or dual FXR and GPBAR1 agonists. Herein, we discovered 7-ethylidene-lithocholic acid (7-ELCA) as a novel combined FXR antagonist/GPBAR1 agonist (IC_50_ = 15 μM/EC_50_ = 26 nM) with no off-target activation in a library of 7-alkyl substituted derivatives of BAs. 7-ELCA significantly suppressed the effect of the FXR agonist obeticholic acid in BSEP and SHP regulation in human hepatocytes. Importantly, 7-ELCA significantly stimulated the production of glucagon-like peptide-1 (GLP-1), an incretin with insulinotropic effect in postprandial glucose utilization, in intestinal enteroendocrine cells. We can suggest that 7-ELCA may be a prospective approach to the treatment of type II diabetes as the dual modulation of GPBAR1 and FXR has been supposed to be effective in the synergistic regulation of glucose homeostasis in the intestine.

## Introduction

Bile acids (BAs) are amphipathic steroidal molecules that facilitate the absorption of lipids, but they are also important signaling agents acting as ligands of the nuclear farnesoid X receptor (FXR) and the membrane G-protein coupled bile acid receptor 1 (GPBAR1, also known as Takeda G protein-coupled receptor 5, TGR5) (Donkers et al., 2019; Keitel et al., 2019; Kecman et al., 2020). Chenodeoxycholic acid (3α,7α-dihydroxy-5β-cholanic acid, CDCA) is the most potent endogenous FXR ligand (Ahmad and Haeusler, 2019), whereas lithocholic acid (3α-hydroxy-5β-cholan-24-oic acid, LCA) and its taurine conjugate, taurolithocholic acid (TLCA), activate the GPBAR1 with the highest potency among natural BAs.

The FXR functions as an enterohepatic regulator of bile acid homeostasis, cholesterol, lipid, glucose, and amino acid metabolism and inflammation (Han, 2018; Massafra et al., 2018). The intestinal GPBAR1 is important in the regulation of glucose metabolism and insulin resistance (Keitel et al., 2019). In addition, GPBAR1 positively regulates energy expenditure in adipocytes and muscle cells (Watanabe et al., 2006; Arab et al., 2017; Keitel et al., 2019).

FXR and GPBAR1 exert distinct but also overlapping effects in the intestine and the liver (Downes et al., 2003; Han, 2018; De Marino et al., 2019; Di Leva et al., 2019; Donkers et al., 2019). Recent research provides compelling evidence suggesting that antagonism of intestinal FXR signaling improves glucose metabolism, alleviates insulin resistance, and may improve nonalcoholic fatty liver disease (Gonzalez et al., 2017; Han, 2018; Sun et al., 2018; van Zutphen et al., 2019). Similarly, the activation of GPBAR1 increases glucagon-like peptide-1 (GLP-1) secretion from enteroendocrine L cells, which stimulates insulin secretion from pancreatic β-cells (Katsuma et al., 2005). Therefore, the development of combined FXR antagonists/GPBAR1 agonists might provide a synergistic therapeutic strategy in the regulation of glucose homeostasis mediated by intestinal endocrine cells (Downes et al., 2003; Han, 2018; De Marino et al., 2019; Di Leva et al., 2019; Donkers et al., 2019).

Previous reports suggest that modification of the steroidal scaffold allows development of both FXR antagonists and GPBAR1 agonists. A natural steroid Z-guggulsterone (Z-GUG) isolated from *Commiphora mukul* is referred as the first described FXR antagonist. However, Z-GUG is today considered as a selective bile acid receptor modulator (Urizar et al., 2002; Cui et al., 2003; Sepe et al., 2015). Natural tauro-conjugated α- and β-muricholic acids (α/β-MCA) have also been described as FXR antagonists with IC_50_ values of 28 and 40 μM, respectively, in a co-activator assay (Li et al., 2013; Sayin et al., 2013). Glycine-β-muricholic acid (Gly-MCA) was synthesized as a more stable FXR antagonist based on *in silico* modeling of tauro-β-MCA (Gonzalez et al., 2016). In contrast, the glycol- and tauro-ursodeoxycholic acid (GUDCA, TUDCA) are supposed to be natural human weak FXR antagonists, with IC_50_ = 77.2 and 75.1 µM, respectively (Sun et al., 2018). Besides the previously mentioned BAs and their derivates, different polyhydroxylated or sulfated sterols from plants or marine organisms exhibit weak to moderate FXR antagonistic activity as well (Sepe et al., 2015; Sepe et al., 2018; De Marino et al., 2019). Nevertheless, no potent BA derived antagonist with the capacity to reverse an agonist-mediated activation of FXR has been described, so far.

On the other hand, after the discovery of obeticholic acid (6α-ethyl-chenodeoxycholic acid, OCA, INT-747), the first-in-class FXR ligand (Pellicciari et al., 2002) approved for the treatment of resistant primary biliary cholangitis (PBC), the structural modifications of BAs have been intensively focused on the development of selective or dual FXR and GPBAR1 agonists (Fiorucci et al., 2019; Ratziu et al., 2019). The removal of the hydroxyl group at C-3 on CDCA or OCA scaffolds generated 3-deoxy-BA derivatives that still transactivated FXR, but were devoid of any activity toward GPBAR1 (Sepe et al., 2015; Sepe et al., 2016a; Carino et al., 2018). Another structure-activity relationship study led to the discovery of 5β-cholan-24-oic acid and 5α-cholan-24-oic acid as the first examples of BA derivatives endowed with FXR agonism and GPBAR1 antagonism (Sepe et al., 2016b). Other modifications led to the synthesis of potent dual FXR/GPBAR1 agonists such as the compound INT-767 (Rizzo et al., 2010) or the steroidal alcohol BAR502 (Festa et al., 2014). A marked selectivity toward GPBAR1 over FXR has been achieved with the methylation of the BA scaffold at C-7 or C-23 position (Pellicciari et al., 2009; Iguchi et al., 2011; Nakhi et al., 2019), with the introduction of a hydroxyl group in β-configuration at C-16 of OCA (Pellicciari et al., 2012), or with the introduction of 7β-hydroxyl groups (Festa et al., 2014; Sepe et al., 2014). In addition, a strong capacity to activate GPBAR1 were recently described for an endogenous BA, cholic acid 7-sulfate (Chaudhari et al., 2021). Nevertheless, no steroidal GPBAR1 agonist with combined FXR antagonizing capacity has been introduced, so far.

**FIGURE 1:**
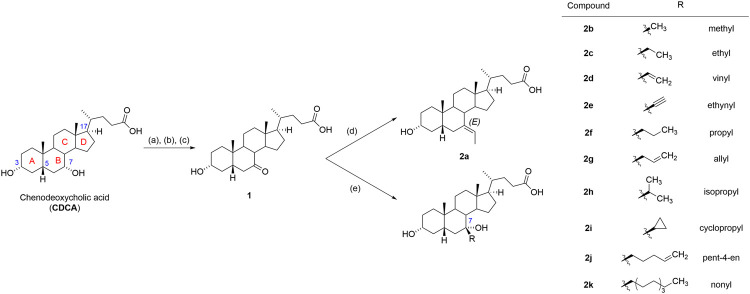
Reagents and conditions. **(A)** H_2_SO_4_, MeOH, reflux; **(B)** CrO_3_, H_2_SO_4_, H_2_O, acetone, 0°C; **(C)** NaOH, MeOH, H_2_O, 50°C; **(D)** NaH, ethyltriphenylphosphonium bromide, THF, reflux, overnight; **(E)** RMgCl for 2f, 2h or RMgBr for 2b, 2c, 2d, 2e, 2g, 2i, 2j, 2k, THF, reflux, 2 h. For CDCA, rings are lettered and steroid ring numbering at biologically significant positions and is included.

In the study, we report on the 7β-alkyl substituted BA derivatives endowed with unique and potent dual FXR antagonistic and GPBAR1 agonistic activities. The most efficacious compounds 7-ethylidene-LCA (7-ELCA) and 7β-isopropyl-CDCA (2h) (Figure 1) significantly suppressed activities of the potent FXR agonist OCA in the regulation of FXR target genes. In addition, 7-ELCA activates GPBAR1 at nanomolar concentrations with 50 times lower EC_50_ than LCA, suggesting it is one of the most potent steroidal GPBAR1 agonists described up to date. Detailed pharmacological evaluations have shown that 7-ELCA significantly stimulates the secretion of GLP-1 in human intestinal endocrine cells and suppresses FXR target gene expression in hepatocytes exposed to the FXR ligand obeticholic acid.

## Materials and Methods

### Chemicals

Rifampicin, dexamethasone, forskolin (FSK), Diprotin A, 3-isobutyl-1-methylxanthine (IBMX), chenodeoxycholic acid (CDCA, Cat. No. 700198P), 1α,25-dihydroxyvitamin D_3_ (D1530), fenofibrate, rosiglitazone, GW501516 (SML1491), GW3964 (G6295), thyroxin (T1775), GW4064 (Cat. No. G5172), (Z)-guggulsterone (Z-GUG; Cat. No. 78251) were purchased form Sigma-Aldrich, now Merck, Darmstadt, Germany). Obeticholic acid (Synonyms: OCA; INT-747; 6-ECDCA; 6-ethylchenodeoxycholic acid; Cat. No HY-12222) was purchased form MCE MedChem Express (NJ, United States). CITCO were obtained from Tocris (3683/10, Minneapolis, MN, United States). Tauro-beta-muricholic acid (Tβ-MCA) has been obtained from Cayman (Cay20289-5; Ann Arbor, MI, United States).

### Cell Culture

Human hepatocellular carcinoma HepG2 cells (purchased from the European Collection of Cell Cultures, ECACC, Salisbury, United Kingdom) were cultured in antibiotic-free Dulbecco's modified Eagle's medium (DMEM, Sigma-Aldrich, now Merck, Darmstadt, Germany), supplemented with 10% fetal bovine serum (FBS), 1% L-glutamine and 1% sodium pyruvate. The HepaRG™ cells (Biopredic, Rennes, France) were seeded at the density of 26,600 cells/cm^2^ and kept in William’s medium, supplemented with 5 µg/ml insulin, 50 µM hydrocortisone, 10% Hyclone fetal serum (GE Healthcare Life Sciences) and 1% L-glutamine. 14 days after seeding, the HepaRG cells were differentiated to hepatocyte-like cells using 1.5% DMSO in culture media for another 14 days. Human enteroendocrine colon cancer NCI-H716 cells (ATCC-CCL-251, ECACC) were cultured in antibiotic-free RPMI-1640 medium, supplemented with 10% FBS. For each experiment, the NCI-H716 cells were seeded onto 96-well Matrigel (Corning®) coated plates (1 × 10^5^ cells/well) and differentiated for 48 h. GLUTag cells were kindly provided by Dr. Colette Roche (Centre de Recherche en Cancérologie de Lyon, INSERM U1052, Lyon, France) with the permission of Dr. Daniel J. Drucker (Lunenfeld Tanenbaum Research Institute Mt. Sinai Hospital, Toronto). GLUTag cells were cultured in DMEM supplemented with 10% FBS. Primary human hepatocytes were purchased from Biopredic (Rennes, France; Lot N. HEP2201023, Human Long-term hepatocytes in monolayer, Caucasian male, 71 years old).

### Transient Transfection and Luciferase Gene Report Assays

For transient transfection, HepG2 cells were seeded at the density of 40,000 cells/cm^2^. In the case of the determination of GPBAR1 activation, cells were transfected using Lipofectamine 2000® (ThermoFisher Scientific, Waltham, MA, United States) with 200 ng CRE luciferase reporter vector (pGL4.29[luc2P/CRE/Hygro], Promega, Hercules, CA, United States), together with 150 ng GPBAR1 (GPBAR1-pcDNA3.1+/C-(K)-DYK) (Genscript, Piscataway, NJ, United States) or empty vector pcDNA3.1 and 50 ng pRL-TK *Renilla* luciferase vector (Promega). The next day, cells were challenged with tested ligands in indicated concentrations for 5 h. Forskolin (FSK) was used as a positive control for the generation of cAMP. All the other transient transfection reporter gene assays were performed with Lipofectamine® 3000 (ThermoFisher Scientific). Briefly, cells were transfected with 150 ng/well luciferase reporter gene constructs (p(DR3)_3_-luc, SHP-luc, p3A4-luc, pTAT-(GRE)2-TK-luc, p2B6-luc, pGL4.35[luc2P⁄9XGAL4UAS⁄Hygro]), FXRE-luc together with 100 ng/well of the corresponding full-length fragment nuclear receptor expression vectors (pSG5-hVDR, pSG5-hFXR, pSG5-hRXRα, pSG5-hPXR, CAR3 variant) or the ligand binding domains of human, pCMX-GAL4-hFXR, pCMX-GA4L-LXRα, pCMX-GA4L-LXRα, pCMX-GA4L-TRα, pCMX-GA4L-PPARα, pCMX-GAL4-PPARγ, pCMX-GAL4-PPARδ) and 30 ng/well of pRL-TK *Renilla* luciferase vector 24 h prior to the treatment. Construct are described in our previous papers (Dvorak et al., 2008; Carazo et al., 2018; Stefela et al., 2020). The firefly luciferase activity was measured using the Dual Luciferase® Reporter Assay System (Promega) and normalized to *Renilla* luciferase activity. Experiments in an agonistic mode were performed with OCA, CDCA or tested compounds (all compounds at 10 μM). Experiments in an antagonistic mode were performed by co-treating cells with 1 µM OCA and tested compounds at 10 and 40 µM concentration or at increasing concentrations (ranging from 0.001 to 200 µM) in the case of IC_50_ determination. The EC_50_ and IC_50_ are theoretical concentrations of a tested compound that provide half-maximal activation and inhibition, respectively, in reporter gene assays. EC_50_ and IC_50_ were calculated using GraphPad PRISM ver. 9.1.0. software (San Diego, CA, United States) employing a non-linear regression module. All the experiments have been repeated at least three times and each experiment was performed in biological triplicates (*n* = 3). Results are presented as fold change to control nontreated (NT) samples. Vehicle (0.1% DMSO) was used as a solvent in all samples including control samples.

### Preparation of G-Protein Bile Acid Receptor 1 Mutants

The substitutions of amino acids Ser270, Tyr89 and Glu169 for glycines were inserted in the GPBAR1 gene cloned into the pcDNA3.1 and shuttle expression vector using a Quick Change XL Site-Directed Mutagenesis Kit (Agilent, Santa Clara, CA, United States). The mutagenic primers were created using Quick Change Primer Design software from Agilent. (Ser270: 5′-CCT​CTC​CCT​AGG​AGG​CGC​CAG​TGC​AGC-3′ (F) 5′- GCT​GCA​CTG​GCA​CCG​CCT​AGG​GAG​AGG - 3′; Tyr89: 5′- GTC​CTG​CCT​CCT​CGT​CGG​CTT​GGC​TCC​CAA​CTT​C – 3′ (F) 5′- GAA​GTT​GGG​AGC​CAA​GCC​GAC​GAG​GAG​GCA​GGA​C – 3′ (R); Glu169: 5′- CAG​GAG​CCC​ATA​GAC​GCC​GAG​GTA​CAG​GTA​GGG – 3′ (F), 5′- CCC​TAC​CTG​TAC​CTC​GGC​GTC​TAT​GGG​CTC​CTG – 3′ (R). The plasmid constructs were transformed into *E. coli* XL10-Gold ultracompetent cells according to the manufacturer’s guideline. The final gene mutations were confirmed by sequencing.

### siRNA Transfection

GLUTag cells were transfected with non-targeting scrambled siRNA (siRNA control) or with the combination of siRNAs specific for Gpbar1 (Silencer® Select Pre-designed siRNA, LOT# ASO2HKN6 and ASO2HKN7, Life Technologies, Carlsbad, CA, United States) twice at 24 and 72 h after seeding using Lipofectamine RNAiMAX reagent (Life Technologies, Carlsbad, CA, United States). GLUTag cells were then treated with tested molecules at the day 4 post-seeding.

### RNA Isolation and Real-Time qPCR

Total RNA from HepaRG cells or primary human hepatocyte samples was isolated by the phenol/chloroform method with TRI-reagent (Merck) according to the manufacturer's protocol. The cDNA was synthesized using a Tetro cDNA Synthesis Kit (Bioline, now Meridian Bioscience, Memphis, United States), and RT-qPCR was run in the Quant Studio 6 instrument using the Fast Advanced Master Mix (ThermoFisher Scientific, Waltham, United States) according to the MIQE protocols. All the probes were obtained from ThermoFisher Scientific: ABCB11 (BSEP, Hs00184824_m1), NR0B2 (SHP, Hs00222677_m1). Data were normalized to beta-2 microglobulin (B2M (Mm00437762_m1) as the reference gene and were evaluated by the ΔΔCq method. All the experiments have been repeated three times and each experiment was performed in biological triplicates (*n* = 3). Results are presented as fold change of mRNA expression to control nontreated (NT) samples.

### Protein Determination

Protein determination was performed from whole-cell lysates of terminally differentiated HepaRG cells treated for 48 h. Protein levels were quantified using a PierceTM BCA Protein Assay Kit (ThermoFisher Scientific, Waltham, United States) according to the manufacturer's protocol. The western blotting analysis was performed as described in our previous paper (Stefela et al., 2020) with mouse monoclonal anti-SHP antibody (clone OTI5F10, Cat. No. TA806319, Origene, Rockville, MD, United States) and antibody against GAPDH (Cell Signaling Technology, Leiden, the Netherlands) as a loading control. Protein concentration was measured using the BCA protein assay (Sigma-Aldrich/Merck, Prague, Czech Republic). Protein expression quantification was done using densitometric software (LabImage, Kapelan Bio-Imaging, Germany).

### LanthaScreen® Time-Resolved Fluorescence Energy Transfer Farnesoid X Receptor Coactivator Assay

The LanthaScreen® TR-FRET FXR Coactivator Assay (goat, PV4833, ThermoFisher Scientific) was performed to assess the affinity of tested compounds to FXR ligand-binding domain (LBD) in agonistic and antagonistic models. In the antagonistic assays, the FXR LBD was co-incubated with OCA or GW4064, together with tested ligands in increasing concentrations. The assay was performed according to the manufacturer’s protocols. After an 1 h incubation period at room temperature, the TR-FRET ratio of 520/495 nm was measured using the Biotek plate reader and used to calculate the IC_50_ values from the concentration-response curves of each compound using GraphPad Prism version 9.1.0. software. Data have been obtained from three independent experiments performed in 4 replicates.

### Glucagon-like Peptide-1 Secretion Analysis

After serum starvation, differentiated NCI-H716 and NCI-H716 cells were washed in PBS and incubated with the tested compound in Hanks' Balanced Salt Solution (HBSS) supplemented with 0.2% (w/v) BSA and 50 µM Diprotin A (Sigma-Aldrich/Merck) for 1 or 2 h, respectively. Supernatants were collected and centrifuged. The quantity of GLP-1 was determined using the Glucagon Like Peptide-1 (Active) ELISA kit (EZGLP1T-36K, Merck Millipore, Burlington, MA, United States) according to the manufacturer's instructions. Data were normalized to protein concentration, and they are presented as a fold protein increase to control nontreated (NT) samples. Vehicle (0.1% DMSO) was present in the control as well as in other samples. All the experiments have been repeated three times and each experiment was performed in biological triplicates (*n* = 3).

### Determination of cAMP

Differentiated NCI-H716 cells were serum-starved, washed in PBS, and the cell culture medium was changed to Hanks' Balanced Salt solution (HBSS) supplemented with 0.2% (w/v) BSA and 500 µM 3-isobutyl-1-methylxanthine and incubated for 45 min at 37°C. Cells were stimulated with tested compounds or forskolin for 30 min, and the amount of cAMP generated was measured using the cAMP-Glo™ Assay (V1501, Promega, Hercules, CA, United States). The changes in cAMP levels (ΔcAMP) are presented as cAMP levels in treated samples after subtraction of the cAMP levels in the nontreated (NT) samples. All the experiments have been repeated three times and each experiment was performed in biological triplicates (*n* = 3).

### Statistical Analysis

Statistical analyses were performed using GraphPad Prism 9.1.0. software (GraphPad Software, Inc., San Diego, CA, United States), with a *p*-value of <0.05 considered statistically significant. All data are presented as the mean ± standard deviations (SDs) based on at least three independent experiments (*n* = 3). A one-way analysis of variance (ANOVA) with a Dunnett’s or Bonferroni’s post-hoc test was applied to the data if more than two groups were being analyzed. The half maximal inhibitory concentration (IC_50_) and the half-maximal response (EC_50_) values were calculated using nonlinear fitting of concentration-response curves (log(inhibitor) vs. normalized response) or (log(agonist) vs. response (three parameters)), respectively.

### Farnesoid X Receptor Ligand Binding Domain Docking and Molecular Dynamics Simulations

For the procedure of FXR LBD docking experiments and molecular dynamics, see Supplementary Material *.*

### G-Protein Bile Acid Receptor 1 Docking

The 3D structures of ligand molecules were designed in PerkinElmer Chem3D (version 19.0.1.28). The energy minimization was done utilizing an inbuilt MM2 force field. Ligands were then exported to PDB files. All ligands were prepared for docking by the AutoDockTools 1.5.6 (Morris et al., 2009) python script “prepare_ligand4.py”. This procedure consists of assigning Gasteiger charges, merging non-polar hydrogens, building the torsion tree, and then exporting the data to PDBQT.

The preparation of the receptor (PDB 7CFN) was performed in the AutoDockTools using a standard protocol. In particular, all chains but R together with water molecules were deleted, non-polar hydrogens were merged, and Kollman charges were calculated. The grid box, which securely covered the whole LBD and the pocket entrance on the extracellular part of the receptor, was defined as a cube with a side length of 30 Grid points (1 Å spacing) and with its center at X: 98, Y: 124, Z: 119, roughly at the level of the INT-777 D-ring.

Molecular docking was performed with AutoDock Vina 1.1.2 (Trott and Olson, 2010). The exhaustiveness was set to 16, the rest of the parameters were kept at default values. Five independent runs were conducted, and the average affinity for the corresponding poses was taken as the final affinity value. Visualization of docking results was generated in Chimera 1.14 (Pettersen et al., 2004). Only residues within a 5 Å distance from the INT-777 pose are displayed. All other residues and all non-polar hydrogens are omitted for clarity. LigPlot+ v.2.2 (Laskowski and Swindells, 2011) was employed to generate the 2D ligand-protein interaction diagram.

### Synthesis of 7β-Alkyl Substituted Bile Acids

All commercial reagents and solvents were used without purification. Melting points were determined with a Hund/Wetzlar micromelting point apparatus (Germany), and are uncorrected. ROESY NMR spectra were obtained using a Bruker Avance III™ HD 500 MHz and/or a JEOL ECZ500 spectrometer, both operating at 125.7 MHz for ^13^C and 500 MHz for ^1^H. The assignment of hydrogen and carbon signals was based on a combination of 1D and 2D NMR experiments (^3^H, ^13^C-APT, ^1^H,^1^H COSY, ^1^H,^13^C HSQC and ^1^H,^13^C HMBC). Proton and carbon NMR spectra were measured in a Bruker AVANCE III™ 400 or 500 MHz with chemical shifts given in parts per million (ppm) (δ relative to residual solvent peak for ^1^H and ^13^C). Coupling constants (*J*) are given in Hz. The HR-MS spectra were performed with LCQ Advantage (ThermoFisher Scientific, Waltham, MA, United States) using ESI mode. Thin-layer chromatography (TLC) was performed on silica gel (Merck, 60 µm). For column chromatography, neutral silica gel 60 µm (Fluka, Buchs, Switzerland) was used. Analytical samples were dried over phosphorus pentoxide at 50°C/0.25 kPa. The purity of final compounds was assessed by HPLC analysis with ELS detection (evaporative light scattering), and all corresponding chromatographs are enclosed in Supplementary Material.

### Analytical HPLC Method A

Analysis was carried out on a HPLC Gilson system (United States) equipped with ELS detector. Solvent A was DCM/AcOH (1000:1), and solvent B was MeOH/AcOH (1000:1). Analysis was performed in isocratic setup as 95/5 A/B with flow rate 1 ml/min, column: Supelco, bare-silica LC-SI 5 μm, 150 × 4.6 mm. The sample was prepared by dissolving the sample (1 mg) in DCM (1 ml) and by then being sonicated for 5 min 20 µl was injected into the LC system.

### Analytical LCMS Method B

Analysis was carried out on LC-MS system LCQ Advantage (Thermo Fisher Scientific). Ions were detected in negative ESI ion mode, with m/z range from 250 to 1500 Da. Solvent A was water/acetonitrile (98:2), and solvent B was acetonitrile/isopropanol/water (95:3:2), with 5 mM ammonium formate in both. Gradient setup: 0-25-30-30.1-45 min, 50-100-100-50-50% of solvent B and flow rate 150 µL/min, column: Phenomenex, C4, Jupiter® 5 μm, 250 × 4.6 mm. The sample was prepared by dissolving the sample (1 mg) in solution A/B (1:3, 1 ml) and by then being sonicated for 5 min. 10 µl was injected into the LCMS system.

### Experimental Data for Compounds 2a-2k

General Procedure for Grignard Reaction. A solution of 3α-hydroxy-7-oxo-5β-cholan-24-oic acid (1, 1.28 mmol, 500 mg) was added dropwise at room temperature to a solution of Grignard reagent (6.4 mmol) in dry THF (10 ml) under an inert atmosphere. Upon addition, a cloud-like precipitate formed. The solution was then vigorously stirred and heated to reflux. The progress of the reaction was monitored by TLC. After 2 h, the reaction mixture was acidified with aqueous 1M HCl to pH 2 and extracted with EtOAc (3 × 75 ml). The combined extracts were washed with water, brine, dried over Na_2_SO_4_, and the solvents were evaporated. The crude product was purified by column chromatography on silica gel (MeOH/DCM, 2:98 to 5:95 v/v), followed by purification on semi-preparative HPLC (Column, Luna® 5 µm bare-silica 250 × 21.2 mm, Isocratic MeOH/DCM, 3:97 v/v, 15 ml/min, injected in either DCM or THF - if insoluble in DCM).

3α-Hydroxy-7-oxo-5β-cholan-24-oic acid (1). 7-Oxolithocholic acid methyl ester (Stefela et al., 2020) (8.2 g, 20.27 mmol) was dissolved in 300 ml of 5% NaOH in MeOH/H_2_O (1:1) and heated to 50°C. After 2 h, aqueous solution of HCl (5%) was added to pH 3. The product was extracted with EtOAc (3 × 200 ml), combined organic extracts were washed with brine (1 × 300 ml) and dried over anhydrous Na_2_SO_4_. After solvent evaporation, the oily residue (8.2 g) was purified by flash chromatography (EtOAc/hexane/AcOH, 30/70/1, v/v/v), and further crystallized from boiling EtOAc to afford compound 1 (7.6 g, 96% yield). R_f_ (TLC) = 0.43 (acetone/hexane/AcOH, 40/60/1), mp 202–203 °C (EtOAc), lit. (Fieser and Rajagopalan, 1950), 202–203°C. [α]_D_^25^ −29.6 (*c* 0.28, MeOH). ^1^H NMR (401 MHz, MeOD): δ 3.53 (tt, *J*_*1*_ = 10.5 Hz, *J*_*2*_ = 4.7 Hz, ^1^H, H-3), 2.99 (ddd, *J*_*1*_ = 12.5, *J*_*2*_ = 6.0 Hz, *J*_*3*_ = 1.1 Hz, 1H, H-6a), 2.54 (t, *J* = 11.3 Hz, _1_H, H-8), 1.23 (s, 3H, H-19), 0.96 (d, *J* = 6.5 Hz, 3H, H-21), 0.71 (s, 3H, H-18). ^13^C NMR (101 MHz, MeOD): δ 215.1 (C-7), 178.1 (C-24), 71.5 (C-3), 56.3, 50.7, 50.4, 47.5, 46.4, 44.4, 43.8, 40.3, 38.2, 36.6, 36.3, 35.2, 32.3, 32.0, 30.6, 29.3, 25.8, 23.5, 22.8, 18.8, 12.5. MS (ESI neg): *m/z* 389.3 (100%, M−H), 435.3 (5%, M+FA−H), 779.5 (3%, 2M−H). HR-MS (ESI neg): *m/z* calcd for C_24_H_37_O_3_ [M−H], 389.26938; found, 389.26973. For C_24_H_38_O_4_ calcd C 73.81, H 9.81. Found: C 73.72, H 9.57.

(E)-3α-Hydroxy-7-ethylidene-5β-cholan-24-oic acid (7-ELCA, 2a). Sodium hydride (60% in mineral oil, 59 mg, 1.48 mmol) was added to a solution of ethyltriphenylphosphonium bromide (550 mg, 0.51 mmol) in dry THF (15 ml) under an inert atmosphere. The reaction mixture was refluxed until a deep orange color formed. Then, the solution was cooled to 50°C, and a solution of 7-keto-LCA (1, 200 mg, 1.48 mmol) in dry THF (10 ml) was slowly added dropwise. After overnight reflux, the reaction mixture was poured into a beaker with crushed ice and extracted with EtOAc (3 × 50 ml). The combined extracts were washed with water, brine, dried over Na_2_SO_4_, and solvents evaporated. The crude product was purified by column chromatography on silica gel (MeOH/DCM, 2:98 to 5:95 v/v), followed by purification on semi-preparative HPLC (Column, Luna® 5 µm bare-silica 250 × 21.2 mm, Isocratic MeOH/DCM, 3:97 v/v, 15 ml/min, injected in DCM) affording compound 2a as a slightly yellowish powder (6 mg, 3%): R_f_ (TLC) = 0.69 (EtOAc/hexane/AcOH, 50/50/1), mp 67–72°C. ^1^H NMR (500 MHz, MeOD): δ 5.30 (q, *J* = 6.7 Hz, _1_H, H-1′), 3.58–3.50 (m, 1H, H-3), 1.59–1.56 (m, 3H, H-2′), 1.08 (s, 3H, H-19), 0.96 (d, *J* = 6.5 Hz, 3H, H-21), 0.71 (s, 3H, H-18). ^13^C NMR (126 MHz, MeOD): δ 176.5 (C-24), 140.9 (C-6), 115.6 (C-1′), 72.0 (C-3), 56.4, 51.4, 46.5, 44.3, 44.12, 44.09, 40.5, 37.1, 37.1, 36.6, 36.1, 32.6, 32.3, 31.8, 31.0, 29.1, 26.5, 24.3, 22.1, 18.9, 13.3, 12.7. MS (ESI neg): *m/z* 401.3 (76%, M−H), 447.3 (100%, M+FA−H), 803.6 (10%, 2M−H). HR-MS (ESI neg): *m/z* calcd for C_26_H_41_O_3_ [M-H], 401.3061; found, 401.3062. LCMS method B (ESI neg, t_R_ = 17.45 min). Purity 97.5% (HPLC method A, t_R_ = 5.26 min).

3α,7α-Dihydroxy-7β-methyl-5β-cholan-24-oic acid (7β-methyl-CDCA, 2b). Compound 2b was prepared according to General Procedure for Grignard Reaction. Starting from compound 7-keto-LCA (1, 500 mg, 1.28 mmol), compound 2b (153 mg, 29%) was obtained as white solids: R_f_ (TLC) = 0.28 (DCM/MeOH, 5/95), mp 85–88°C, lit. (Une et al., 1989) 96–99°C, [α]_D_^25^ + 29.9 (c 0.15, MeOH). ^1^H NMR (401 MHz, CDCl_3_): δ 3.49 (tt, *J* = 11.0, 4.5 Hz, 1H, H-3), 1.22 (s, 3H, H-1′), 0.95 (d, *J* = 6.4 Hz, 3H, H-21), 0.87 (s, 3H, H-19), 0.68 (s, 3H, H-18). ^13^C NMR (101 MHz, CDCl_3_): δ 179.0 (C-24), 73.2 (C-7), 72.1 (C-3), 54.9, 51.5, 44.4, 44.2, 43.3, 42.1, 40.2, 38.5, 36.2, 35.7, 35.5, 34.7, 33.7, 31.0, 30.9, 30.5, 28.6, 28.2, 23.0, 21.4, 18.6, 12.4. MS (ESI neg): *m/z* 405.3 (100%, M−H), 451.3 (11%, 2M−H). HR-MS (ESI neg): *m/z* calcd for C_25_H_41_O_4_ [M−H], 405.30103; found, 405.30043. LCMS method B (ESI neg., t_R_ = 12.66 min). Purity 95.6% (HPLC method A, t_R_ = 6.53 min).

3α,7α-Dihydroxy-7β-ethyl-5β-cholan-24-oic acid (7β-ethyl-CDCA, 2c). Compound 2c was prepared according to General Procedure for Grignard Reaction. Starting from compound 7-keto-LCA (1, 500 mg, 1.28 mmol), compound 2c (254 mg, 47%) was obtained as white solids: R_f_ (TLC) = 0.21 (EtOAc/hexane/AcOH, 50/50/1), mp 112–114 °C (EtOAc), lit. (Une et al., 1989) 102–103°C, [α]_D_^25^ + 32.8 (c 0.27, MeOH). ^1^H NMR (401 MHz, MeOD): δ 3.41 (tt, *J* = 11.2, 4.5 Hz, 1H, H-3), 0.97 (d, *J* = 6.5 Hz, 3H, H-21), 0.91–0.83 (m, 6H, H-19 and H-2′), 0.74 (s, 3H, H-18). ^13^C NMR (101 MHz, MeOD): δ 178.3 (C-24), 76.1 (C-7), 72.8 (C-3), 56.4, 52.6, 45.3, 43.3, 41.6, 40.7, 39.8, 39.4, 37.8, 37.3, 36.9, 36.7, 35.6, 32.3, 32.1, 31.2, 29.3, 27.8, 23.4, 22.6, 19.0, 12.6, 9.9. MS (ESI neg): *m/z* 419.3 (100%, M-H), 465.3 (60%, M+FA-H), 479.3 (44%, M+AcOH−H), 839.6 (75%, 2M−H). HR-MS (ESI neg): *m/z* calcd for C_26_H_43_O_4_ [M−H], 419.31668; found, 419.31647. LCMS method B (ESI−, t_R_ = 14.36 min). Purity 95.6% (HPLC method A, t_R_ = 6.44 min).

3α,7α-Dihydroxy-7β-vinyl-5β-cholan-24-oic acid (7β-vinyl-CDCA, 2d). Compound 2d was prepared according to General Procedure for Grignard Reaction. Starting from compound 7-keto-LCA (1, 500 mg, 1.28 mmol), compound 2d (273 mg, 51%) was obtained as white solids: R_f_ (TLC) = 0.24 (EtOAc/hexane/AcOH, 50/50/1), mp 90–95°C, [α]_D_^25^ + 9.3 (c 0.10, CHCl_3_). ^1^H NMR (401 MHz, CDCl_3_): δ 5.92 (dd, *J*_*1*_ = 17.3 Hz, *J*_*2*_ = 10.7 Hz, 1H, H-1′), 5.15 (dd, *J*_*1*_ = 17.3 Hz, *J*_*2*_ = 1.1 Hz, 1H, (Z)-H-2′), 4.91 (dd, *J*_*1*_ = 10.8 Hz, *J*_*2*_ = 1.0 Hz, 1H, (E)-H-2′), 3.55–3.45 (m, 1H, H-3), 0.96–0.87 (m, 6H, H-19 and H-21), 0.67 (s, 3H, H-18). ^13^C NMR (101 MHz, CDCl_3_): δ 179.3 (C-24), 150.3 (C-1′), 110.2 (C-2′), 75.8 (C-7), 72.1 (C-3), 55.1, 51.2, 43.8, 43.7, 41.8, 41.3, 40.0, 38.7, 35.6, 35.5, 35.2, 34.6, 31.0, 30.9, 30.5, 28.5, 27.9, 22.9, 21.1, 18.5, 12.3. MS (ESI neg): *m/z* 417.3 (80%, M−H), 463.3 (100%, M+FA−H), 477.3 (50%, M+AcOH−H), 835.6 (35%, 2M−H). HR-MS (ESI neg): *m/z* calcd for C_26_H_41_O_4_ [M−H], 417.30103; found 417.30066. LCMS method B (ESI neg., t_R_ = 13.22 min). Purity 96.5% (HPLC method A, t_R_ = 6.00 min).

3α,7α-Dihydroxy-7β-ethynyl-5β-cholan-24-oic acid (7β-ethynyl-CDCA, 2e). Compound 2e was prepared according to General Procedure for Grignard Reaction. Starting from compound 7-keto-LCA (1, 500 mg, 1.28 mmol), compound 2e (370 mg) was obtained as white solids that were re-dissolved in DCM (7 ml). After gentle evaporation with a steam of air, precipitate formed. Filtration afforded solid material that was washed with HPLC grade pentane (3 × 5 ml), dried by high vacuum to obtain 2e as a fine white powder (337 mg, 63%). R_f_ (TLC) = 0.35 (DCM/MeOH, 5/95), mp 122–125°C, [α]_D_^25^ + 48.6 (c 0.15, MeOH). ^1^H NMR (401 MHz, CDCl_3_): δ 3.50–3.37 (m, 1H, H-3), 2.40 (s, 1H, H-2′), 0.92 (d, *J* = 6.6 Hz, 3H, H-21), 0.91 (s, 3H, H-19), 0.69 (s, 3H, H-18). ^13^C NMR (101 MHz, CDCl_3_): δ 177.4 (C-24), 90.7 (C-1′), 71.7 (C-2′), 71.7 (C-7), 69.2 (C-3), 55.2, 50.9, 43.7, 43.5, 42.8, 41.6, 39.8, 38.2, 35.4, 35.3, 34.9, 34.4, 30.9, 30.9, 30.3, 28.4, 26.2, 22.8, 20.9, 18.4, 12.1. MS (ESI neg): *m/z* 415.3 (100%, M−H), 461.3 (10%, M+FA−H). HR-MS (ESI neg): *m/z* calcd for C_26_H_41_O_4_ [M−H], 417.30103; found 417.30066. LCMS method B (ESI neg., t_R_ = 12.27 min). Purity 95.4% (HPLC method A, t_R_ = 5.78 min).

3α,7α-Dihydroxy-7β-propyl-5β-cholan-24-oic acid (7β-propyl-CDCA, 2f). Compound 2f was prepared according to General Procedure for Grignard Reaction. Starting from compound 7-keto-LCA (1, 500 mg, 1.28 mmol), compound 2f (203 mg, 36%) was obtained as white solids. R_f_ (TLC) = 0.26 (DCM/MeOH, 5/95), mp 100–105 °C, lit. (Une et al., 1989) 102–103°C, [α]_D_^25^ + 30.1 (c 0.13, CHCl_3_). ^1^H NMR (401 MHz, CDCl_3_): δ 3.49 (tt, *J*_*1*_= 11.0 Hz, *J*_*2*_ = 4.5 Hz, ^1^H, H-3), 0.94 (d, *J* = 6.4 Hz, 3H, H-21), 0.88 (t, *J* = 6.9 Hz, 3H, H-3′), 0.84 (s, 3H, H-19), 0.70 (s, 3H, H-18). ^13^C NMR (101 MHz, CDCl_3_): δ 179.2 (C-24), 75.4 (C-7), 72.1 (C-3), 54.9, 51.6, 47.6, 44.4, 41.8, 40.5, 40.3, 39.7, 38.8, 36.3, 35.7, 35.5, 34.5, 31.1, 30.9, 30.5, 28.5, 27.1, 22.9, 21.6, 18.6, 18.5, 14.7, 12.4. MS (ESI neg): *m/z* 433.3 (100%, M−H), 479.3 (6%, M+FA−H). HR-MS (ESI neg): *m/z* calcd for C_27_H_45_O_4_ [M−H], 433.33233; found 433.33180. LCMS method B (ESI neg., t_R_ = 16.63 min). Purity 97.8% (HPLC method A, t_R_ = 6.27 min).

3α,7α-Dihydroxy-7β-allyl-5β-cholan-24-oic acid (7β-allyl-CDCA, 2g). Compound 2g was prepared according to General Procedure for Grignard Reaction. Starting from compound 7-keto-LCA (1, 500 mg, 1.28 mmol), compound 2g (220 mg, 40%) was obtained as white solids. R_f_ (TLC) = 0.29 (EtOAc/hexane/AcOH, 50/50/1), mp 90–93°C, [α]_D_^25^ + 42.9 (c 0.11, CHCl_3_). ^1^H NMR (401 MHz, CDCl_3_): δ 5.82 (ddt, *J*_*1*_ = 17.3, *J*_*2*_ = 10.1, *J*_*3*_ = 7.4 Hz, 1H, H-2′), 5.10 (dd, *J*_*1*_ = 10.2, *J*_*2*_ = 2.2 Hz, 1H, (E)-H-3′), 5.04 (dd, *J*_*1*_ = 17.1, *J*_*2*_ = 2.1 Hz, 1H, (Z)-3′), 3.50 (tt, *J*_*1*_ = 11.1, *J*_*2*_ = 4.5 Hz, 1H, H-3), 0.95 (d, *J* = 6.4 Hz, 3H, H-21), 0.82 (s, 3H, H-19), 0.70 (s, 3H, H-18). ^13^C NMR (101 MHz, CDCl_3_): δ 179.0 (C-24), 134.8 (C-2′), 118.6 (C-3′), 74.8 (C-7), 72.1 (C-3), 54.9, 51.7, 48.9, 44.4, 41.7, 41.2, 40.4, 39.8, 38.6, 36.6, 35.7, 35.5, 34.5, 31.0, 30.9, 30.5, 28.5, 27.7, 22.9, 21.6, 18.6, 12.4. MS (ESI neg): *m/z* 431.3 (100%, M−H), 477.3 (50%, M+FA−H), 491.3 (35%, M+AcOH−H), 863.6 (45%, 2M−H). HR-MS (ESI neg): *m/z* calcd for C_27_H_43_O_4_ [M−H], 431.31668; found 431.31629. LCMS method B (ESI neg., t_R_ = 14.92 min). Purity 96.0% (HPLC method A, t_R_ = 8.45 min).

3α,7α-Dihydroxy-7β-isopropyl-5β-cholan-24-oic acid (7β-isopropyl-CDCA, 2h). Compound 2h was prepared according to General Procedure for Grignard Reaction. Starting from compound 7-keto-LCA (1, 500 mg, 1.28 mmol), compound 2h (200 mg, 36%) was obtained as white solids. Crystallization from DCM/MeOH (2 ml/1 drop) afforded 40 mg of crystals (40 mg). R_f_ (TLC) = 0.56 (DCM/MeOH/AcOH, 5/95/1), mp 95–100 °C (DCM:MeOH, 2 ml:1 drop), [α]_D_^25^ + 34.0 (c 0.19, CHCl_3_). ^1^H NMR (401 MHz, CDCl_3_): δ 3.50 (tt, *J*_*1*_ = 11.1, *J*_*2*_ = 4.6 Hz, 1H, H-3), 0.95 (d, *J* = 6.4 Hz, 3H, H-21), 0.89 (d, *J* = 5.5 Hz, 3H, H-2′), 0.87 (d, *J* = 5.5 Hz, 3H, H-2′), 0.83 (s, 3H, H-19), 0.72 (s, 3H, H-18). ^13^C NMR (101 MHz, CDCl_3_): δ 179.3 (C-24), 77.48 (C-7, CDCl_3_ overlap), 72.1 (C-3), 54.8, 51.6, 44.6, 41.2, 40.4, 39.1, 39.1, 36.9, 36.6, 35.8, 35.5, 34.4, 31.8, 31.1, 30.9, 30.5, 28.4, 27.3, 22.9, 21.7, 18.8, 18.6, 16.7, 12.4. MS (ESI neg): *m/z* 433.3 (100%, M−H), 479.3 (4%, M+FA−H). HR-MS (ESI neg): *m/z* calcd for C_27_H_45_O_4_ [M−H], 433.33233; found 433.33195. LCMS method B (ESI neg., t_R_ = 15.70 min). Purity 99.1% (HPLC method A, t_R_ = 7.97 min).

3α,7α-Dihydroxy-7β-cyclopropyl-5β-cholan-24-oic acid (7β-cyclopropyl-CDCA, 2i). Compound 2i was prepared according to General Procedure for Grignard Reaction. Starting from compound 7-keto-LCA (1, 500 mg, 1.28 mmol), compound 2i (185 mg, 33%) was obtained as white solids. R_f_ (TLC) = 0.38 (DCM/MeOH, 10/90), mp 78–82°C, [α]_D_^25^ + 28.3 (c 0.37, CHCl_3_). ^1^H NMR (401 MHz, CDCl_3_): δ 3.54–3.42 (m, 1H, H-3), 0.95 (d, *J* = 6.4 Hz, 3H, H-21), 0.88 (s, 3H, H-19), 0.69 (s, 3H, H-18), 0.58–0.12 (m, 4H, H-2′). ^13^C NMR (101 MHz, CDCl_3_): δ 179.4 (C-24), 72.6 (C-7), 72.1 (C-3), 54.9, 50.8, 45.5, 44.4, 41.8, 40.0, 39.1, 38.8, 35.9, 35.7, 35.4, 34.7, 31.2, 30.9, 30.4, 28.6, 27.5, 24.8, 23.0, 21.3, 18.6, 12.3, 4.5, 2.7. MS (ESI neg): *m/z* 431.3 (65%, M−H), 477.3 (100%, M+FA−H), 491.3 (56%, M+AcOH−H), 863.6 (37%, 2M-H). HR-MS (ESI neg): *m/z* calcd for C_27_H_43_O_4_ [M−H], 431.31668; found 431.31619. LCMS method B (ESI neg., t_R_ = 15.15 min). Purity 96.2% (HPLC method A, t_R_ = 5.74 min).

3α,7α-Dihydroxy-7β-(pent-4-en)-5β-cholan-24-oic acid (7β-pentenyl-CDCA, 2j). Compound 2j was prepared according to General Procedure for Grignard Reaction. Starting from compound 7-keto-LCA (1, 500 mg, 1.28 mmol), compound 2j (198 mg, 34%) was obtained as white solids. R_f_ (TLC) = 0.26 (DCM/MeOH, 5/95), 87–90°C, [α]_D_^25^ + 34.9 (c 0.34, CHCl_3_). ^1^H NMR (401 MHz, CDCl_3_): δ 5.78 (ddt, *J*_*1*_ = 16.9, *J*_*2*_ = 10.1, *J*_*3*_ = 6.7 Hz, ^1^H, H-4′), 5.00 (dq, *J*_*1*_ = 17.2, *J*_*2*_ = 1.7 Hz, ^1^H, (Z)-5′), 4.97–4.93 (m, ^1^H, (E)-5′), 3.55–3.43 (m, ^1^H, H-3), 0.94 (d, *J* = 6.4 Hz, 3H, H-21), 0.84 (s, 3H, H-19), 0.70 (s, 3H, H-18). ^13^C NMR (101 MHz, CDCl_3_): δ 179.5 (C-24), 138.8 (C-4′), 114.9 (C-5′), 75.3 (C-7), 72.1 (C-3), 54.9, 51.6, 44.5, 44.4, 41.7, 40.7, 40.4, 39.6, 38.8, 36.4, 35.7, 35.5, 34.5, 34.4, 31.2, 30.9, 30.5, 28.5, 27.3, 24.6, 23.0, 21.6, 18.6, 12.5. MS (ESI neg): *m/z* 459.3 (60%, M−H), 505.4 (100%, M+FA−H), 519.4 (47%, M+AcOH−H), 919.7 (45%, 2 M−H). HR-MS (ESI neg): *m/z* calcd for C_29_H_47_O_4_ [M-H], 459.34798; found 459.34770. LCMS method B (ESI neg., t_R_ = 17.73 min). Purity 99.4% (HPLC method A, t_R_ = 4.31 min).

3α,7α-Dihydroxy-7β-nonyl-5β-cholan-24-oic acid (7β-nonyl-CDCA, 2k). Compound 2k was prepared according to General Procedure for Grignard Reaction. Starting from compound 7-keto-LCA (1, 500 mg, 1.28 mmol), compound 2k (235 mg, 35%) was obtained as white solids. R_f_ (TLC) = 0.27 (DCM/MeOH, 5/95), mp 78–80°C, [α]_D_^25^ + 30.6 (c 0.36, CHCl^3^). ^1^H NMR (401 MHz, CDCl_3_): δ 3.49 (tt, *J*_*1*_ = 11.0, *J*_*2*_ = 6.2 Hz, 1H, H-3), 0.94 (d, *J* = 6.4 Hz, 3H, H-21), 0.87 (t, *J* = 6.5 Hz, 3H, H-9′), 0.84 (s, 3H, H-19), 0.70 (s, 3H, H-18). ^13^C NMR (101 MHz, CDCl_3_): δ 179.2 (C-24), 75.4 (C-7), 72.1 (C-3), 54.9, 51.6, 45.1, 44.4, 41.8, 40.5, 40.4, 39.7, 38.8, 36.4, 35.7, 35.5, 34.5, 32.1, 31.1, 31.0, 30.5, 30.3, 29.7, 29.4, 28.5, 27.2, 25.2, 23.0, 22.8, 21.6, 18.6, 14.2, 12.4. MS (ESI neg): *m/z* 517.4 (58%, M−H), 563.4 (100%, M+FA−H), 577.4 (60%, M+AcOH−H), 1035.9 (69%, 2M−H). HR-MS (ESI neg): *m/z* calcd for C_33_H_57_O_4_ [M−H], 517.4262; found 517.4258. LCMS method B (ESI neg., t_R_ = 25.75 min). Purity 97.9% (HPLC method A, t_R_ = 4.36 min).

## Results

### Library Synthesis

**FIGURE 2:**
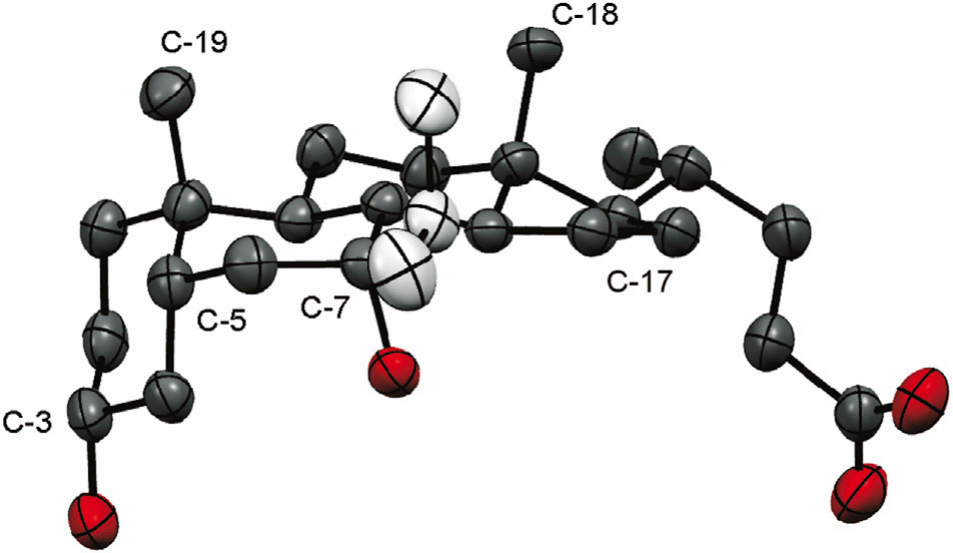
An ORTEP (Farrugia, 2012) view of 2 h (7β-isopropyl-CDCA), displacement ellipsoids are shown with 50% probability. Oxygen – red, steroid carbon skeleton – black, isopropyl moiety – white.

7-Ketolithocholic acid (1) was prepared by a three-step synthesis (Figure 1) from commercially available chenodeoxycholic acid (CDCA). First, the carboxylic moiety was protected as methyl ester, followed by the selective oxidation of a 7-hydroxy substituent (Stefela et al., 2020). The regioselectivity of the oxidation towards the C-7 substituent is given by the different reactivity of C-3 equatorial and C-7 axial hydroxy groups, which has been described in the literature (Haslewood, 1942; Fieser and Rajagopalan, 1949; Fieser and Rajagopalan, 1950). The protection of carboxylic moiety as ester facilitates the separation of products after the oxidation step on the column chromatography. Finally, the ester moiety was hydrolyzed under basic conditions.

(E)-7-Ethylidene derivative (7-ELCA, 7-ethylidene-LCA, 2a) was prepared by the Wittig reaction using ethyltriphenylphosphonium bromide as an alkylating reagent (Posa et al., 2014). Similar to the published data, only the *E*-isomer was obtained. Its structure was confirmed by ROESY NMR (Supplementary Figure S9), exhibiting contacts of the double-bond hydrogen with hydrogen atoms in positions C-14 and C-15. Next, compounds 2b-2k were prepared by the addition of Grignard reagent on the C-7 carbonyl group. The addition proceeded exclusively from the β-side of the steroid skeleton to form a new equatorial C-C bond, as reported by other groups (Une et al., 1989; Bjedov et al., 2017). The stereochemistry at C-7 was assigned and confirmed by several ROESY NMR experiments. For example, the olefinic CH protons of 2d and 2g had clear contacts to hydrogen atoms in position C-6β and C-8, which confirms that the allyl substituent is in position C-7β and the hydroxyl group in position C-7α (Supplementary Figures S10, S11). The structure of 7-ethyl derivative 2c does not exhibit such clear contacts of 7-substituent with the steroid skeleton. The structure was confirmed by the catalytic hydrogenation on palladium in ethanol of 7β-vinyl derivative 2d that afforded compound with an identical ^1^H NMR spectrum with that of compound 2c. Finally, the crystal data (Figure 2) of compound 2h (7β-isopropyl-CDCA) showed an alkyl substituent in position C-7β and a hydroxyl group in position C-7α.

### FXR Agonistic and Antagonistic Activities of 7-Alkylated Derivatives

**FIGURE 3:**
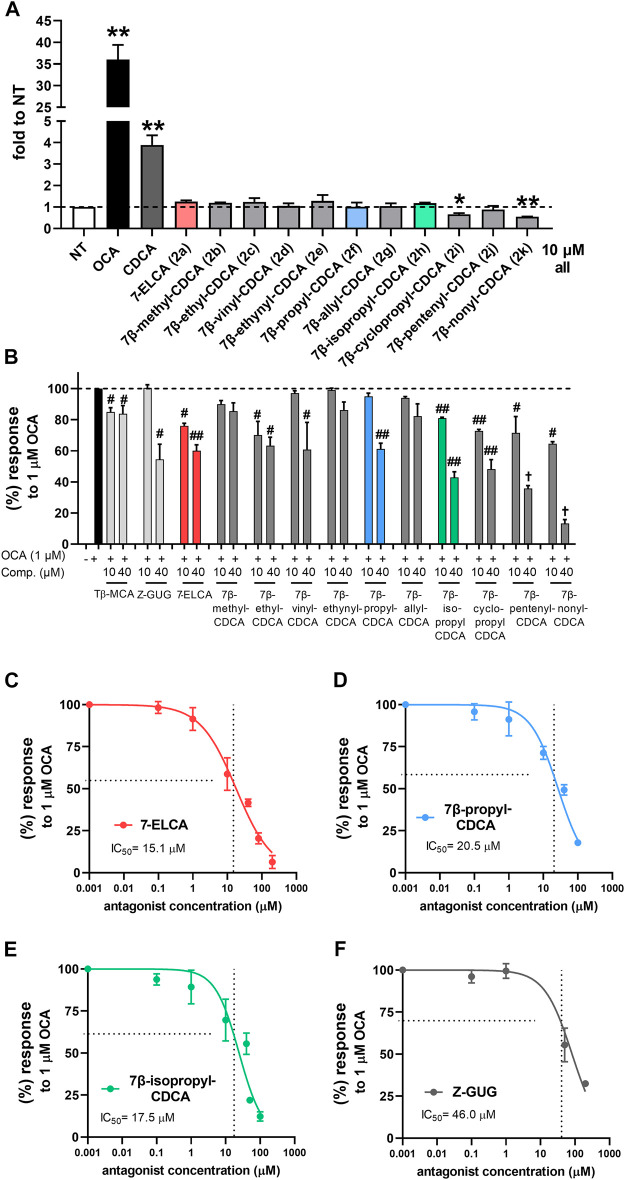
Interaction of 7-alkylated derivatives of CDCA and 7-ELCA with human FXR in luciferase reporter gene assays. HepG2 cells were transiently co-transfected with the luciferase FXRE-luc construct and with expression pSG5-hFXR, pSG5-hRXRα, and pRL-TK vectors. Cells were treated with indicated concentrations for 24 h alone **(A)**, *an agonistic mode)* or with 1 µM OCA as an FXR agonist **(B–F)**, *an antagonistic mode)*. FXR agonist CDCA and antagonists Tβ-MCA and Z-GUG were used as known FXR ligands. Data were normalized to *Renilla* luciferase activity and are expressed relative to those of control nontreated cells (NT). Values are presented as the means ± SD of three independent experiments performed in biological triplicates (*n* = 3). **p < 0.01 vs. NT; ^#^ *p* < 0.05, ^##^ *p* < 0.01 vs. 1 µM OCA. IC_50_ values were calculated using concentration-response curves nonlinear fitting in GraphPad software.

To determine the activity of novel derivatives on FXR, we performed luciferase gene reporter assays using a human FXR expression construct in human hepatocyte-derived HepG2 cells. We found that the alkyl substitution to the 7β position led to the complete abrogation of the capacity to activate FXR for all derivatives at 10 µM concentration (Figure 3A). Moreover, the introduction of cyclopropyl (2i) and nonyl (2k) moieties resulted in significant inhibition of the FXR basal activation.

Subsequently, we evaluated whether the introduction of alkyl substituents to the C-7β position would result in an antagonistic capacity toward FXR. For this purpose, we co-treated HepG2 cells with tested compounds together with different known FXR agonists including the highly potent semisynthetic bile acid OCA (1 μM, Figure 3B), non-steroidal ligand GW4064 (1 µM) or endogenous bile acid CDCA (20 μM, Supplementary Table S1). Our results show that the FXR antagonizing behavior appears to be dependent on the length and level of unsaturation of the alkyl substituent at the 7β position. The FXR antagonizing capacity increased with the longer alkyl chain: methyl (2b) < ethyl (2c) < propyl (2f) derivative (Figure 3B). Furthermore, the FXR antagonizing capacity was improved for the branched isopropyl (2h) and cyclopropyl (2i) analogs as compared with the propyl (2f) derivative (Figure 3B). Longer substituents (≥ C_5_, 2j, 2k) may, however, affect viability at higher concentrations, as the IC_50_ of 7β-nonyl derivative (2k) was determined about 15 µM in various cell lines using the MTS viability assay (Supplementary Table S2). This effect might relate to the increased lipophilicity of compounds 2j and 2k. Contrarily, compounds 2a-2h exhibited no effects on cellular viability with IC_50_ value > 100 µM (Supplementary Figure S1, Supplementary Table S2).

Interestingly, the ability of vinyl (2d), allyl (2g), and pent-4-enyl (2j) derivatives to antagonize the OCA-stimulated FXR activation was maintained with the presence of a double bond in the alkyl moiety. However, this antagonizing capacity might be dependent on the double bond position as well as the spatial orientation of the lipophilic moiety, as 7-ELCA (2a) with (E)-7-ethylidene substituent was established as the most potent FXR antagonist. On the other hand, the compound 2e bearing ethynyl substituent failed to maintain any strong antagonistic effect and exhibited only minor antagonist activity.

In summary, 7-ELCA (2a), 7β-propyl-CDCA (2f), and 7β-isopropyl-CDCA (2h) were demonstrated as the most potent FXR antagonists. These compounds inhibited FXR activation in a concentration-dependent manner by about 95%, 85%, and 90% at 100 µM concentration, respectively, with IC_50_ values of 15.1 ± 0.7 µM (7-ELCA, 2a, Figure 3C), 20.5 ± 1.0 µM (7β-propyl-CDCA, 2f, Figure 3D) and 17.5 ± 1.7 µM (7β-isopropyl-CDCA, 2h, Figure 3E), respectively. Known FXR antagonists tauro-β-muricholic acid (Tβ-MCA) and Z-GUG were used as controls in these experiments. However, their FXR antagonistic capacity to inhibit FXR activation by the highly potent agonist OCA was limited, and Tβ-MCA decreased the FXR activity by 15% and Z-GUG by 70% at the concentration of 100 μM (IC_50_ for Z-GUG = 46.0 ± 7.1 μM, Figure 3F).

### Interaction of 7-ethylideneLCA and 7β-isopropyl-CDCA Within the Ligand Binding Pocket of the Farnesoid X Receptor Receptor

**FIGURE 4:**
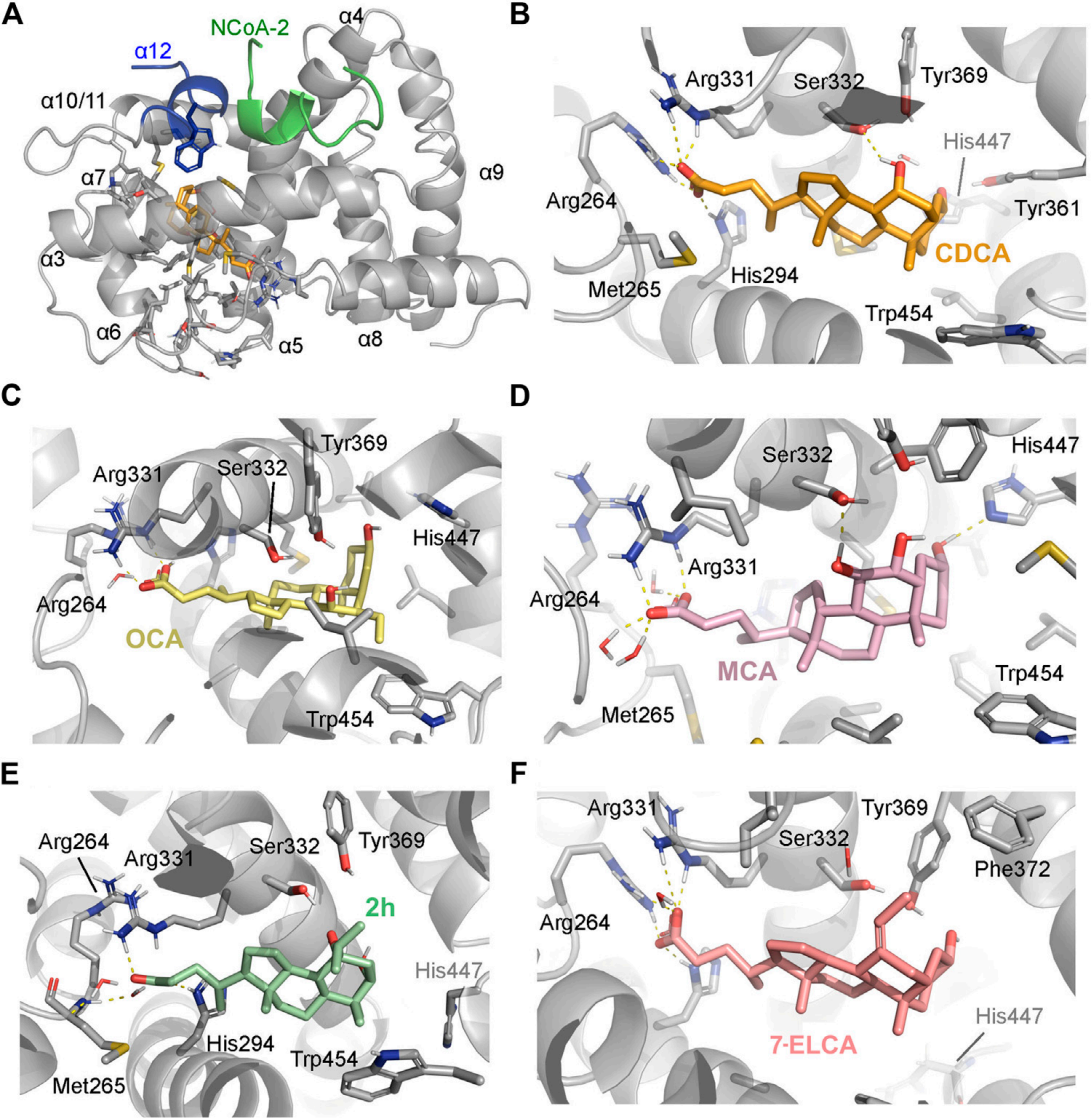
**(A)** Ligand binding domain of human FXR, bound to the co-activator peptide (NcoA-2/SRC-2, in green) on the AF-2 cavity, composed by the α-12. All helices mentioned in the text are numbered α1–12. Representative snapshots from the molecular dynamics simulation (500 ns per system), highlighting the residues involved in interactions: **(B)** CDCA (PDB ID: 6HL1), **(C)** OCA, **(D)** muricholic acid (MCA), **(E)** 7β-isopropyl-CDCA (2h) and **(F)** 7-ELCA (2a). Interactions are represented by dashed lines. FXR residues are colored according to the atom types of the interacting amino acid residues (protein’s carbon, light grey; nitrogen, blue; oxygen, red).

The FXR LBD structure with the co-crystallized CDCA and OCA, as well as docked poses for 7-ELCA and 7β-isopropyl-CDCA, underwent short molecular dynamics simulations to understand how they could potentially interact within the ligand binding pocket (LBP). Information from different FXR crystal structures shows that the positions of most α-helices are conserved for steroid-based ligands, with the exception of helices α11 and α12, which suggests a single flexible LBP. Due to the high chemical similarity between our series and co-crystallized ligands, we postulated that all ligands could occupy a similar binding site (respective side-chains are depicted as sticks in Figure 4A). However, crystal structures and docking poses can only represent a static snapshot of this interaction, which drove us to use simulations as a model to represent the dynamic equilibrium. CDCA (Figure 4B) and OCA (Figure 4C) have conserved electrostatic interactions between the carboxylate moieties His294, Arg331 and Arg264, the latter leading to the reorganization of the loop between helices α1 and α2. Additionally, both OCA and CDCA presented recurrent hydrogen interactions between the 7α-hydroxyl’s group and Ser332 and Tyr369, which were less prominent in our antagonists. The work from Merk *et al.* describes the lipophilic contact between the Trp454 and the hydrophobic β-face of CDCA’s A-ring as crucial for FXR full activation (Merk et al., 2019). Similar hydrophobic contact was conserved for most of our proposed antagonists (Supplementary Figure S8B), with exception of guggulsterone (Supplementary Figure S8C), which was unstable in our simulations. Trp454 interaction can also bring the 3α-hydroxyl group into a suitable position to interact with Tyr361 and His447. However, interactions between the 3α-hydroxyl group and Tyr361/His447, although represented in the crystal structure, were not conserved in simulations (Supplementary Figure S8A and Zenodo open data repository under the doi:10.5281/zenodo.3898392).

Simulations of the antagonist muricholic acid’s (MCA) docking pose (Figure 4D) generated a similar interaction profile with a more stable interaction with His447 (Supplementary Figure S9A). We hypothesize that the free His447 could influence the conformation of the α11 and so the heterodimerization interface. However, the extent of this conformational change would need to be addressed by longer monomeric simulations and simulations with the heterodimer. Contrastingly, our proposed antagonists 7-ELCA (2a) and 7β-isopropyl-CDCA (2h) shared similar features with OCA and CDCA, such as a stable interaction with the loop L:α1–α2’s residues (Figure 4E), which suggests a competitive mechanism of action against the natural ligand. Specifically, 7-ELCA had no interactions with Ser332 and Tyr361. Due to the lack of 7α-hydroxyl’s group (Figure 4F), the counterpart ethylidene moiety established hydrophobic contacts with both Phe366 and Phe372.

### 7-ELCA, 7β-Propyl-CDCA and 7β-Isopropyl-CDCA Are Farnesoid X Receptor Antagonists in the TR-FRET FXR Co-Activator Assay With the Recombinant Farnesoid X Receptor LBD

**FIGURE 5:**
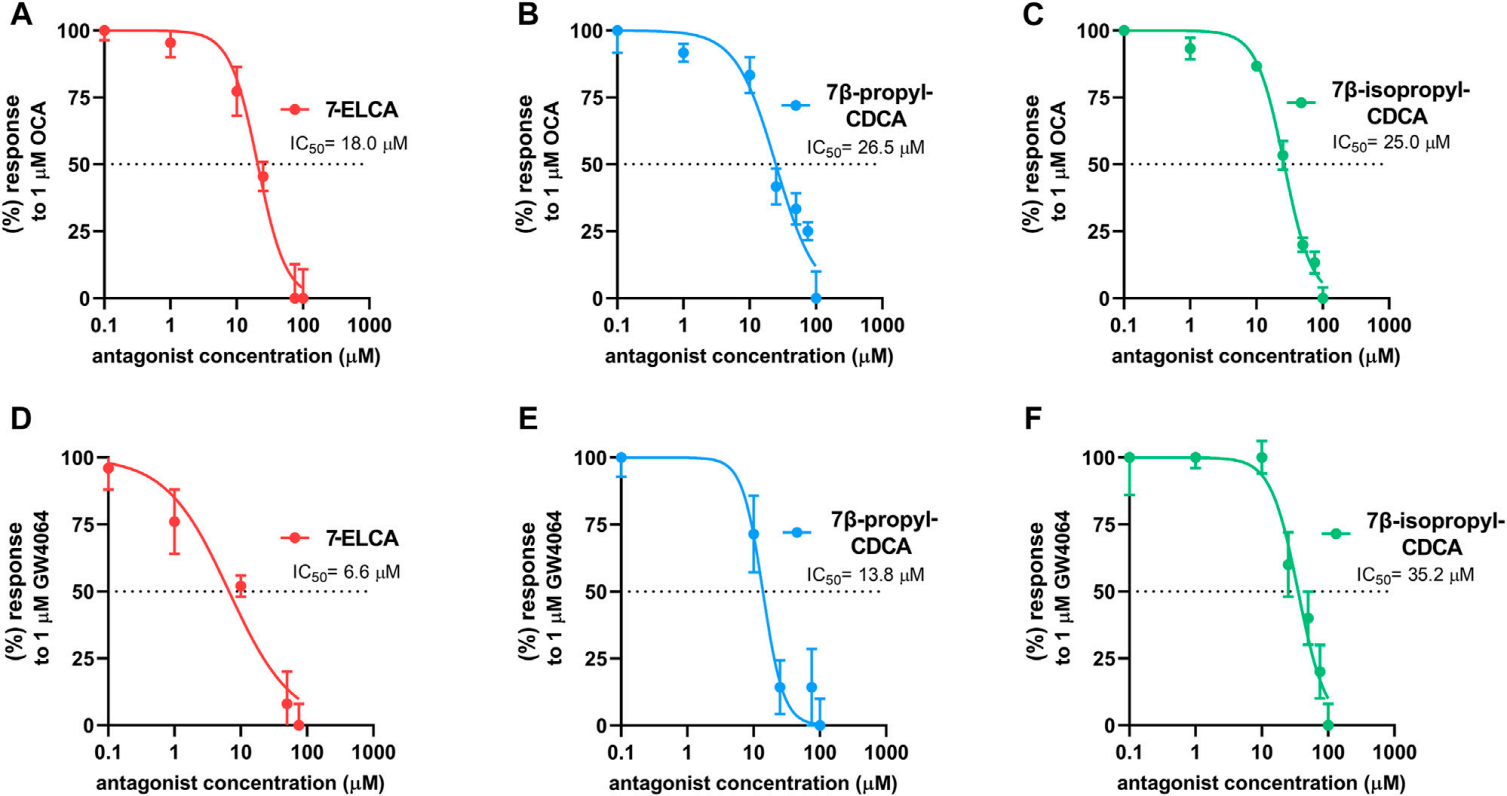
7-ELCA, 7β-propyl-CDCA and 7β-isopropyl-CDCA inhibit, in a concentration-dependent manner, the interaction of the FXR LBD stimulated by OCA (1 μM, **(A–C)** or GW4064 (1 μM, **(D–F)** with SRC-2 co-activator peptide in the TR-FRET FXR Coactivator assay. IC_50_ values were calculated from three independent experiments performed in four replicates using concentration-response curves nonlinear fitting (log(inhibitor) vs. normalized response) in GraphPad software.

The time-resolved fluorescence energy transfer (TR-FRET) FXR co-activator association assay with the recombinant FXR LBD was used to assess the FXR antagonism of the most potent FXR antagonists 7-ELCA, 7β-propyl-CDCA and 7β-isopropyl-CDCA in the cell-free system. We found that all three compounds inhibited OCA-induced recruitment of the co-activator peptide SRC-2 to FXR LBD in a concentration-dependent manner with IC_50_ values 18.0 ± 2.7 µM (7-ELCA), 26.5 ± 2.0 µM, (7β-propyl-CDCA), 25.0 ± 3.7 µM (7β-isopropyl-CDCA, Figures 5A–C), respectively. Moreover, the derivatives antagonized the interaction of SRC-2 and FXR promoted by the nonsteroidal FXR activator GW4064 with IC_50_ values 6.6 ± 2.9 µM (7-ELCA), 13.8 ± 2.5 µM, (7β-propyl-CDCA), 35.2 ± 5.4 µM (7β-isopropyl-CDCA, Figures 5D–F), respectively.

### Regulation of the Farnesoid X Receptor Target Gene Expression by 7-ELCA and 7β-propyl-CDCA in Hepatic Cells

**FIGURE 6:**
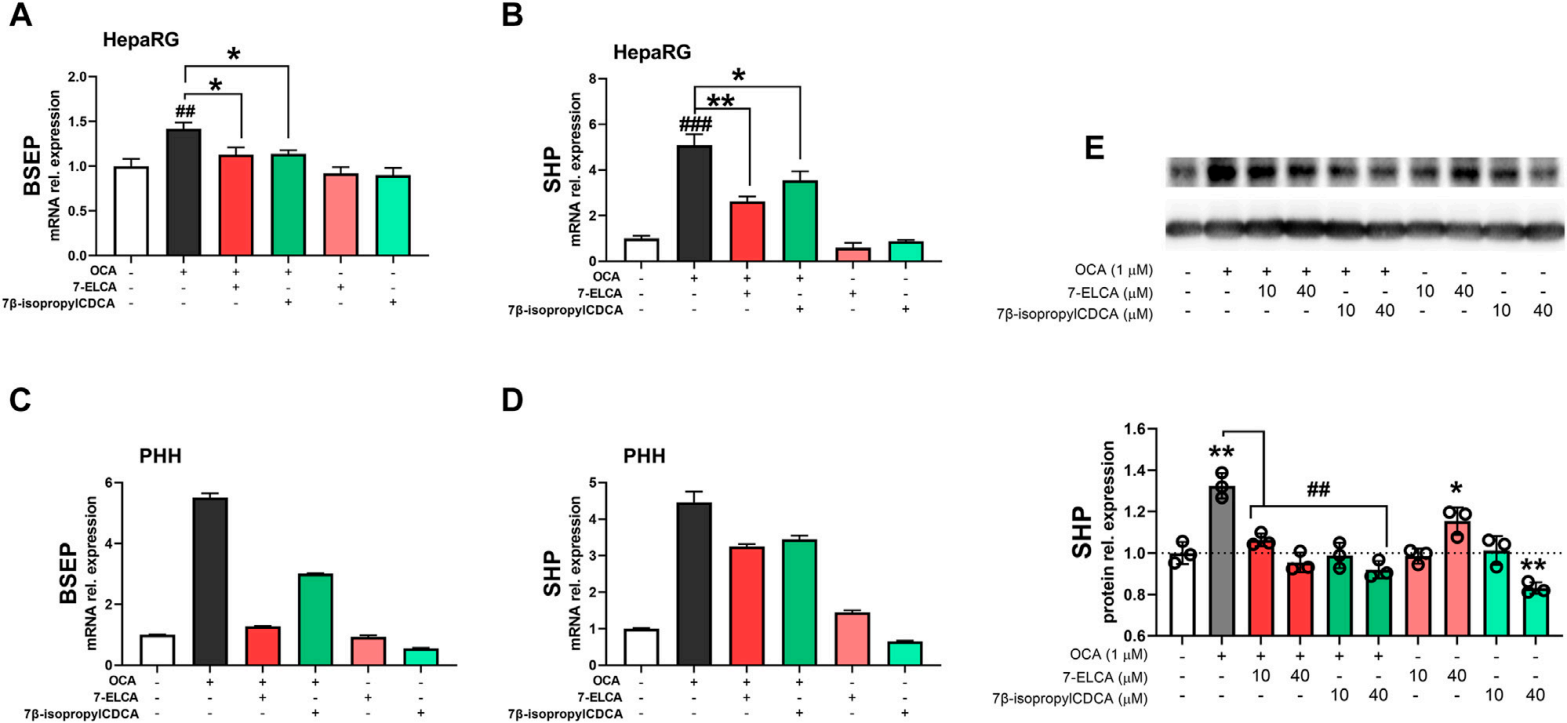
Regulation of FXR downstream genes by 7-ELCA and 7β-propyl-CDCA in the presence or absence of 1 µM OCA as the potent FXR agonist. Real-time qPCR analysis of mRNA expression of **(A,C)** BSEP and **(B,D)** SHP genes was performed in terminally differentiated HepaRG hepatic cells **(A,B)**, and in primary human hepatocytes (PHH) from one donor treated for 24 h **(C,D)**. **(E)** SHP protein expression in HepaRG cells after 48 h treatment with OCA alone or in combination with 7-ELCA and 7β-propyl-CDCA. Data were normalized to B2M mRNA or GAPDH protein and are expressed relative to those of control (vehicle-treated) cells. The experiments were repeated in three independent experiments performed in triplicates and values are presented as the means ± SD. **p < 0.01, ***p < 0.001 vs. NT; ^#^ *p* < 0.05, ^##^ *p* < 0.01 vs. 1 µM OCA.

To further corroborate the FXR antagonistic properties of 7-ELCA and 7β-propyl-CDCA, we examined their effects on the expression of FXR downstream genes in the presence and absence of the FXR agonist OCA in terminally differentiated HepaRG cells (Figures 6A,B) and primary human hepatocytes (Figures 6C,D). 7-ELCA and 7β-propyl-CDCA (40 µM) reduced BSEP (Figures 6A,C) and SHP (Figures 6B,D) mRNA levels upregulated by OCA (1 µM). The treatment with 7-ELCA and 7β-propyl-CDCA, *per se*, did not have an impact on the expression of FXR target genes BSEP and SHP mRNA. In addition, we confirmed the FXR antagonistic effect of 7-ELCA and 7β-propyl-CDCA on SHP protein expression in HepaRG cells treated with the FXR agonist OCA (Figure 6E). The effects of 7-ELCA and 7β-propyl-CDCA were much stronger on SHP protein downregulation than on SHP mRNA expression after treatment with OCA in HepaRG cells (Figures 6B,E). We suppose that the phenomenon is due to the longer treatment intervals in protein expression experiments. Altogether, these data suggest that 7-ELCA and 7β-propyl-CDCA act as FXR antagonists in hepatic cells.

### 7-ELCA Does Not Interact With Other Nuclear Receptors

**FIGURE 7:**
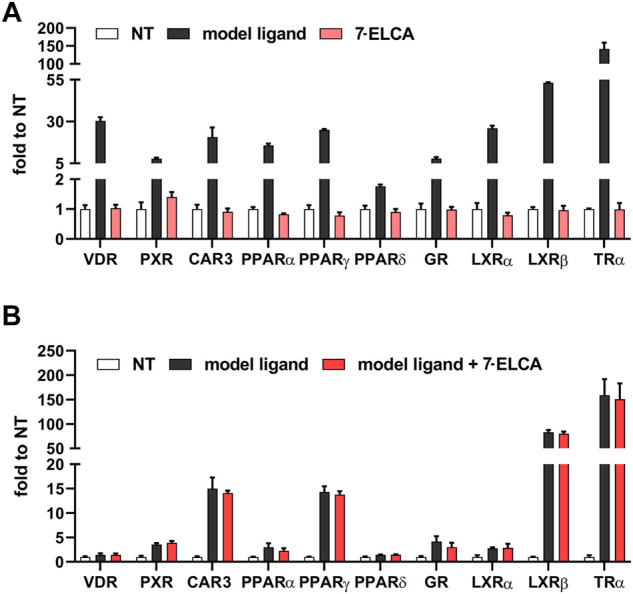
Specificity of 7-ELCA in agonistic **(A)** and antagonistic **(B)** assays performed on transiently co-transfected HepG2 cells with luciferase reporter constructs in combination with appropriate expression vectors as indicated in *Materials and Methods*. **(A)** Cells were treated with 7-ELCA for 24 h. The following compounds were used as model ligands: VDR agonist 1α,25(OH)_2_vitaminD_3_ (100 nM), PXR agonist (rifampicin 10 µM), human CAR agonist (CITCO 10 µM), PPARα agonist fenofibrate (10 µM), PPARγ agonist rosiglitazone (10 µM), PPARδ agonist GW501516 (10 µM), GR agonist dexamethasone (100 nM), LXRα and LXRβ agonist GW3964 (10 µM), and TR agonist thyroxin (10 µM). **(B)** The same model ligands of the nuclear receptors were used in combination with 7-ELCA in the same assays; VDR agonist 1α,25(OH)_2_vitaminD_3_ (10 nM), PXR agonist rifampicin (1 µM), human CAR agonist CITCO (1 µM), PPARα agonist fenofibrate (1 µM), PPARγ agonist rosiglitazone (1 µM), PPARδ agonist GW501516 (1 µM), GR agonist dexamethasone (50 nM), LXRα and LXRβ agonist GW3965 (1 µM) and TR agonist thyroxin (1 µM) were used. Data were normalized to *Renilla* luciferase activity and are expressed as fold activation relative to control (nontreated, NT) cells. Values are presented as the means ± SD of three independent experiments.

To examine the specificity of the most potent FXR antagonist in this study, 7-ELCA, we assessed its interaction with a wide range of nuclear receptors known to interact with BAs or to regulate metabolic processes, including vitamin D receptor (VDR), pregnane X receptor (PXR), constitutive androstane receptor (CAR), peroxisome proliferator-activated receptors α, γ, β/δ (PPAR α, γ, β/δ), glucocorticoid receptor (GR), liver X receptor α, β (LXR α, β) and thyroid receptor (TRα). As shown in Figure 7A, 7-ELCA did not activate any of the examined nuclear receptors. In addition, 7-ELCA did not antagonize the activation of the nuclear receptors stimulated by their model ligands at the 10 µM concentration (Figure 7B). Taken together, 7-ELCA is a selective FXR antagonist when considering interactions with the tested nuclear receptors.

### 7-ELCA Is a Potent Agonist of G-Protein Bile Acid Receptor 1

**FIGURE 8:**
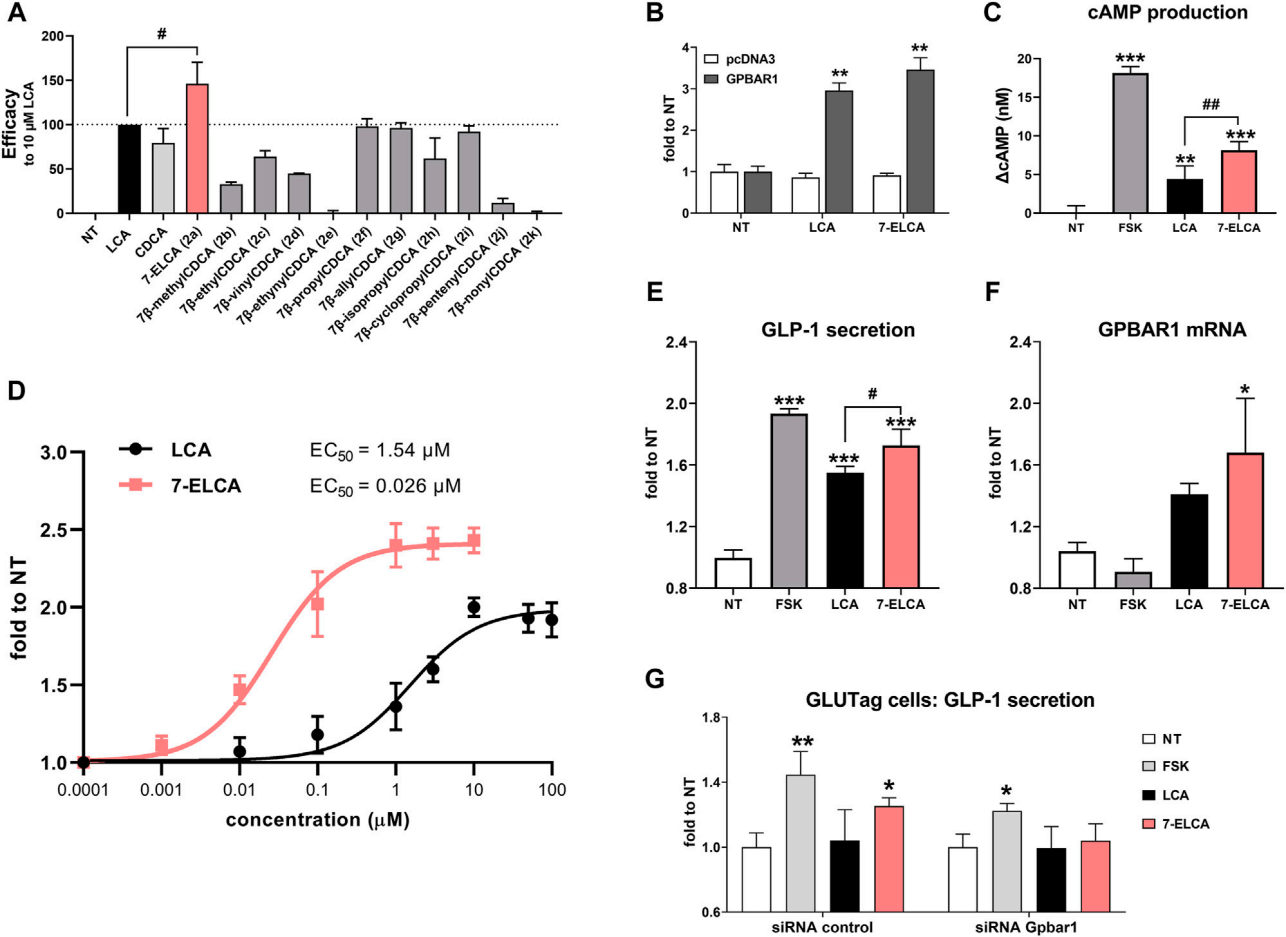
Effects of 7-alkylated derivatives of CDCA and 7-ELCA on the membrane receptor GPBAR1. **(A, B, D)** HepG2 cells were co-transfected with a CRE-containing luciferase reporter plasmid and a GPBAR1 expression vector and cells were then treated with tested compounds at 10 µM (*A,B*) or with increasing concentrations **(D)** for 5 h. **(A)** Efficacy of tested compounds to activate CRE-luc was compared to the activity of 10 µM LCA (set as 100% activation). **(B)** CRE-luc activation depends on the presence of GPBAR1. **(C)** Effects of LCA and 7-ELCA (both at 10 µM) on intracellular generation of cAMP in differentiated NCI-H716 cells after 30 min treatment. The increase in cAMP level (ΔcAMP) is presented as the difference between cAMP concertation in the treated sample after subtraction of the cAMP in the nontreated control sample. **(D)** Concentration-response curves for activation of GPBAR1 by LCA and 7-ELCA. EC_50_ values were calculated using nonlinear fitting of concentration-response curves. **(E)** 7-ELCA and LCA (both 10 µM) stimulate GLP-1 secretion and **(F)** GPBAR1 mRNA expression in differentiated human enteroendocrine NCI-H716 cells after 2 h treatment. **(G)** 7-ELCA (10 µM) increases GLP-1 secretion in siRNA control but not in siRNA Gpbar1 transfected GLUTag cells after 1 h treatment. GLP-1 was normalized to protein concentration and GPBAR1 mRNA expression to GAPDH mRNA. Data are presented as a fold to control nontreated (NT) samples. Forskolin (FSK, 10 µM) was used as a GPBAR1-independent activator of cAMP production. Values are presented as means ± SD from three independent experiments performed in triplicates. **p < 0.01, ***p < 0.001 vs. NT; ^#^ *p* < 0.05, ^##^ *p* < 0.01 vs. 10 µM LCA.

Upon the activation of the membrane GPBAR1, BAs bind to the ligand-binding pocket of GPBRA1 and trigger downstream signaling via cAMP generation followed by the activation of downstream kinases and cAMP response element (CRE) in the nucleus (Kawamata et al., 2003). Tested compounds (10 µM) were analyzed for their ability to activate a CRE-luc construct when compared to LCA (10 µM) as a known GPBAR1 agonist (Figure 8A). Only 7-ELCA (2a) was able to significantly increase CRE-luc activation more than LCA (10 µM). 7β-propyl-CDCA (2f), 7β-allyl-CDCA (2g) and 7β-cyclopropyl-CDCA (2i) displayed comparable activities to LCA. Other compounds significantly activated GPBAR1 with activity lower than LCA at the concentration of 10 μM and compounds 7β-ethynyl-CDCA (2e), 7β-pentenyl-CDCA (2j) and 7β-nonyl-CDCA (2k) exhibited weak or no ability to act as agonists of GPBAR1. As demonstrated in Figure 7B, the activation of CRE-luc by LCA or other compounds was dependent on the co-transfection of GPBAR1, because the mock co-transfection of an empty vector did not lead to augmented luciferase activity of CRE-luc. Consistently with the data, the treatment with 10 µM 7-ELCA led to more significant production of cAMP compared to the treatment with 10 µM LCA in differentiated enteroendocrine NCI-H716 cells (Figure 8C).

The concentration-response study (Figure 8D) underlined the superiority of 7-ELCA in GPBAR1 activation with the EC_50_ value being lower by about 2 orders of magnitude when compared to LCA activity (0.026 ± 0.006 µM vs. 1.54 ± 0.4 µM, respectively).

The activation of GPBAR1 by BAs in colonic L-cells is known to result in the secretion of the incretin GLP-1, which in turn stimulates insulin secretion from pancreatic cells. To further evaluate the activity of 7-ELCA on GPBAR1, we exposed differentiated colonic human NCI-H716 L-cells to 7-ELCA. We observed a significant increase of GLP-1 secretion into the culture media after the treatment with 7-ELCA (Figure 8E). In addition, the GLP-1 secretion induced by 7-ELCA (10 µM) was significantly stronger compared to LCA (10 µM). Interestingly, 7-ELCA can also increase GPBAR1 mRNA expression in NCI-H716 cells (Figure 8F). We determined GLP-1 secretion in control and Gpbar1 siRNA transfected GLUTag cells. The treatment of 7-ELCA (10 µM) led to a significant increase of the GLP-1 secretion in the control cells. However, when the Gpbar1 expression was silenced by Gpbar1 siRNA, 7-ELCA treatment did not lead to the significantly increased GLP-1 production (Figure 8G). To summarize, our results show that 7-ELCA is a potent steroidal GPBAR1 agonist with an EC_50_ value at nanomolar concentration and its effect on GLP-1 production is dependent on the GPBAR1 expression.

### Interactions of LCA and 7-ELCA With the Ligand Binding Pocket of G-Protein Coupled Bile Acid Receptor-1

**FIGURE 9:**
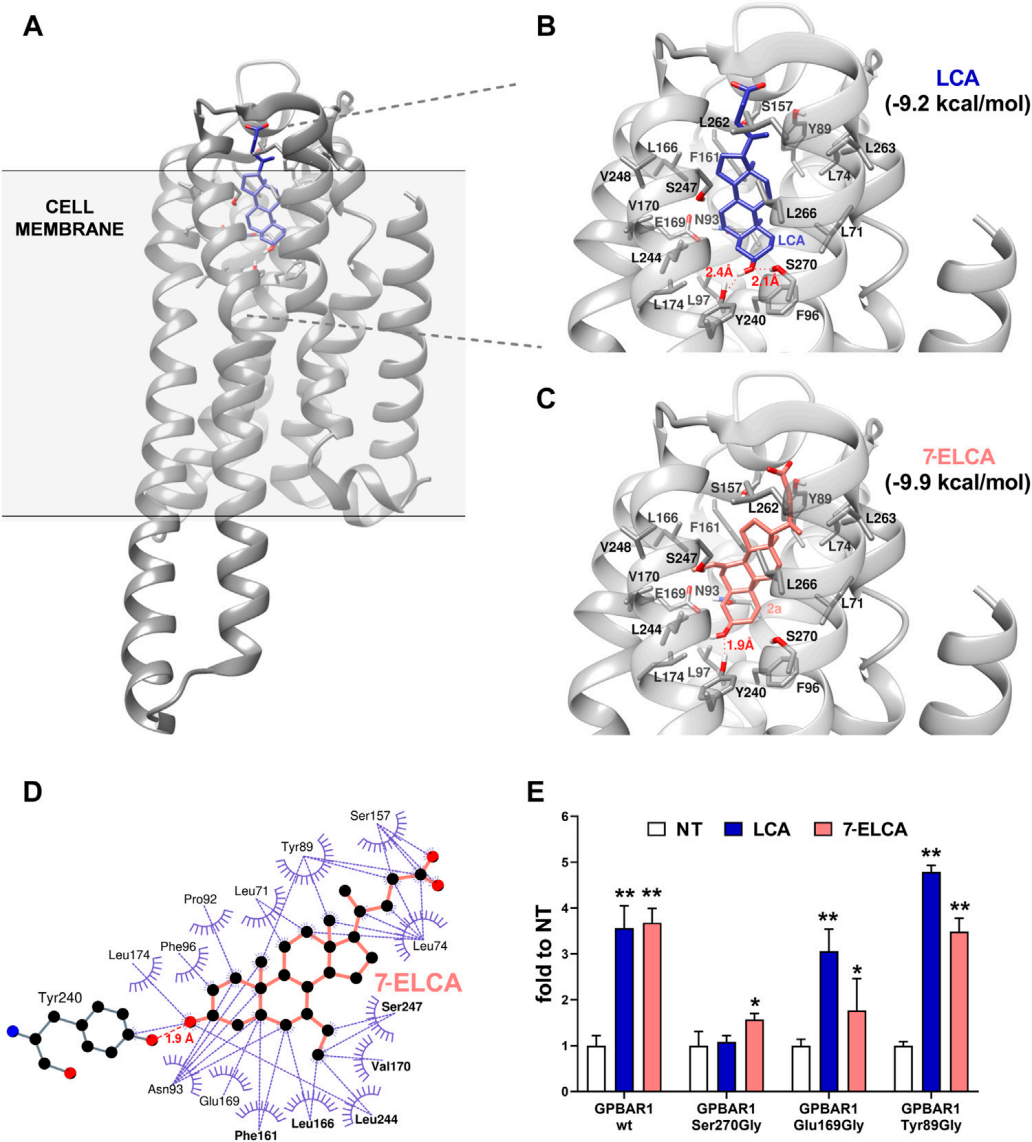
Interactions of LCA and 7-ELCA in the ligand binding pocket of GPBAR1. **(A)** The position of the model ligand LCA is perpendicular to the cell membrane with A-ring facing the bottom of the LBD. Detailed presentation of LBD with LCA **(B)** and 7-ELCA **(C)** in the LBD of GPBAR1. Docking was carried out using AutoDock Vina 1.1.2. software. **(D)** A 2D representation of molecular interactions between 7-ELCA and GPBAR1 as generated in LigPlot+. The dashed line in red represents the hydrogen bond between C-3 substituent of 7-ELCA and Tyr240. Dashed lines in purple represent hydrophobic interactions. Amino acid residues forming the hydrophobic pocket are presented in bold. **(E)** HepG2 cells were co-transfected with CRE-luc together with the wild type (*wt*) GPBAR1 or its mutated variants Ser270Gly, Glu169Gly or Tyr89Gly as indicated. Luciferase activity was normalized to the *Renilla* luciferase as an internal control. Data are expressed as means ± SD from three independent determinations performed in triplicates. **p < 0.01, ***p < 0.001 vs. NT.

For a long time, only homology models of GPBAR1 have been available. Recently, the cryo-electron microscopy structure of GPBAR-G_s_ unveiled an oval LBP with hydrophilic residues accumulated at the bottom part of the cavity with the rest of the LBD surface formed predominantly by hydrophobic amino acids (Yang et al., 2020). For this study, the PDB 7CFN model was used due to the structural similarity of our compounds and the bound ligand (6-ethyl-23(*S*)-methyl-cholic acid, INT-777). The molecular docking study revealed that LCA binds to GPBAR1 perpendicularly to the cytoplasmatic membrane (Figure 9A). The A-ring of LCA faces the hydrophilic bottom of the cavity where it forms a hydrogen bond between 3-hydroxyl group and amino acid residues Tyr240 and Ser270. The rest of the LBD is fairly hydrophobic and further stabilizes the LCA preferred pose. The flexible sidechain connected to the D-ring and ended by a carboxyl group freely floats in the outward direction from the pocket. However, it is not long enough to exhibit any interaction with polar groups that form the outer surface around the pocket entrance.

The docking study showed that the docking score of LCA to GPBAR1 (−9.2 kcal/mol, Figure 9B) is worse than in the case of 7-ELCA (−9.9 kcal/mol, Figure 9C). In addition, 7-ELCA had the best docking score towards GPBAR1 among all alkylated derivatives (Supplementary Figure S2). All tested ligands adopted a similar position as LCA in the LBD. If present, the C-7 hydroxyl always forms a hydrogen bond with Ser247. The C-7 alkyls face towards a strongly hydrophobic pocket cleft formed by Phe161, Leu166, Val170, Leu244, Ser247, and Val248, as presented with the 7-ELCA in Figure 9D. The alkylation at the position C-7 might help ligands to pose in the pocket tightly. Furthermore, the alkylation on C-7 influences the hydrogen bond formation between a ligand and Ser270. Ligands with two-carbons substituents on C-7 form only one hydrogen bond interaction between the C-3 hydroxyl and Tyr240. The Ser270 hydroxyl is spatially too far away because the whole ligand’s C-7 two-carbons substituent drags the ligand towards the hydrophobic pocket cleft to exploit hydrophobic interactions. On the other hand, compounds with three-carbons substituents are wide enough to reach both the hydrophobic pocket cleft with its C-7 substituent and polar Tyr240 and Ser270 groups with its C-3 hydroxyl. LCA has no C-7 alkyl substituent and therefore is not attracted so strongly towards the hydrophobic pocket cleft and prefers a position where hydrogen bonds are formed with both Tyr240 and Ser270.

Finally, deeper insight into the interactions between GPBAR1 and 7-ELCA was obtained in luciferase reporter gene experiments with mutated GPBAR1 variants (Figure 9E). Amino acids Ser270, Glu169, and Tyr89, identified previously as potentially important for the interaction of ligands with GPBAR1 in various receptor models (D'Amore et al., 2014; Gertzen et al., 2015; Macchiarulo et al., 2013), were replaced with glycine, a neutral amino acid incapable to form hydrophobic or hydrophilic interactions. The mutation of Ser270 blocked the capacity of LCA to activate CRE-luc reporter construct. However, the activity of 7-ELCA was partially preserved in the same experiment. This might be explained by the hydrogen bond between Ser270 and 3-hydroxyl group of LCA, which is not present in the case of 7-ELCA. These data suggest that Ser270 may be important for the activation of GPBAR1 (Figure 9E). On the other hand, Glu169 is not crucial for GPBAR1 activation by LCA, but rather it helps to stabilize ligands in the LBD. The mutation of the residue Tyr89 did not affect GPBAR1 activation, which is consistent with our previous data (Stefela et al., 2020).

## Discussion

Modification of the BA scaffold generated several hit compounds with pharmacological activities, ranging from a selective modulation on FXR or GPBAR1 to dual modulation or even mild GPBAR1 antagonism. In this study, we introduced 7-ELCA ((E)-3α-hydroxy-7-ethylidene-5β-cholan-24-oic acid) as the first steroid compound endowed with unique and potent mixed FXR antagonistic and GPBAR1 agonistic activities. We suppose that this compound could represent prominent progress in the development of steroidal dual modulators targeting intestinal endocrine cells in the therapy of diabetes type II or other metabolic diseases.

The farnesoid X receptor regulates bile acid, lipid, and glucose metabolism (Han, 2018). Numerous FXR ligands based on steroidal or non-steroidal structures have been developed. For instance, obeticholic acid (OCA), a potent steroidal FXR agonist, is used in the therapy of ursodeoxycholic acid (UDCA)-resistant primary biliary cholangitis (PBC) and it is additionally investigated for the treatment of other liver diseases such as non-alcoholic steatohepatitis (NASH) (Ðanić et al., 2018; Ratziu et al., 2019). Despite promising results emerging from experimental models or clinical trials, significant side effects appeared during the therapy such as altered cholesterol levels, exacerbation of liver injury or cholestasis implying the potential use of FXR antagonists in the treatment of these disorders (Stedman et al., 2006; Lamers et al., 2014).

Indeed, FXR antagonistic activity has been already described for different natural compounds used as lipid lowering agents in traditional medicine including Z-GUG (Cui et al., 2003) or acanthoic acid (Han et al., 2019). In particular, the inhibition of intestinal FXR signaling appears to represent a novel strategy for the treatment of metabolic disorders. A study with a selective intestinal FXR inhibitor, Gly-MCA, demonstrated a reduction of triglyceride accumulation in the liver, decreased blood glucose levels and increased insulin sensitivity in the murine model of obesity (Gonzalez et al., 2016). The therapeutic potential of gut-specific FXR antagonists is also supported by more recent findings that metformin, a drug of choice for the treatment of type II diabetes, can antagonize FXR signaling in the intestine (Sun et al., 2018). Another study showed that capsaicin improved glucose tolerance by suppressing enterohepatic FXR signaling (Hui et al., 2019). In addition, improved glucose homeostasis and insulin resistance have been reported in FXR-deficient, but not in liver-specific FXR deficient, obese mice as well as after application of the FXR antagonist HS218 in a mouse model of type 2 diabetes (Prawitt et al., 2011; Xu et al., 2018).

Here, we introduced 7β-alkyl substituted derivatives of chenodeoxycholic acid as FXR antagonists. The modification of CDCA by 7-alkylation drown the attention before the discovery of FXR with the aim to protect the CDCA scaffold against bacterial 7-dehydroxylation occurring naturally in the intestine. Authors declared appropriate absorption and conjugation with better metabolic stability of 7-ethyl and 7-propyl CDCA derivatives. In addition, they observed reduced absorption of cholesterol from the intestinal lumen as well as lowered serum and liver cholesterol levels (Une et al., 1990; Kim et al., 2000). Fujino *et al.* then first described the importance of the 7α-hydroxyl group in FXR activation and they found that substitution of an alkyl group to the position 7β led to decreased FXR activation. In contrast to our results, they did not observe any antagonistic behavior on CDCA-induced SRC-1/FXR interaction (Fujino et al., 2004).

On the other hand, the alkylation of the BA scaffold at the position C-7 has been shown to increase GPBAR1 activation (Iguchi et al., 2011; Nakhi et al., 2019). By performing the molecular docking to GPBAR1, we observed that the alkylation at the position C-7 results in the formation of hydrophobic interactions with Phe161, Leu166, Val170, Leu244, Ser247, and Val248 amino acid residues of the LBP. We propose that the hydrophobic interactions might help to stabilize ligands in the LBP which is reflected in the reduction of the docking score of these ligands when compared to LCA. This indicates an increased affinity of the ligands toward GPBAR1.

The GPBAR1 activation has been shown to downregulate inflammation (Kawamata et al., 2003; Perino et al., 2014), decrease LDL cholesterol particles uptake (Pols et al., 2011) and attenuate weight gain (Glicksman et al., 2010) and lipid accumulation (Thomas et al., 2009). Importantly, stimulation of GPBAR1 in endocrine L cells induces the release of incretin glucagon-like peptide-1 (GLP-1), which increases insulin secretion in the pancreas (Brighton et al., 2015). This can result in increased glucose tolerance as was observed after the treatment with an endogenous GPBAR1 ligand, cholic acid-7-sulfate (CA7S) in insulin-resistant mice (Chaudhari et al., 2021). Similarly to CA7S, 7-ELCA increases the secretion of GLP-1 from intestinal cells and upregulates the expression of GPBAR1 mRNA. This suggests a dual mechanism by which both compounds target GPBAR1 signaling – direct stimulation and indirect upregulation of the receptor, which can be activated more easily by endogenous ligands such as LCA. In addition, FXR inhibition in the enteroendocrine L cells has been recently proposed to increase GLP-1 secretion (Niss et al., 2020). Previous studies have shown that FXR activation with FXR agonist GW4064 repressed transcription of GLP-1 in intestinal L cells via cAMP-CREB signaling pathway (Trabelsi et al., 2015; Li et al., 2018).

In contrast, other studies have demonstrated controversial results showing that a gut-specific FXR agonist, fexaramine, stimulates TGR5 expression and increases GLP-1 secretion in intestinal L cells and sensitivity to insulin (Fang et al., 2015; Pathak et al., 2017) via microbiome changes leading to bile acid composition alteration, resulting in enhanced TGR5 signaling *in vivo* (Pathak et al., 2018).

Despite the controversy on the role of FXR in GLP-1 secretion regulation, we can suppose that both the agonistic GPBAR1 as well as FXR antagonistic activities of 7-ELCA may synergize in GLP-1 release and contribute to the glucoregulatory mechanism of 7-ELCA. The merit needs further investigation in animal experiments to evaluate potential therapeutic activity *in vivo*.

The flaw of the treatment with GPBAR1 agonists is the occurrence of side effects, such as gallbladder filling and itching, resulting from the systemic activation of the receptor. Therefore, several strategies have been published to synthesize low-absorbed nonsteroidal GPBAR1 agonists, referred to also as gut-restricted or topical intestinal GPBAR1 agonists via modifying the parent structure with polar functional groups. For example, the identification of 4-phenoxynicotinamide derivatives led to the discovery of low-absorbed non-steroidal GPBAR1 agonists. The modification of other nonsteroidal GPBAR1 derivatives with a quarternary ammonium function or a terminal amine, with sulfonate, D-glucamine derivatives, dimerization of the core structure using a PEG-linker or conjugation of two active substances have been also reported (Duan et al., 2015; Cao et al., 2016; Ma et al., 2016; Lasalle et al., 2017; Zhang et al., 2017; Chen et al., 2018).

To conclude, we introduced 7-ELCA which is, to the best of our knowledge, the first reported BA derivative that can antagonize FXR and efficiently activate GPBAR1 at the same time. With the increasing frequency of metabolic disorders in the western population, dual FXR antagonistic/GPBAR1 agonistic potency represents an interesting synergistic pharmacological intervention and therapeutic application to this issue. Therefore, 7-ELCA warrants further structural modifications and extended studies on experimental animal models.

## Data Availability

The original contributions presented in the study are publicly available. This data can be found here: https://zenodo.org/record/3898392#.YNHKQGhKiUk.

## Abbreviations

CAR, Constitutive Androstane Receptor (NR1I3); CITCO, 6-(4-chlorophenyl) imidazo[2,1-b]thiazole-5-carbaldehyde O-(3,4-dichlorobenzyl) oxime; LBD, ligand-binding domain; LXR, liver X receptor; PPAR, peroxisome proliferator-activated receptor; PXR, Pregnane X Receptor; RT-qPCR, Reverse transcription quantitative (real-time) polymerase chain reaction; TR-FRET, Time Resolved – Fluorescence Resonance Energy Transfer.
