# Supplementary material for: (E)-7-Ethylidene-lithocholic Acid (7-ELCA) Is a Potent Dual Farnesoid X Receptor (FXR) Antagonist and GPBAR1 Agonist Inhibiting FXR-Induced Gene Expression in Hepatocytes and Stimulating Glucagon-like Peptide-1 Secretion From Enteroendocrine Cells
Source: Front Pharmacol. 2021 Aug 13;12:713149. doi: 10.3389/fphar.2021.713149 (PMC8414367; doi:10.3389/fphar.2021.713149)
Supplement: Supplementary file 1 [file DataSheet1.docx]

#

Supplementary Material

**(E)-7-ethylidene-lithocholic acid (7-ELCA) is a potent dual farnesoid X receptor (FXR) antagonist and GPBAR1 agonist inhibiting FXR-induced gene expression in hepatocytes and stimulating glucagon-like peptide-1 secretion from enteroendocrine cells**

***Alzbeta Stefela et al.***

Table of Contents

[Effects of compounds 2a-2k on cellular viability and their FXR antagonistic activity 1](#_Toc58531044)

[Molecular docking to GPBAR1 2](#_Toc58531045)

[Molecular modeling and molecular dynamics simulation to FXR 2](#_Toc58531046)

[^1^H and ^13^C NMR spectra 10](#_Toc58531047)

[Purity of final compounds – traces for analytical HPLC Method A 22](#_Toc58531048)

[LCMS – traces for analytical HPLC method B 26](#_Toc58531049)

[LRMS 37](#_Toc58531050)

[ROESY spectrum of compounds 2a, 2d and 2g 43](#_Toc58531051)

[The crystal data of compound 2h - data collection 44](#_Toc58531052)

[Acknowledgments 45](#_Toc58531053)

[References 45](#_Toc58531054)

#

# Effects of compounds 2a-2k on cellular viability

The potential cytotoxicity of 7-alkylated chenodeoxycholic acid derivatives were assessed in different human (HepG2, HepaRG and Huh7) and murine (AML12) hepatocyte-derived cell lines. For this purpose, hepatic cells were treated at increasing concentration for 24h and analyzed by MTS viability assay.

**Table S1**. FXR antagonistic activity determined in luciferase reporter gene assays in the presence of different FXR agonists OCA, GW4064 and CDCA.

| **Compound** | | **GW4064 1 µM^#^** | | **CDCA 20 µM^#^** |  |
| --- | --- | --- | --- | --- | --- |
|  |  | **10 µM** | **40 µM** | **40 µM** |  |
| **7-ethylideneLCA** | **2a** | 82.9±2.3 | 51.1±2.9 | 67.3±0.6 | |
| **7β-methyl-CDCA** | **2b** | 85.0±7.7 | 64.9±5.0 | 112.5±1.6 | |
| **7β-ethyl-CDCA** | **2c** | 89.0±6.0 | 75.3±2.5 | 67.1±9.2 | |
| **7β-vinyl-CDCA** | **2d** | 93.9±6.6 | 75.2±4.6 | 80.2±0.4 | |
| **7β-ethynyl-CDCA** | **2e** | 80.3±3.9 | 64.8±4.9 | 114.8±0.7 | |
| **7β-propyl-CDCA** | **2f** | 85.7±11.2 | 61.7±10.8 | 96.4±0.7 | |
| **7β-allyl-CDCA** | **2g** | 93.2±0.6 | 74.4±4.4 | 107.2±0.3 | |
| **7β-isopropyl-CDCA** | **2h** | 91.4±5.3 | 63.0±8.2 | 47.6±0.3 | |
| **7β-cyclopropyl-CDCA** | **2i** | 88.4±4.2 | 60.5±5.2 | 60.0±5.8 | |
| **7β-pentenyl-CDCA** | **2j** | 93.6±2.8 | * | * | |
| **7β-nonyl-CDCA** | **2k** | 84.8±1.8 | * | * | |
| **Tβ-MCA** | | N.D. | 84.8±6.2 | 95.0±5.7 | |
| **Z-GUG** | | 45.0 | 74.9±5.9 | 72.4±9.7 | |

The half maximal inhibitory concentration (IC_50_) was calculated for each compound in experiments with obeticholic acid (OCA) in the concentration of 1 μM employing dose-response fitting.

#relative response (%) to the model FXR ligand activation (set to be 100%) after treatment with a tested compound in a concentration 10 or 40 μM; * significant effect on cellular viability; N.D. not determined; Tβ-MCA – tauro-β-muricholic acid; Z-GUG – Z-guggulsterone.

**Table S****2.** Cell viability was determined using the Cell Titer 96 Aqueous One Solution Cell Proliferation Assay (MTS assay) after treatment with test compounds for 24 h in four different hepatic cell lines. Vehicle (0.1% DMSO) and background (10% SDS; v/v, toxic control) controls of cell viability were set to be 100% and 0%, respectively.

| **Comp.** | | **HepG2** | | **HepaRG** | | **Huh7** | | **AML12** | |
| --- | --- | --- | --- | --- | --- | --- | --- | --- | --- |
|  |  | **IC_50_ (µM)** | **Viability at 10 µM** | **IC_50_ (µM)** | **Viability at 10 µM** | **IC_50_ (µM)** | **Viability at 10 µM** | **IC_50_ (µM)** | **Viability at 10 µM** |
| **7-ethylideneLCA** | **2a** | > 200 | 99.7±1.2 | > 200 | 119.4±6.0 | > 200 | 110.6±9.5 | > 200 | 107.5±4.8 |
| **7β-methylCDCA** | **2b** | > 200 | 99.7±1.2 | > 200 | 119.5±1.0 | > 200 | 105.7±5.8 | > 200 | 107.6±7.7 |
| **7β-ethylCDCA** | **2c** | > 200 | 100.1±3.9 | > 200 | 116.6±1.3 | > 200 | 113.2±4.8 | > 200 | 104.9±2.0 |
| **7β-vinylCDCA** | **2d** | 168.8±1.0 | 95.9±8.3 | > 200 | 103.9±2.4 | > 100 | 117.3±5.6 | > 200 | 105.7±3.9 |
| **7β-ethynylCDCA** | **2e** | > 200 | 96.5±4.1 | > 200 | 92.9±6.5 | > 200 | 103.4±4.4 | > 200 | 97.5±6.6 |
| **7β-propylCDCA** | **2f** | 106.7±1.1 | 94.9±11.7 | 97.3 | 89.4±2.5 | > 100 | 117.0±2.3 | 90.8 | 81.2±1.0 |
| **7β-allylCDCA** | **2g** | 162.3±2.1 | 97.7±9.1 | > 200 | 125.8±4.5 | > 100 | 93.5±10.0 | 161.1 | 108.6±3.2 |
| **7β-isopropylCDCA** | **2h** | 103.3±1.0 | 93.4±7.4 | 180.8 | 100.9±6.9 | > 100 | 97.6±7.3 | 143.2 | 94.0±3.7 |
| **7β-cyclopropylCDCA** | **2i** | 178.4±1.0 | 90.9±0.9 | > 200 | 133.3±12.2 | > 200 | 97.9±10.4 | > 200 | 107.7±3.2 |
| **7β-pentenylCDCA** | **2j** | 77.3±1.2 | 87.8±6.7 | 76.5 | 93.1±4.4 | 34.0±1.5 | 114.0±7.0 | 75.0 | 126.6±7.1 |
| **7β-nonylCDCA** | **2k** | ≈10.9 | 79.8±2.5 | 18.9 | 71.6±2.8 | 21.8 | 118.0±4.9 | 12.5 | 68.0±1.4 |
|  | **β-MCA** | > 200 | 107.2±3.8 | > 200 | 108.3±7.7 | > 200 | 111.2±7.6 | > 200 | 135.1±3.2 |

#

In addition, we examined the potential effect of 7-ethylidene-lithocholic acid (7-ELCA) on viability in murine colonic GLUTag cells. IC_50_ for 7-ELCA in the cell line remains above 200 µM.


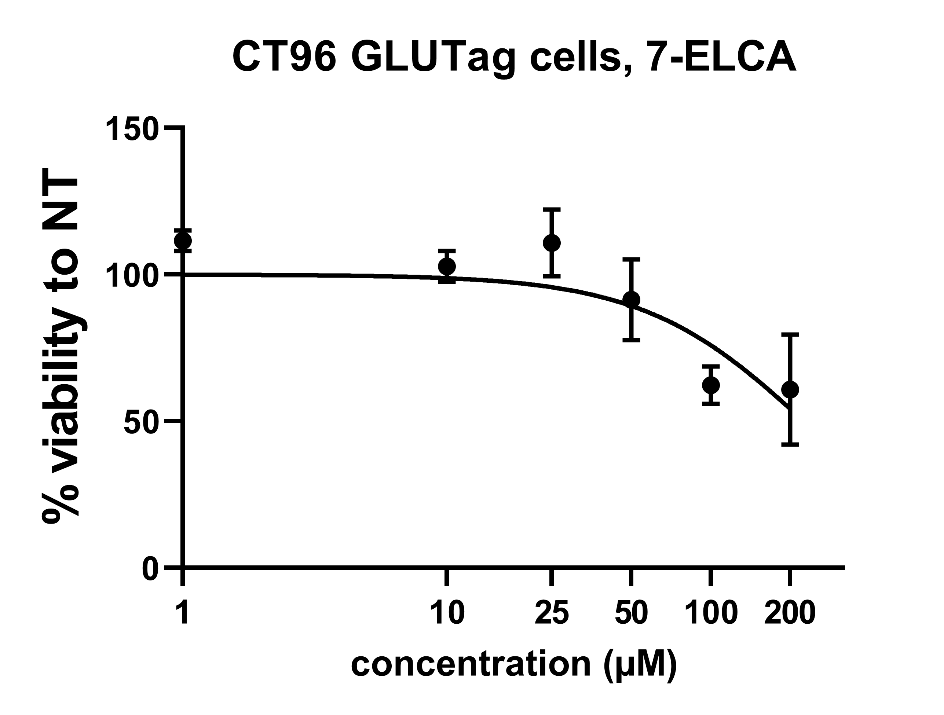


**Figure S1.** Cell viability in GluTag cells was determined using the Cell Titer 96 Aqueous One Solution Cell Proliferation Assay (Promega) after treatment with 7-ethylidene-lithocholic acid (7-ELCA) for 24 h. Vehicle (0.1% DMSO) and background (10% SDS; v/v, toxic control) controls of cell viability were set to be 100% and 0%, respectively. IC_50_ is greater than 200 μM.

# Molecular docking to GPBAR1

The ligands **2a** and **2c** (–9.9 kcal/mol, and –9.8 kcal/mol, respectively) show the lowest free binding energy. Both ligands have a short two-carbons substituent. Ligands with a 3-carbon substituent **2f**, **2g**, **2h** and **2i** as well as the compound **2e** with a rigid substituent show only a slight lowering of the binding energy in comparison with LCA (-9.2 kcal/mol).

Compound **2k**, alkylated with a nonyl on the C-7, is the only studied ligand that exhibits different behavior. Among the top calculated poses, there is one in which the ligand enters the LBD by the carboxyl group, leaving the A-ring close to the pocket entrance. The free binding energy of
–8.2 kcal/mol is significantly higher when compared with LCA. Even the pose with the A-ring down in the pocket scored only –8.1 kcal/mol. Compound **2j**, which is substituted by the pent-4-enyl group, also shows significantly higher free binding energy (–8.7 kcal/mol). We can conclude that alkyl substitution on C-7 is in general beneficial, but only to some extent. A too-long substituent can completely break ligand interactions, leading to inactivity of the ligand. The best performance showed 2-carbons substituents.


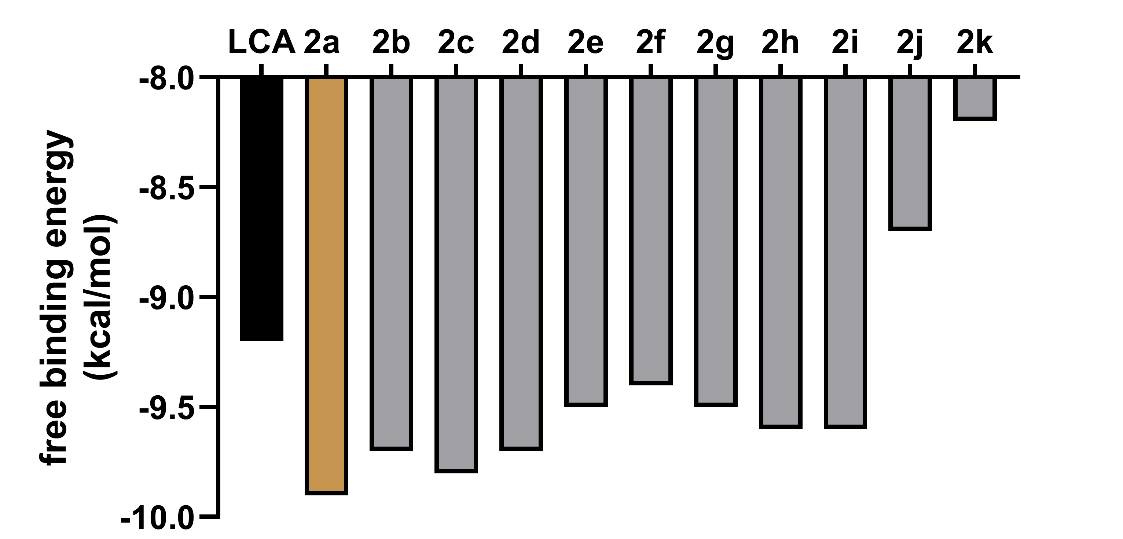


**Supplementary Figure S2**. Docking score of top scored pose of each compound on human bile acid receptors GPABR1.

#

# Molecular modeling and molecular dynamics simulation to FXR

The PDB structure of human monomer ligand binding domain of FXR co-crystallized with CDCA (PDB ID: 6HL1(Merk et al., 2019), resolution: 1.60 Å) was selected for docking and simulation based on the quality and similarity between the co-crystallized ligand and our series. The 6HL1 structure was chosen due to the high resolution, completeness, and high similarity between CDCA and our ligands series.

Other structures with different co‑crystallized ligands, such as the partial agonist GW4064 (PDB ID: 3DCT(Akwabi-Ameyaw et al., 2008), resolution: 2.5 Å) and its N‑oxide pyridine analogue, 7b (3FXV(Feng et al., 2009), resolution: 2.26 Å), the antagonist MFA-1 (PDB ID 3BEJ^1^, resolution: 1.9 Å), as well as the apostructure bound to the NCoA‑2 (6HL0, Resolution: 1.66 Å), were simulated for comparison purposes. The Small-Molecule Drug Discovery Suite (v2019.4, Schrödinger, LLC, New York, NY, 2019) was used for all calculations. Protein structures were prepared by adding hydrogen atoms and fixing missing sidechains using the Protein Preparation Wizard (PrepWiz)(Sastry et al., 2013), missing loops (Lys339 – Pro341) were generated using Prime. NCoA-2 co-activator peptide was kept in the AF-2 region, and water and sulfate/crystallization buffer molecules, such as glycerol (GOL) were removed. The ionization states of amino acids’ side-chains were stablished using Epik (Schrödinger 2019v4) with pH=7.4, specifically His447 displayed the ionization on the N_D_ atom, stablishing a hydrogen bond with the hydroxyl group from the original ligands.

Molecular docking of our series ligands and other antagonists such as MCA and GUG were performed in a grid encompassing residues around 10 Å from the centroid of the co-crystallized ligand, using the default settings of the Glide program (Glide v7.7, Maestro v2019.4) in standard precision mode, with at least five poses selected for further visual inspection(Friesner et al., 2004). Amino acid residues were considered rigid. All ligands were prepared using LigPrep (v2019.4) with standard options.

Protein complexes with the original co-crystallized ligands and with the docked ligands were submitted for molecular dynamics simulations (MD) in order to evaluate both the ligand stability and the effects of ligand-binding upon the protein (the full protocol was previously described(Kronenberger et al., 2019). MD simulations were carried out using Desmond(Dror et al., 2010), with the OPLS3e force-field(Harder et al., 2016). The simulated system encompassed the protein-ligand complexes, a predefined water model (TIP3P(Jorgensen et al., 1983)) as a solvent and counterions (Na^+^ or Cl^-^ adjusted to neutralize the overall system charge). The system was treated in a cubic box with periodic boundary conditions specifying the shape and the size of the box as 13 Å distance from the box edges to any atom of the protein. We used a time step of 1 fs, the short-range coulombic interactions were treated using a cut-off value of 9.0 Å using the short-range method, while the smooth Particle Mesh Ewald method (PME) handled long-range coulombic interactions(Darden et al., 1993). Initially, the relaxation of the system was performed using Steepest Descent and the limited-memory Broyden-Fletcher-Goldfarb-Shanno algorithms in a hybrid manner. The simulation was performed under the NPT ensemble for 5 ns, implementing the Berendsen thermostat and barostat methods. A constant temperature of 310 K was kept throughout the simulation by first using the Nose-Hoover thermostat algorithm, and then the Martyna-Tobias-Klein Barostat algorithm to maintain 1 atm of pressure After minimization and relaxation of the system, we continued with a single production step of at least 500 ns, with sampling every 1,000 ps. Trajectories, interaction data and associated movie content are available on the Zenodo repository (under the code: 10.5281/zenodo.3898392).

The representative structure was selected by inspecting changes in the Root-mean-square deviation (RMSD). Figures S2 and S3 represent the variation of the RMSD values, along with the simulation, for both template crystal structures and simulations with docking poses. Accordingly, the changes in the Root-mean-square fluctuation (RMSF), normalized by residue for the protein backbone, are displayed in Figures S4 and S5. Most of the ligand poses, with exception of 2k, were stabilized after few nanoseconds of simulation as displayed by the ligand RMSD <2 Å after the stabilization (Figure S2,3).

Interactions and distances were determined using the Simulation Event Analysis pipeline implemented in Maestro (Maestro v2019.4). The current geometric criteria for the protein-ligand hydrogen bond is a distance of 2.5 Å between the donor and acceptor atoms (D — H···A); a donor angle of ≥120° between the donor-hydrogen-acceptor atoms (D — H···A); and an acceptor angle of ≥90° between the hydrogen-acceptor-bonded atom atoms (H···A — X). Similarly, protein-water or water-ligand hydrogen bond had a distance of 2.8 Å between the donor and acceptor atoms (D—H···A); a donor angle of ≥110° between the donor-hydrogen-acceptor atoms (D—H···A); and an acceptor angle of ≥90° between the hydrogen-acceptor-bonded atom atoms (H···A—X). Non-specific hydrophobic interactions are defined by a hydrophobic sidechain within 3.6 Å of a ligand's aromatic or aliphatic carbons, and π-π interactions required two aromatic groups stacked face-to-face or face-to-edge, within 4.5 Å of distance.


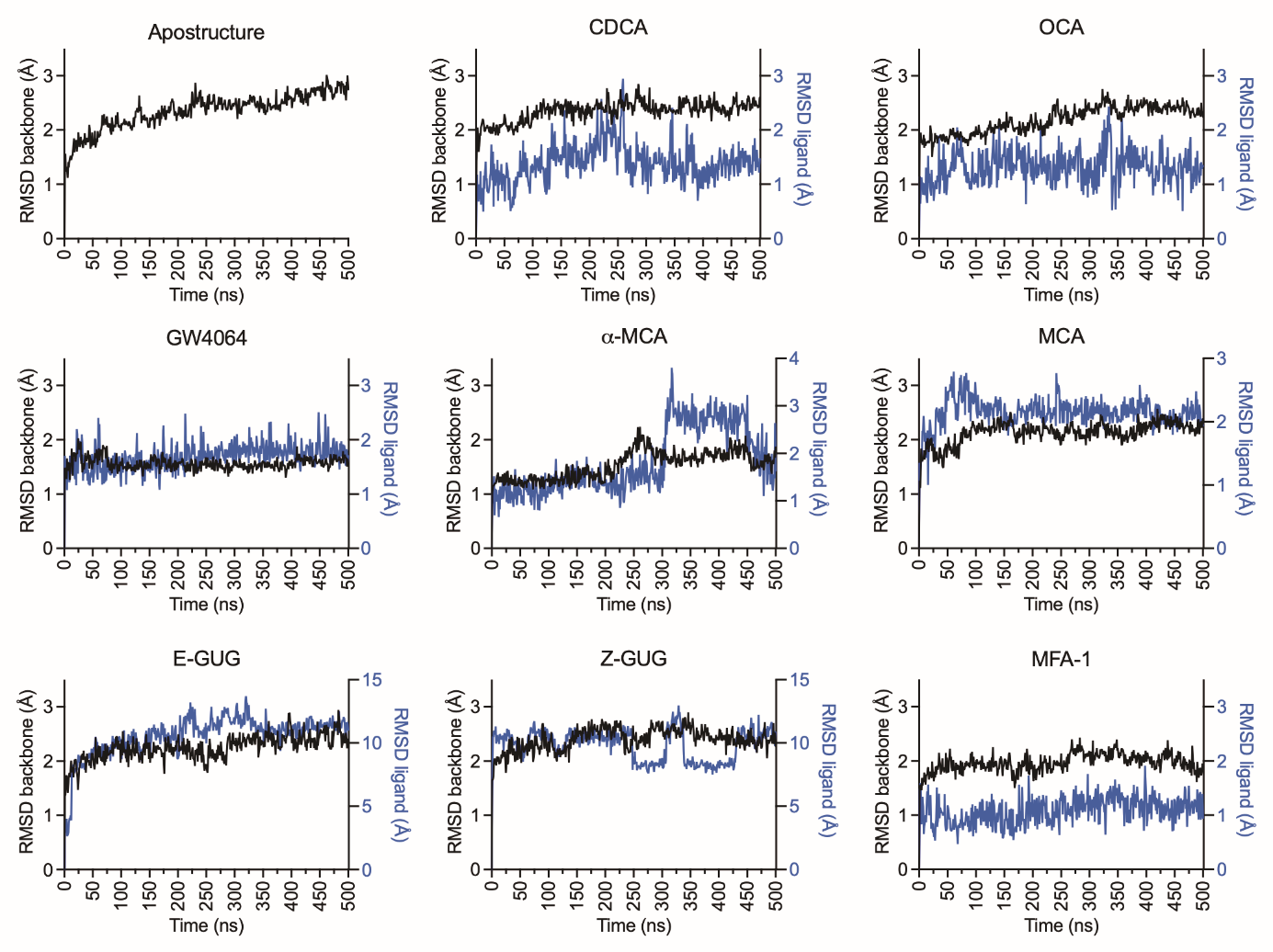


**Supplementary Figure S3.** Root mean square deviation (RSMD) values of the protein backbone (black) and ligand heavy atoms (blue) for the representative agonists and antagonists’ complex structures and no ligand simulation (apostructure), meaning only FXR+NCoA2, monitored along the individual 500 ns production phase of the MD simulations for the whole proteins.


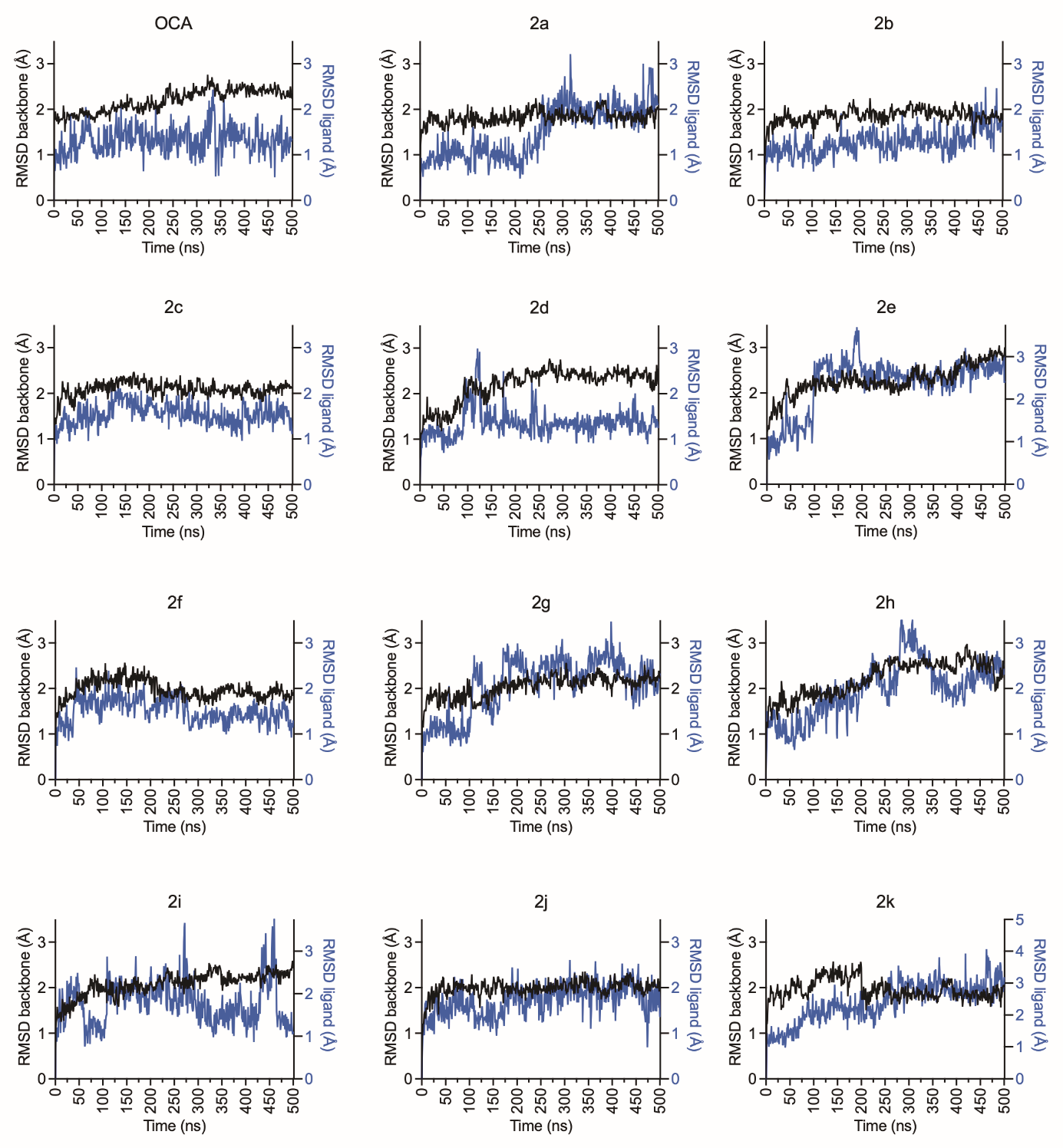


**Supplementary Figure S4.** Root mean square deviation (RSMD) values of the protein backbone (black) and ligand heavy atoms (blue) for the eleven complex structures from our FXR proposed ligands, monitored along the individual 500 ns production phase of the MD simulations for the whole proteins.


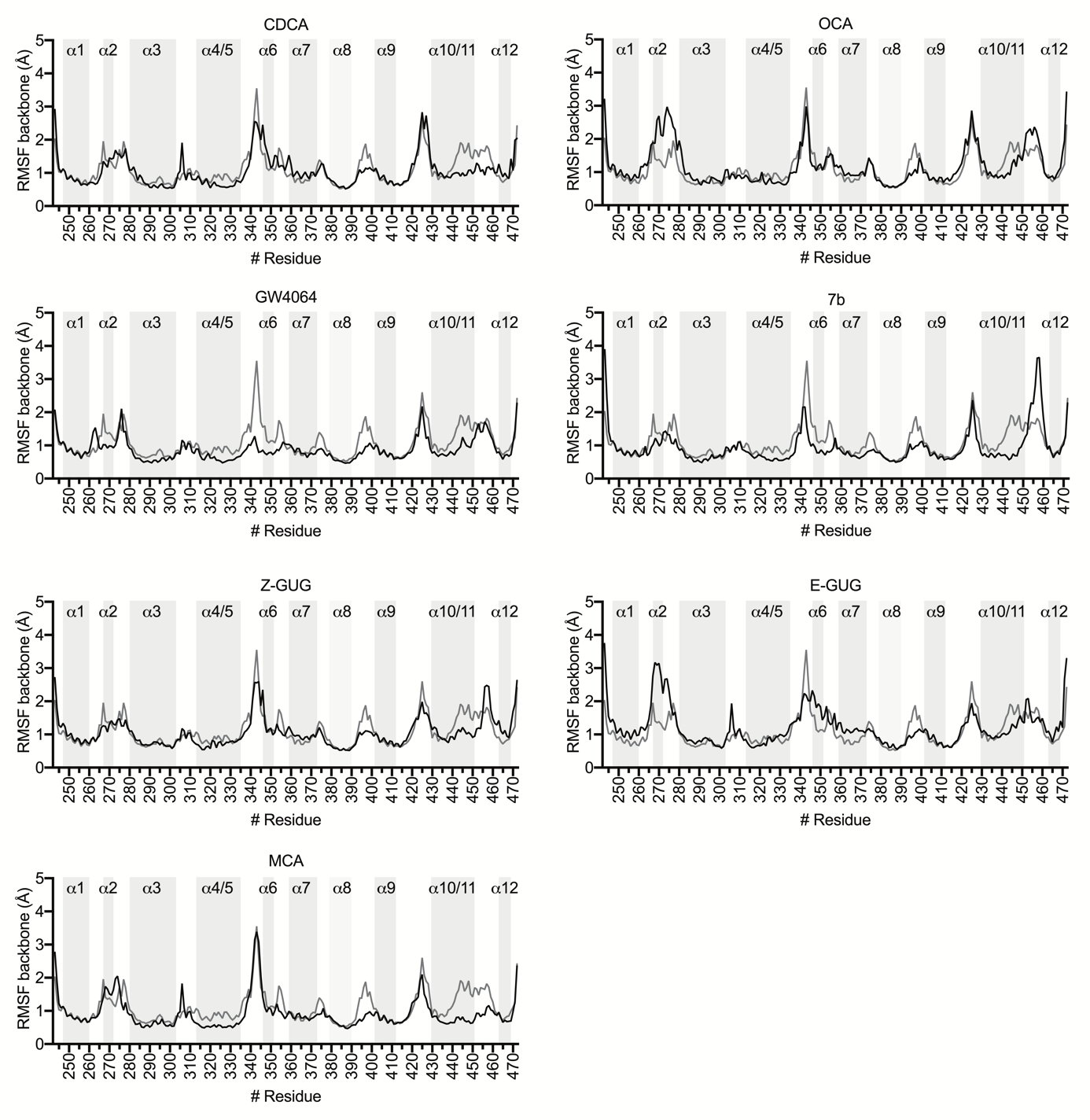


**Supplementary Figure S5.** Average residue fluctuations obtained from root mean square deviation fluctuations (RMSF) of the FXR backbone atoms for simulations with template ligands (black line), calculated in relation to the initial simulation frame, shown in comparison to the simulation of apostructure (grey line). Alpha-helices are highlighted by grey shades and numbered from α1 – α12.


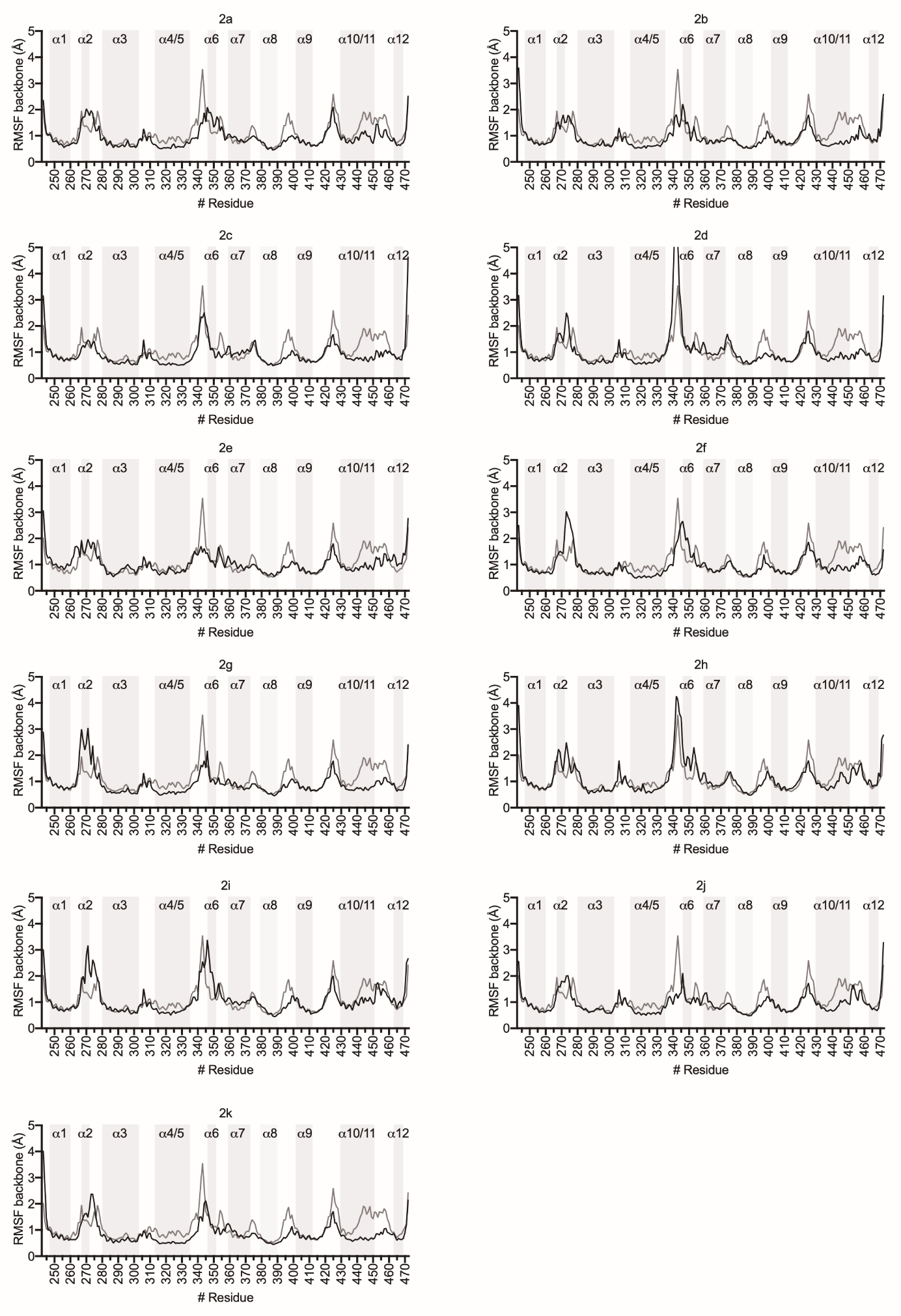


**Supplementary Figure S6.** Average residue fluctuations obtained from root mean square deviation fluctuations (RMSF) of the FXR backbone atoms for simulations with docked ligands (black line), calculated in relation to the initial simulation frame shown in comparison to the simulation of apostructure (grey line). Alpha-helices are highlighted by grey shades and numbered from α1–α12.

Most of our ligands, with the exception of **2d** and **2h**, stabilize the L:α5-α6 folding upon binding, as shown by the reduction in the RMSF values when compared to the apostructure (Figure S4,5). Interestingly, the antagonist GUG increases the RMSF values of L:α11-α12, suggesting destabilization of the AF-2 region. This destabilization was already shown for the partial agonist binding, where the electron density in the co-crystals is ambiguous^2^.

FXR agonists, such as CDCA, GW4064 and 7b, bind close to helix 4. However, for partial agonists such as the anthranilic acid derivatives^3^, the binding mode extends towards L:α11-α12. Ligands from our series also have a proposed binding mode similar to CDCA and OCA, both in their docking and as observed in the rat’s structure, with a decreased RMSF at the end of helix 11 and L:α11-α12, which suggests the stabilization of this region upon binding (Figures S4,5).


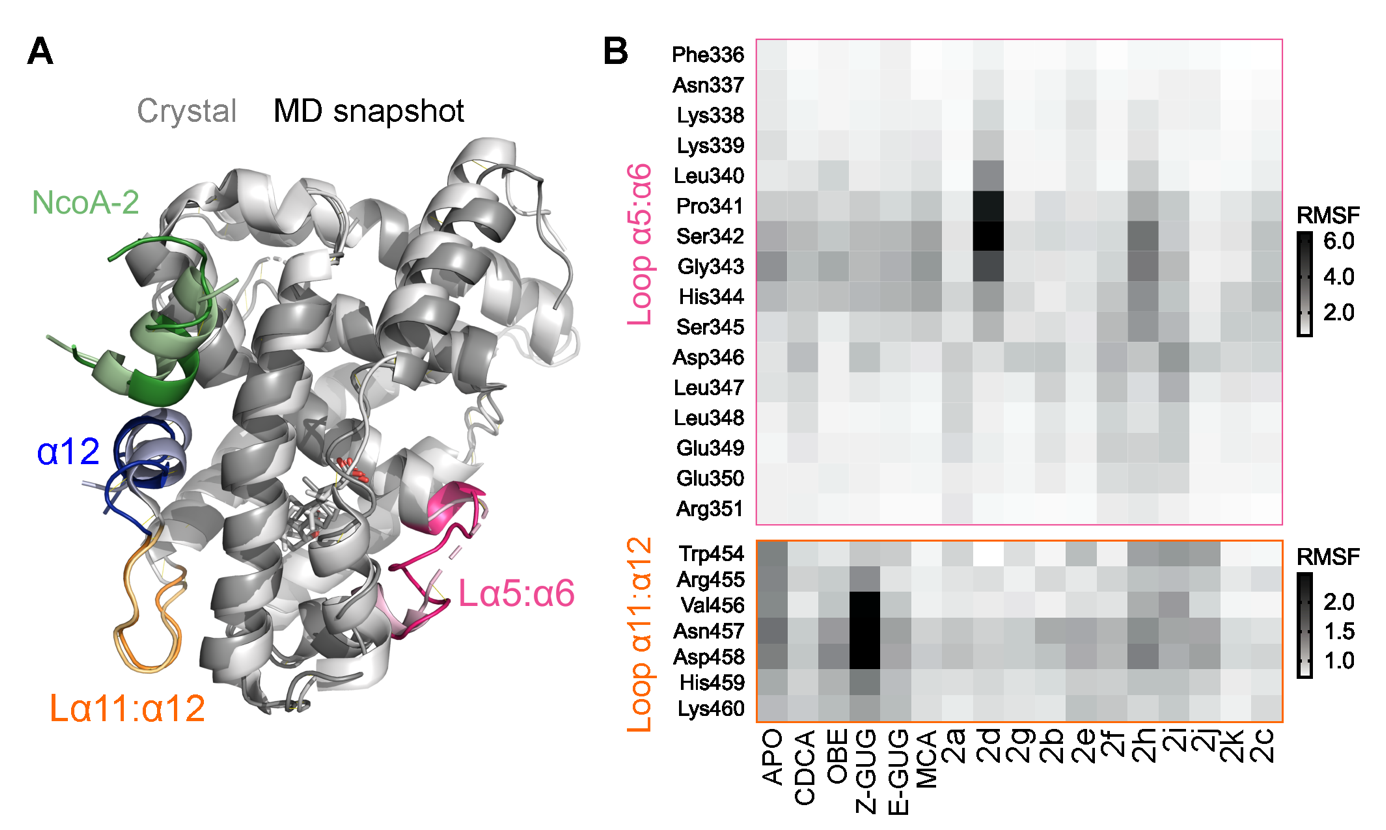


**Supplementary Figure S7.** A) localization of loops relevant for LBD’s stabilization upon ligand binding and B) variation of RMSF values in those loops in simulations with different ligands in comparison with apostructure (PDB: 6HL0).

All simulated ligands with a carboxylate moiety stablished stable interactions with the Arg264 and Arg331 with intermittent polar contacts with the His294 (Figure S7a). The partial agonists (GW4064 and 7b) displayed an additional water-mediated interaction with the Met265 main-chain atoms. Interestingly, both OCA and CDCA presented recurrent hydrogen interactions between the 7α-hydroxyl’s group and Ser332 and Tyr369, which were less prominent in our antagonists, with exception of 2d. The trajectories of ligands of the simulations for the ligands of interest are available in the associated movie material submitted in the Zenodo link.

Finally, interactions between the 3α-hydroxyl group and Tyr361/His447, although represented in the crystal structure, were not conserved in simulations. We hypothesize that the free His447 could influence the conformation of the α11 and, so the heterodimerization interface. However, the extent of this conformational change would need to be addressed by longer monomeric simulations and simulations with the heterodimer.

Additionally, simulated FXR agonists and ligands from our series remained in close distance to Trp454 (Figure S7c), while in simulations with antagonist GUG, this residue had an ambiguous behavior both by moving outwards or by keeping itself inwards, but far from any hydrophobic regions of the ligand. This outwards movement is related to the destabilization of the helix α12, and therefore to the co-activator binding.

Previous reports suggest that anthranilic acid derivatives(Merk et al., 2014) would cause a similar outward movement of Trp454, by a distance of 12 Å, induced by the tert-butyl moiety. Mutations on Trp454 are known to prevent CDCA agonistic activation^2^, but to not affect GW4064 agonism, which suggests that, despite highly relevant for FXR functionality, this motion is not essential for all pharmacological modulation profiles.


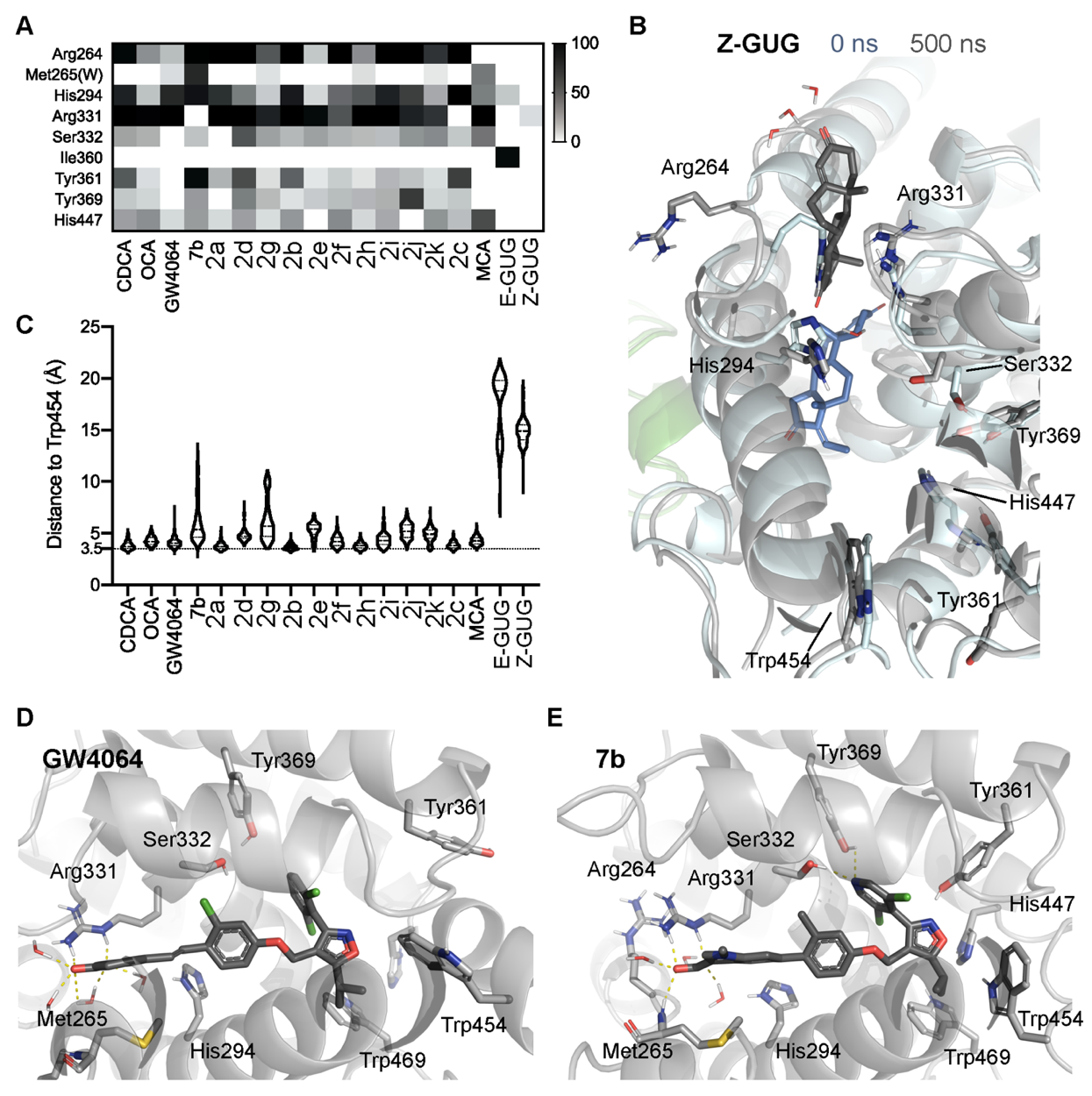


**Supplementary Figure S8.** A) The frequency of hydrogen interactions observed, with the production phase of molecular dynamics simulation. Methionine 265 interactions are mediated by water molecules and atoms of the main-chain. B) The initial docking pose and the last frame of the simulation with Z-GUG show an unstable trajectory that corroborates the lack of Suplementary hydrogen bond interactions. C) The minimum distance between hydrophobic atoms from ligands and the side-chain of Trp454, highlighting that most of the ligands remain in close interaction with this residue, in an inward conformation, with the exception of both GUG isomers. Representative snapshots from the molecular dynamics simulation with D) GW4064 and E) GW4064’s analogue, 7b. Interactions are represented by dashed lines. DHFR residues are colored according to the atom types of the interacting amino acid residues (protein’s carbon in light grey, nitrogen in blue, oxygen in red).

# ^1^H and ^13^C NMR spectra

# **Purity of final compounds – traces for analytical HPLC Method A**

Experimental details are in the full text.


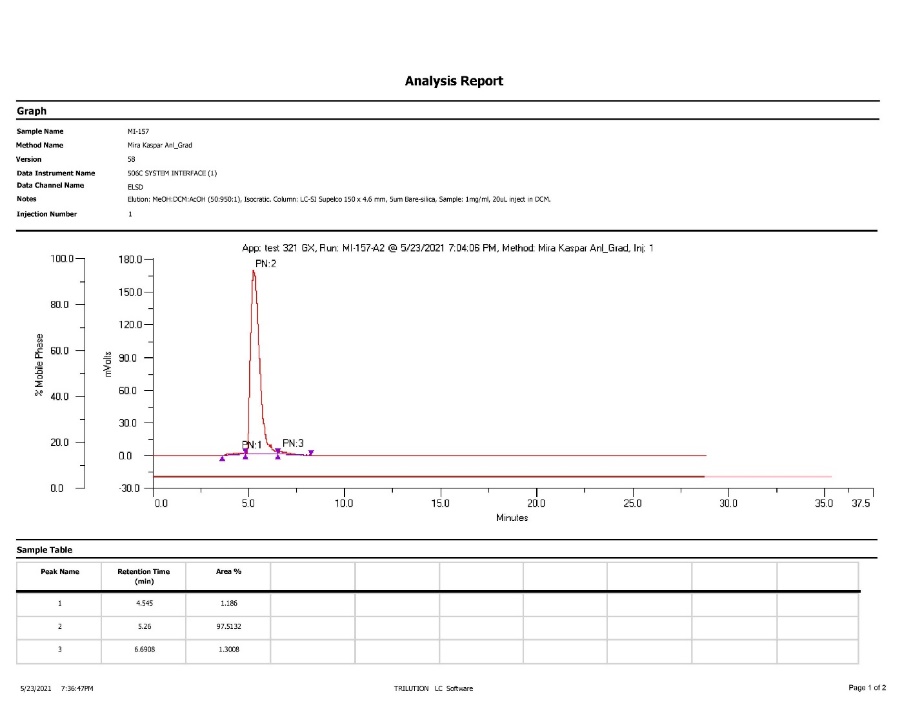


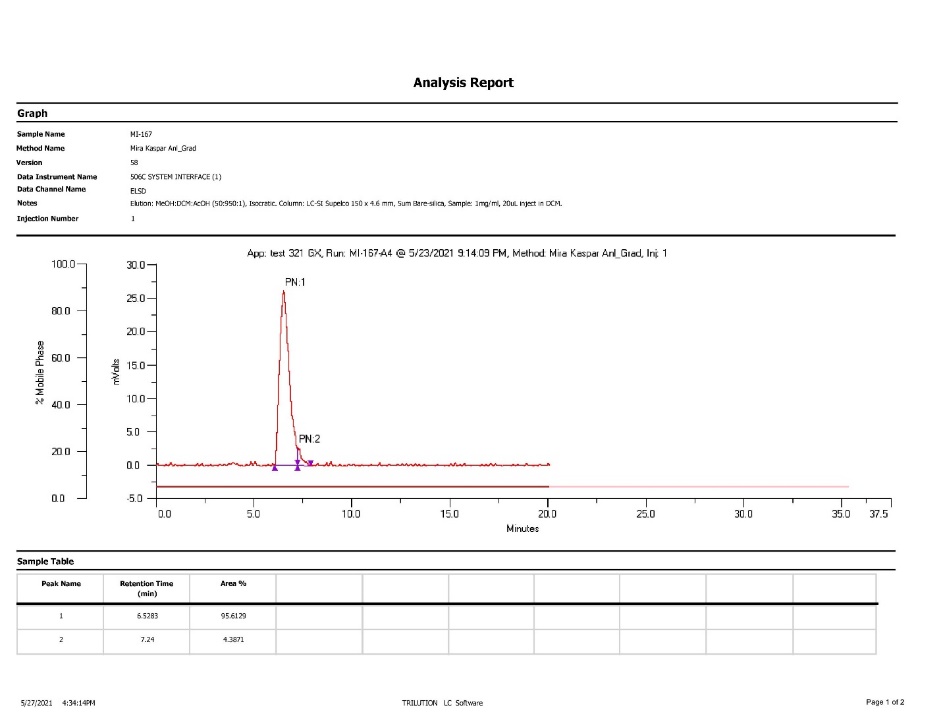


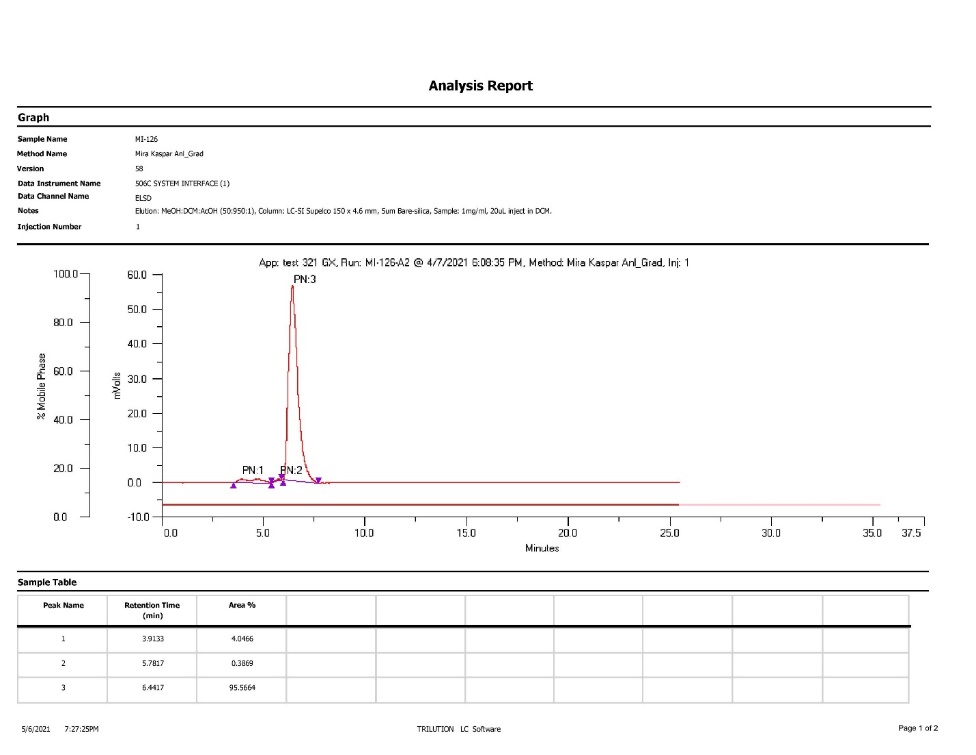


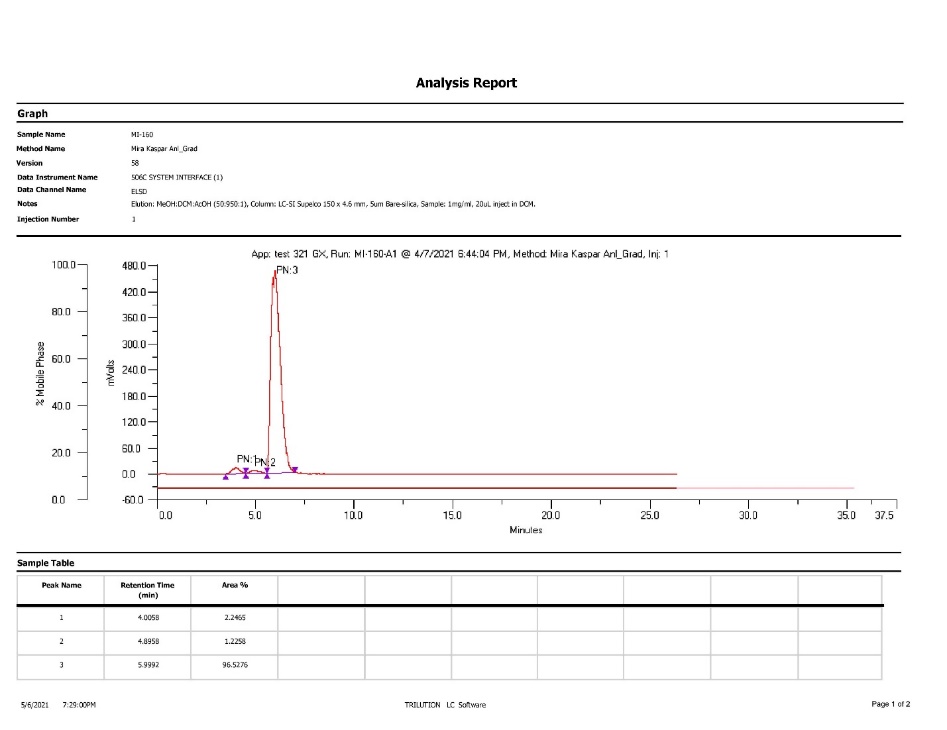


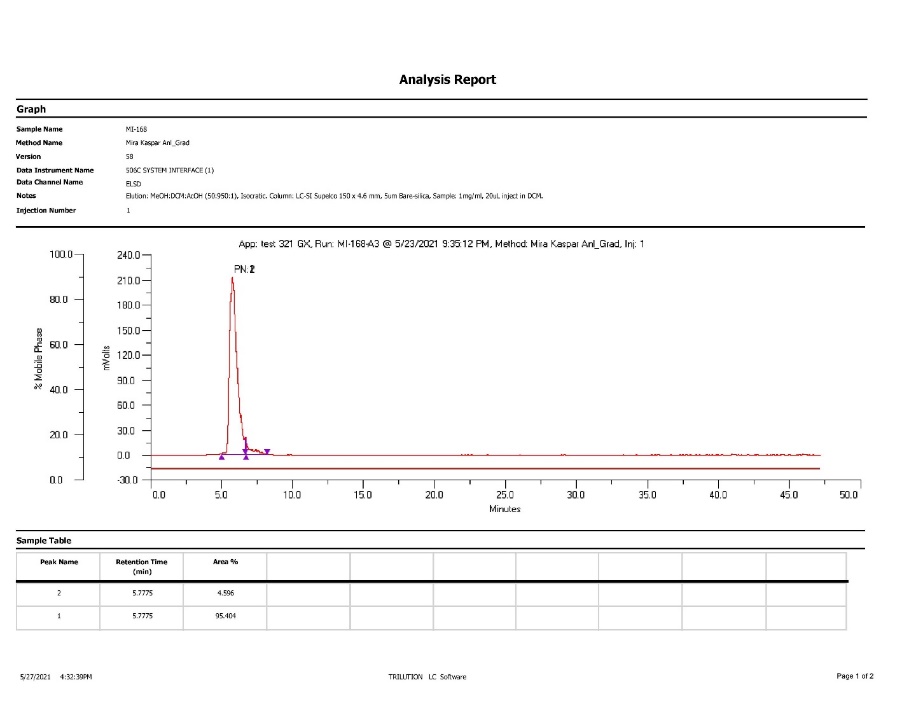


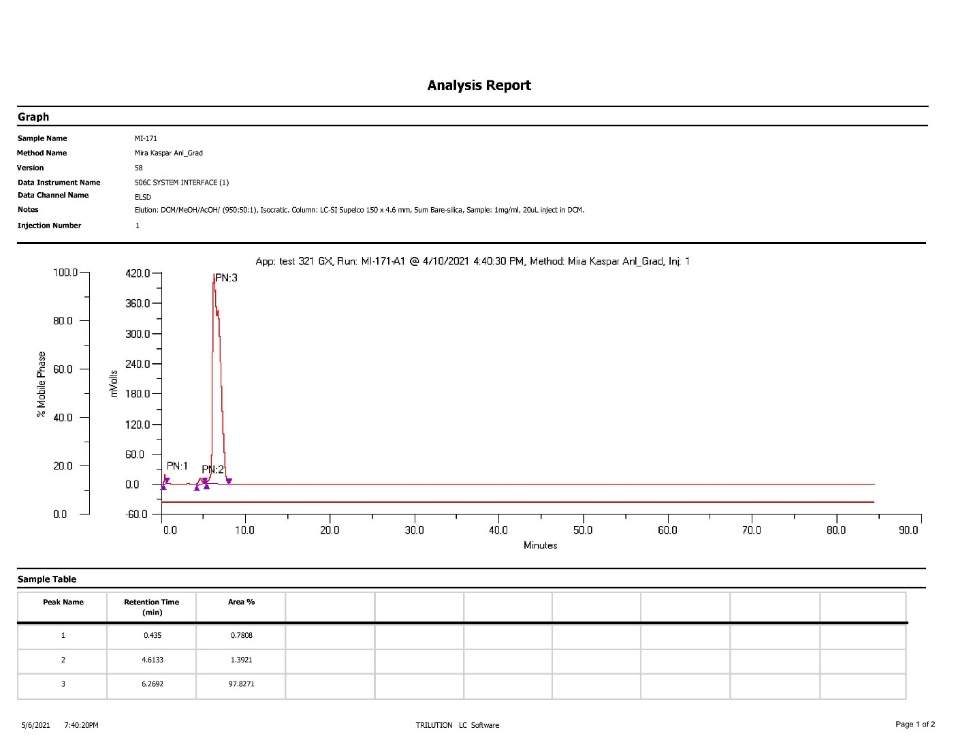


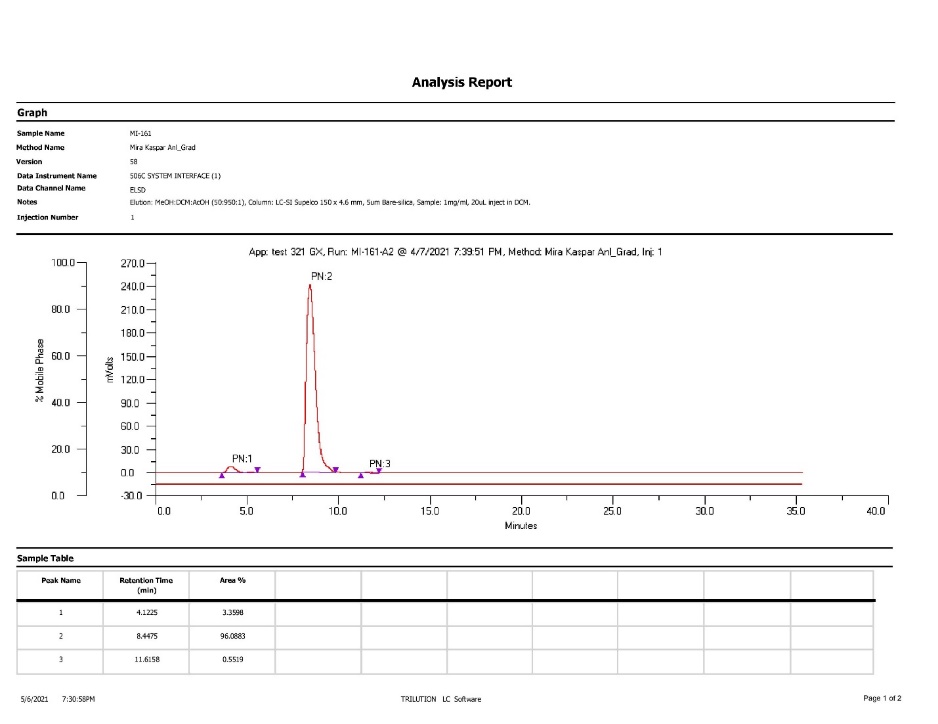


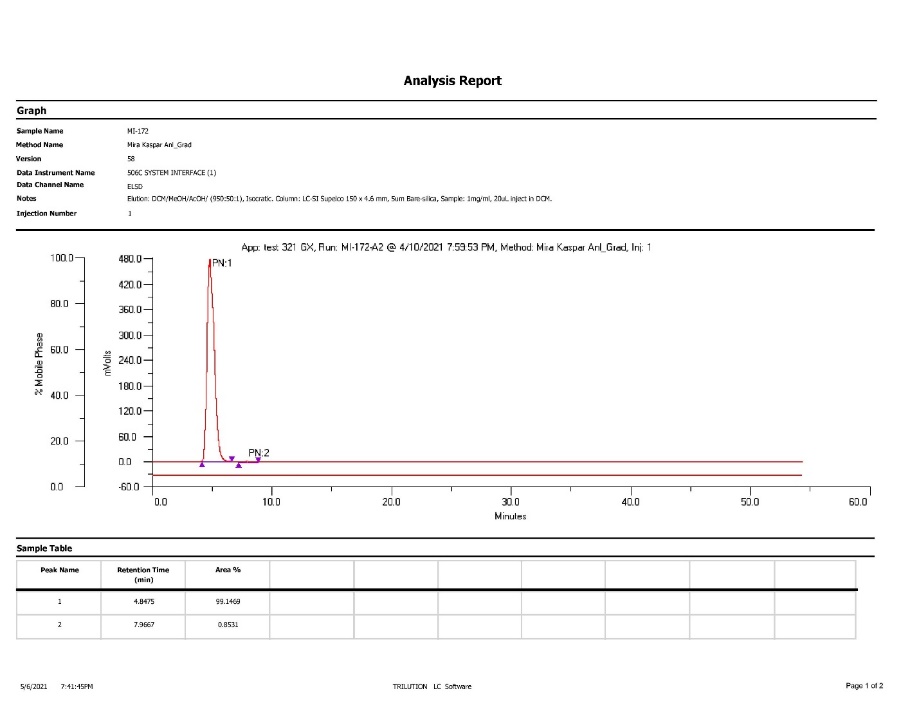


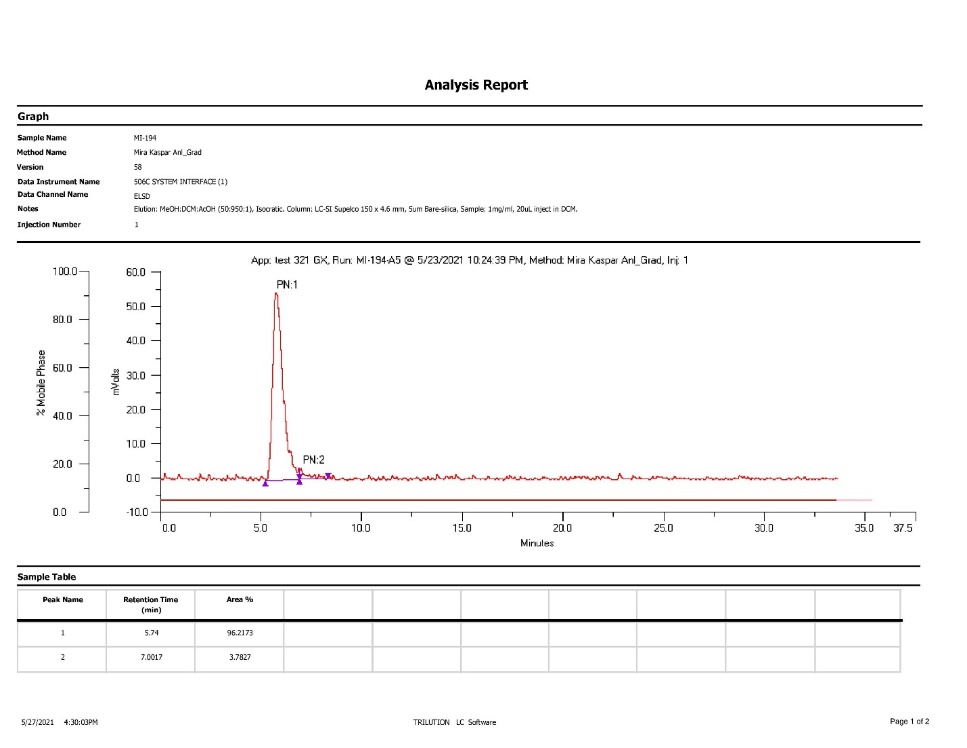


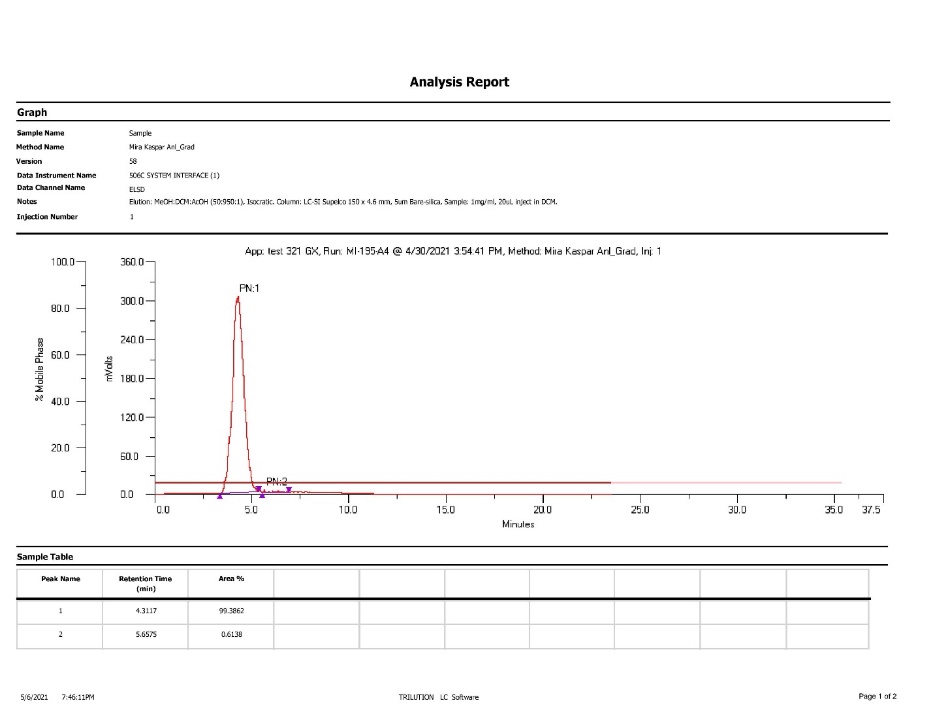


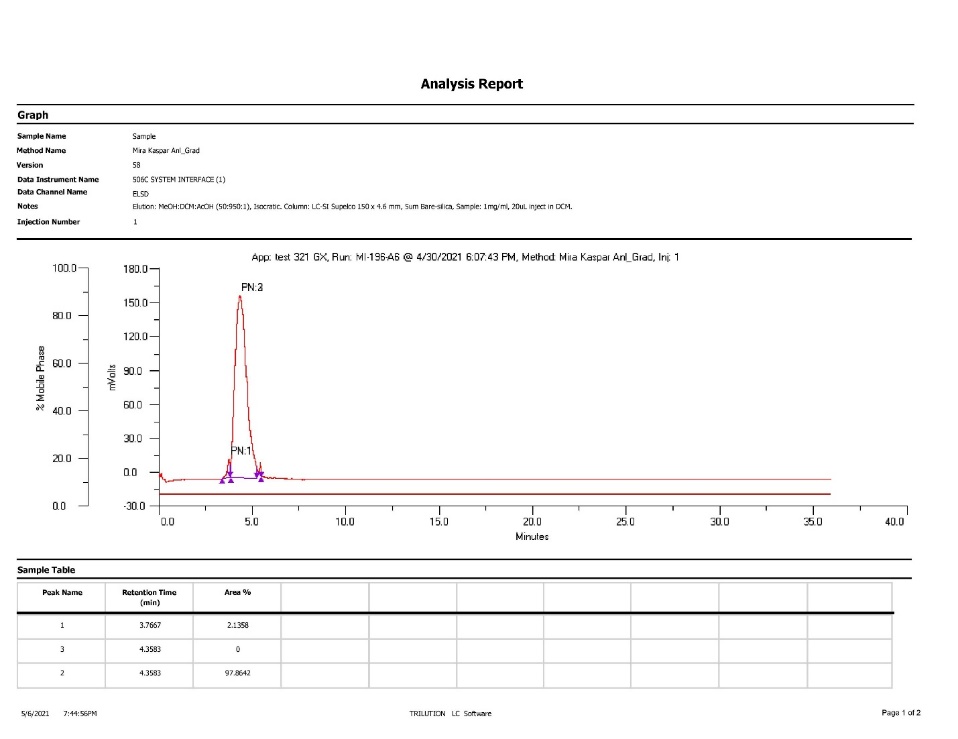


# LCMS – traces for analytical HPLC method B

Experimental details are in the full text.


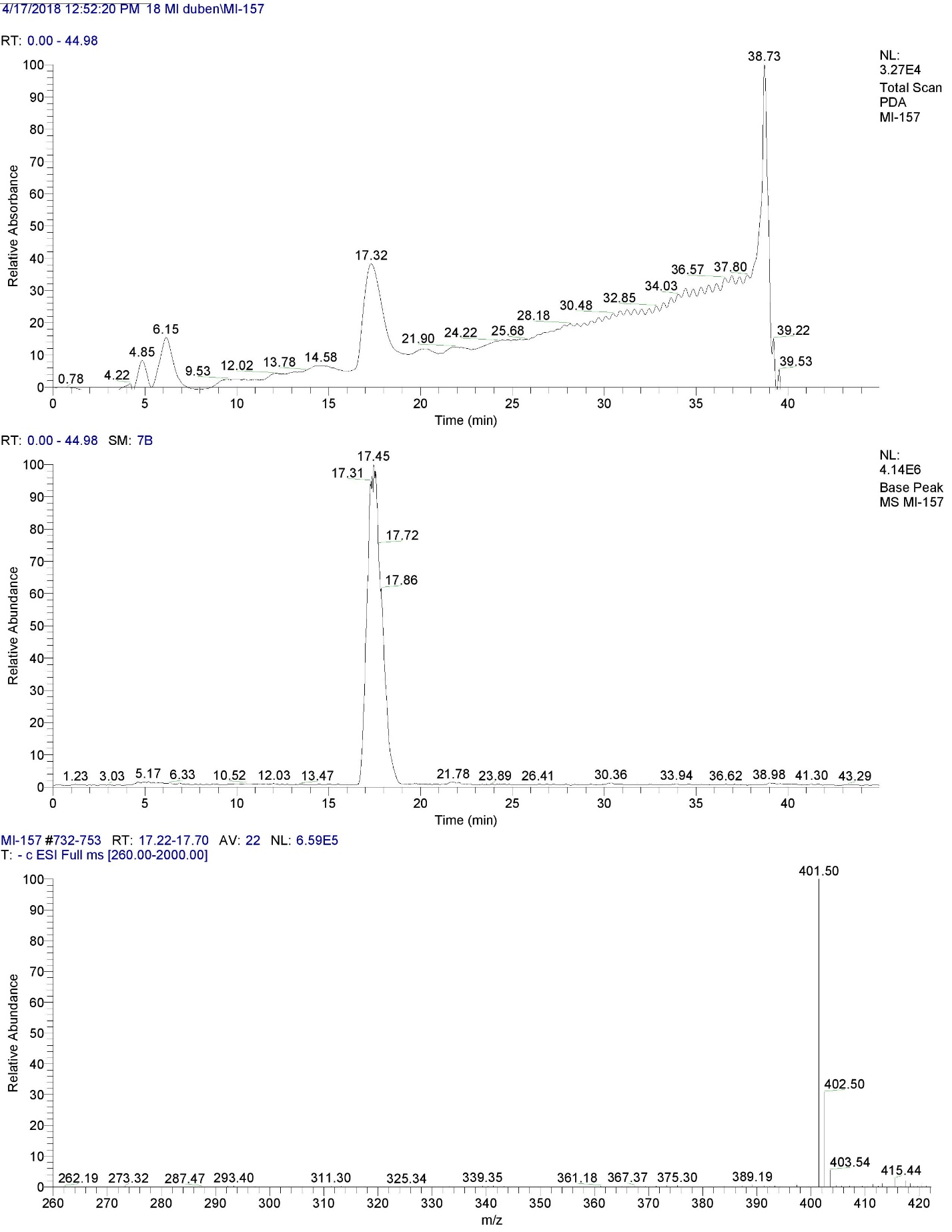


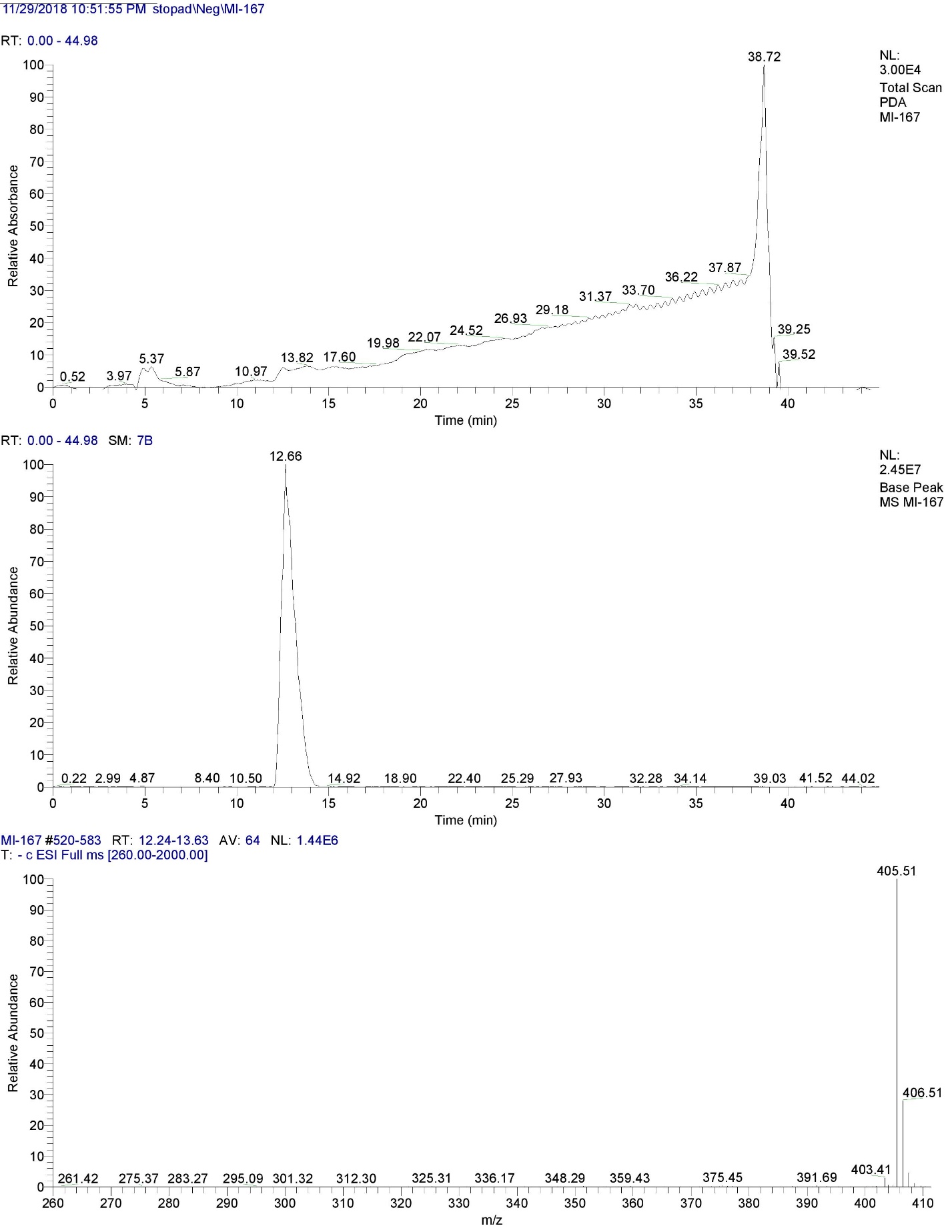


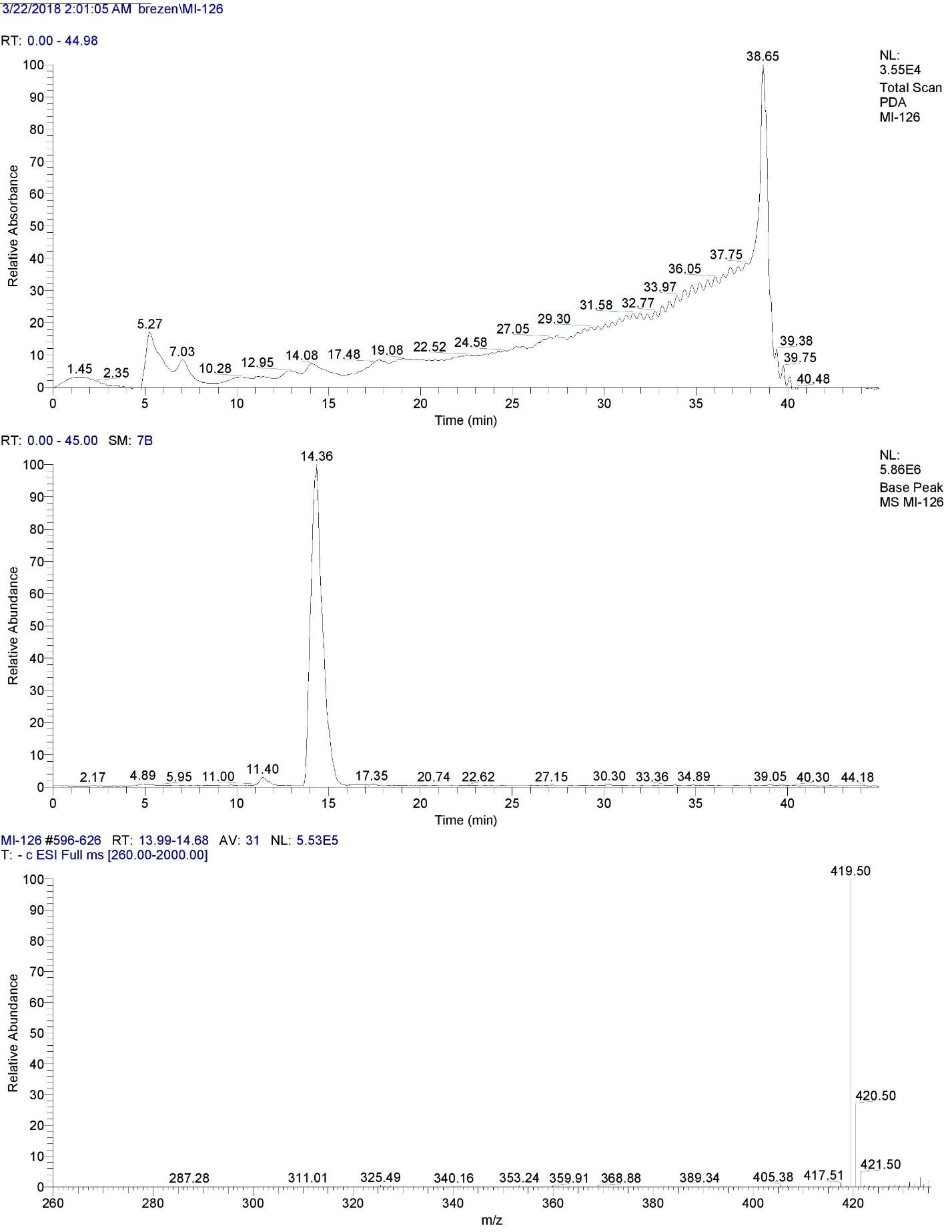


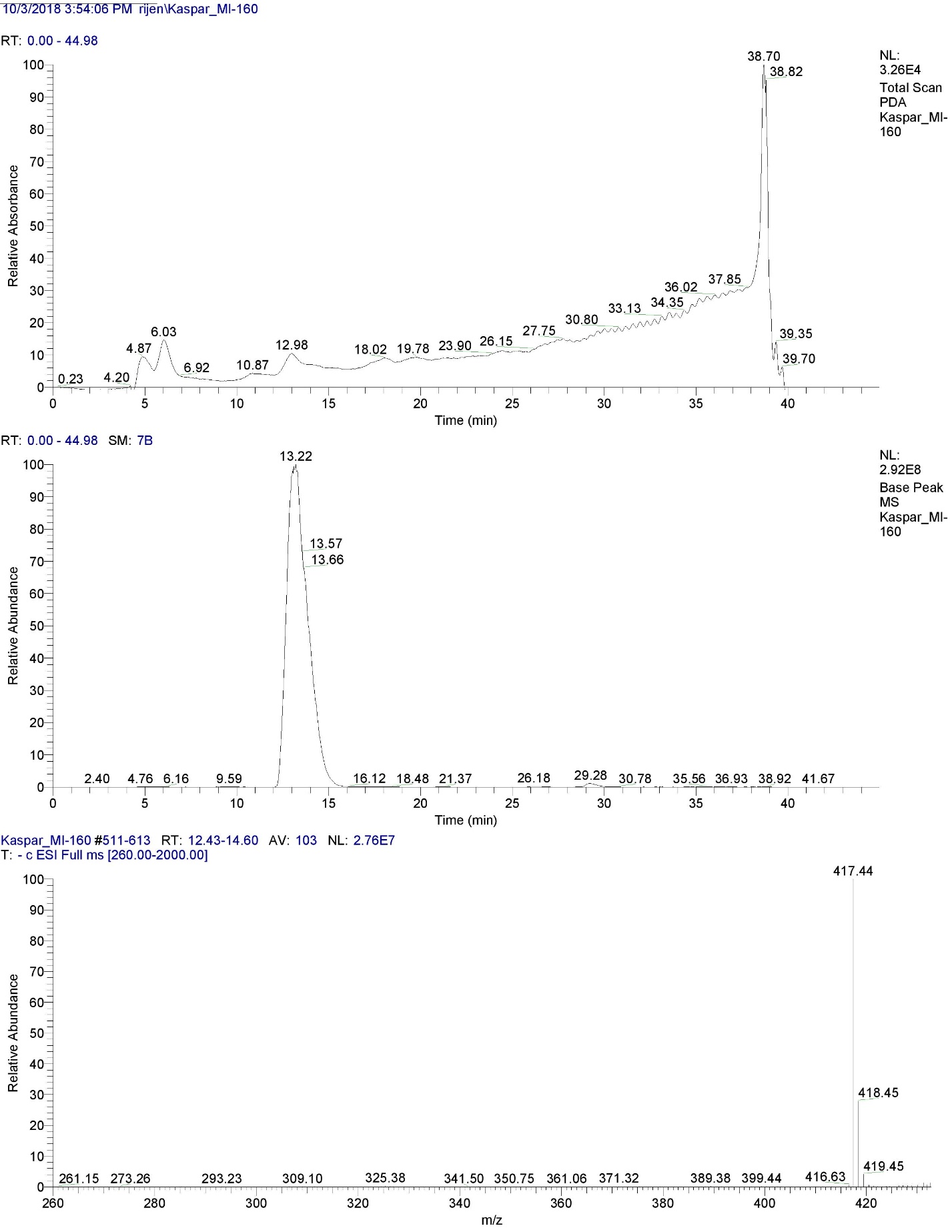


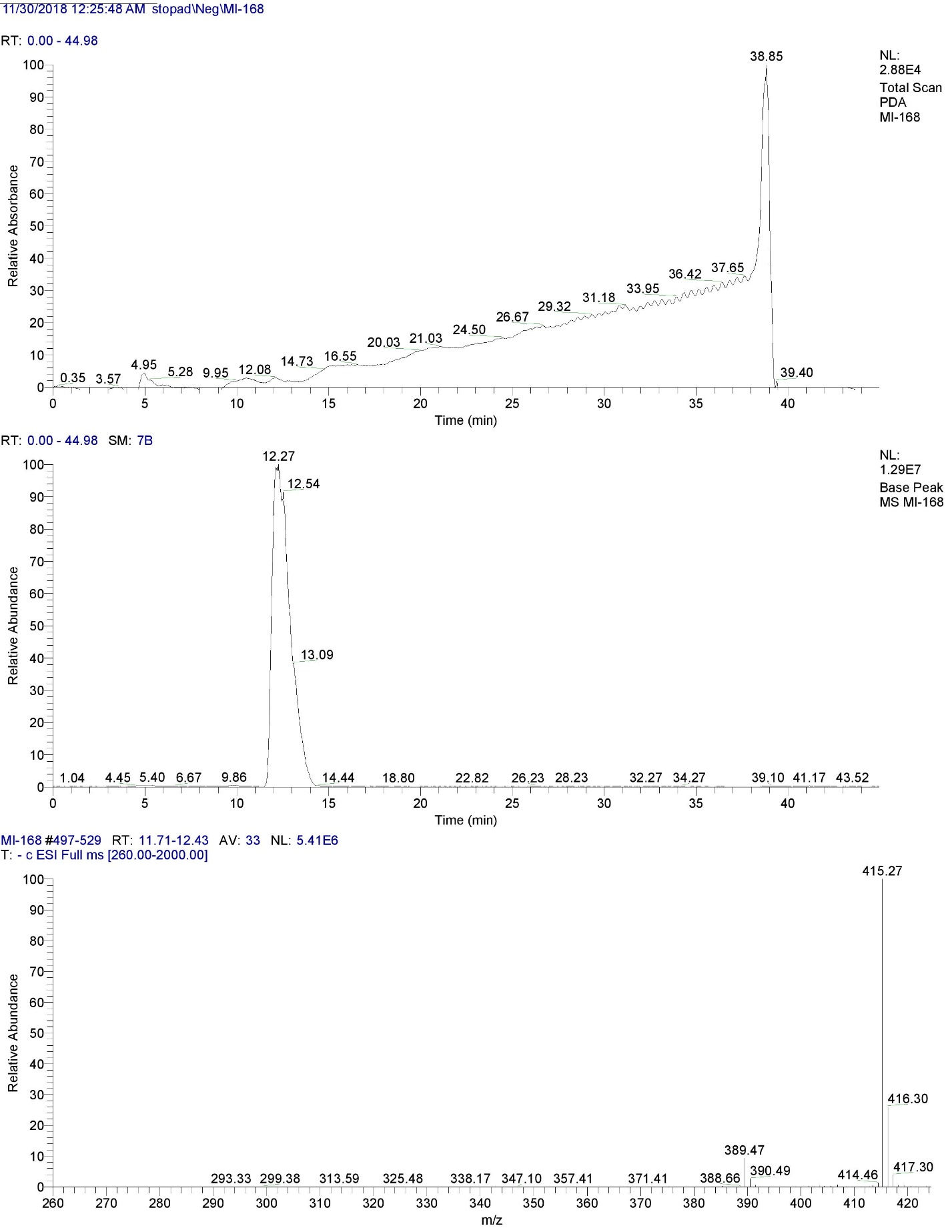


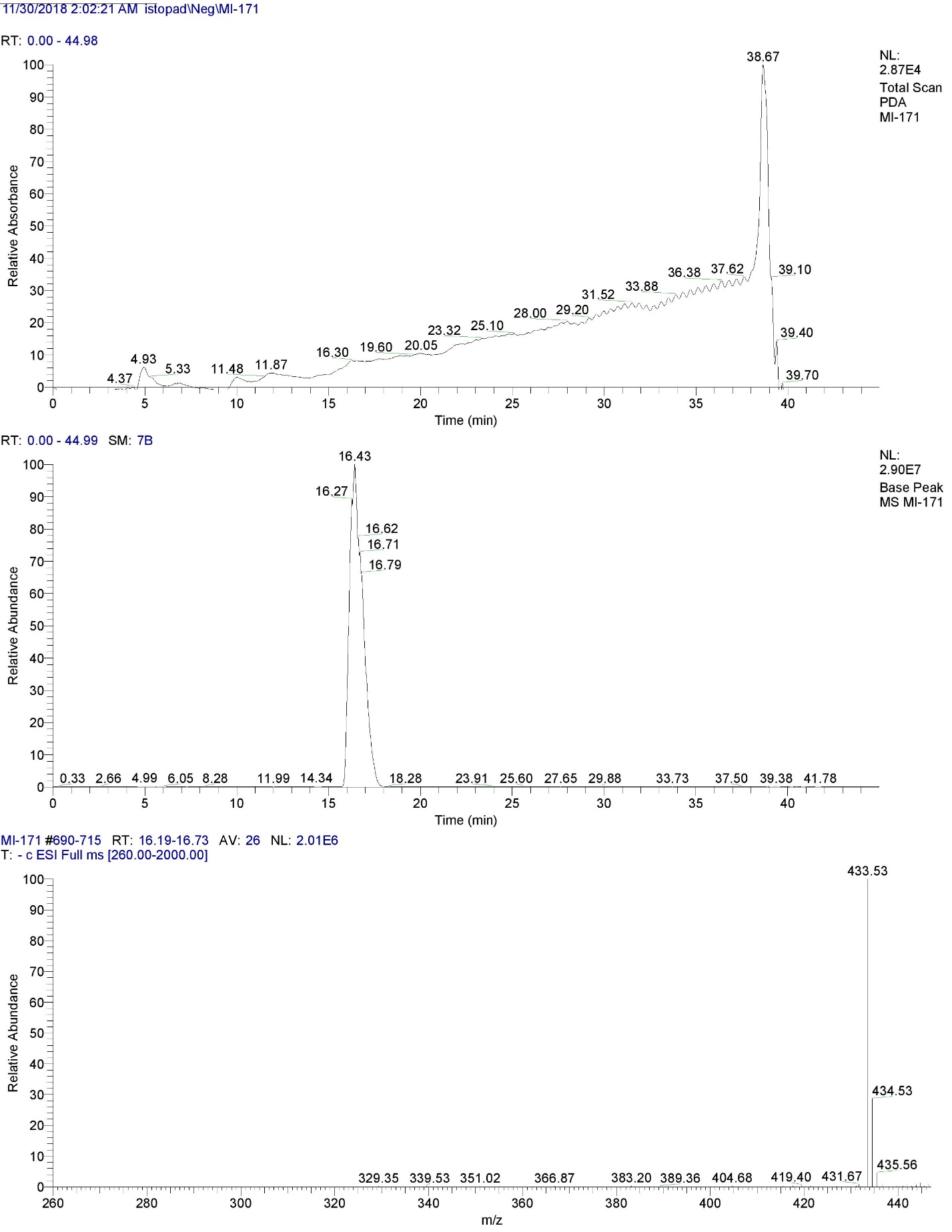


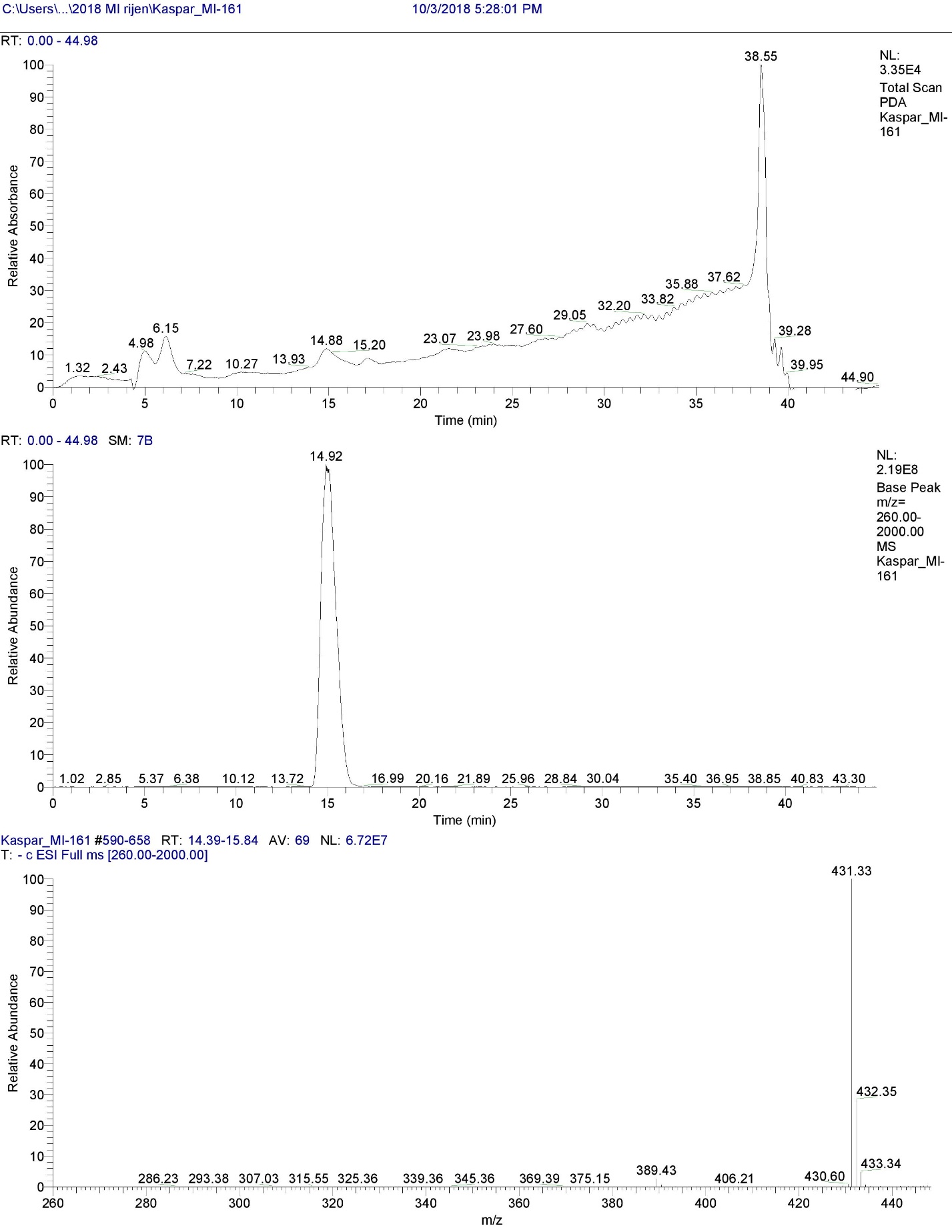


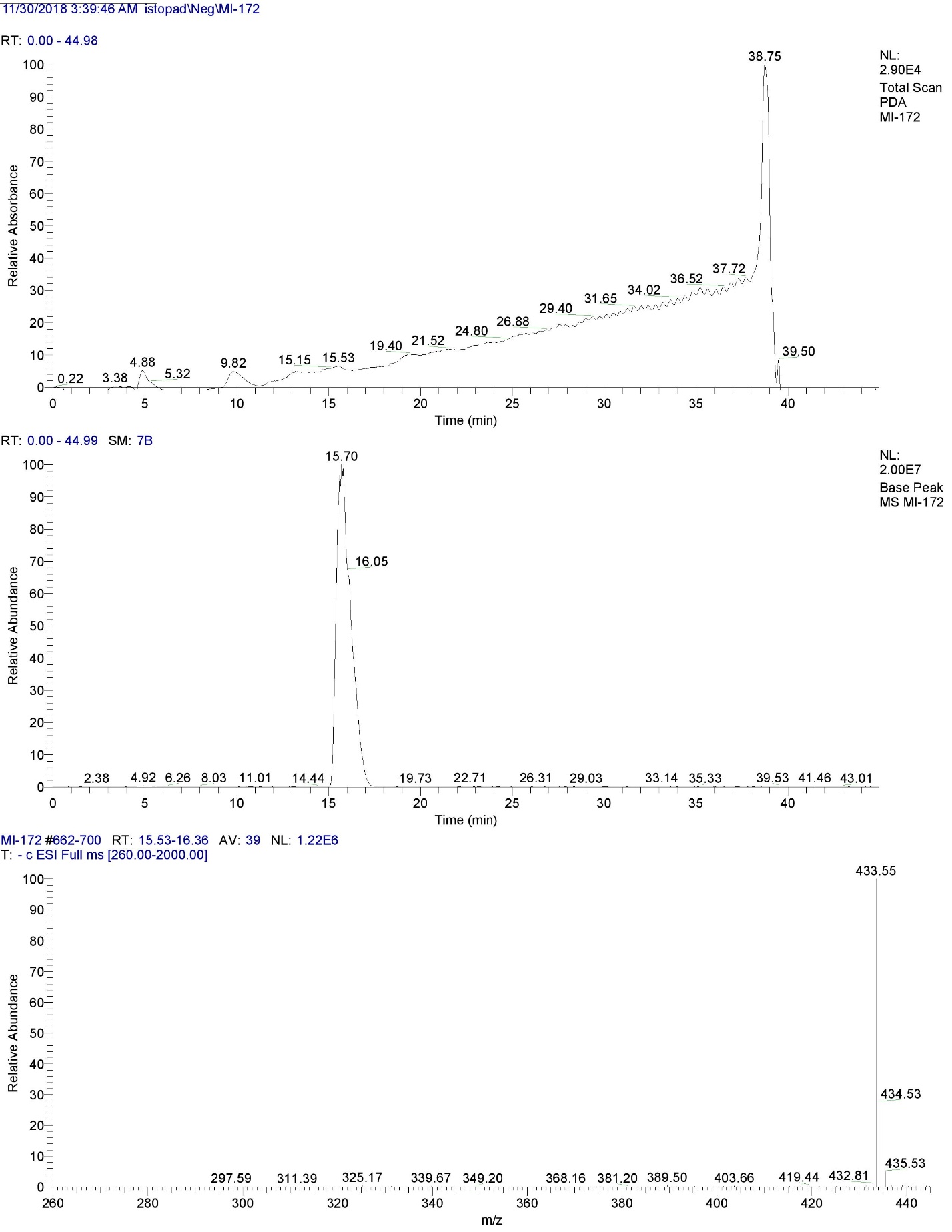


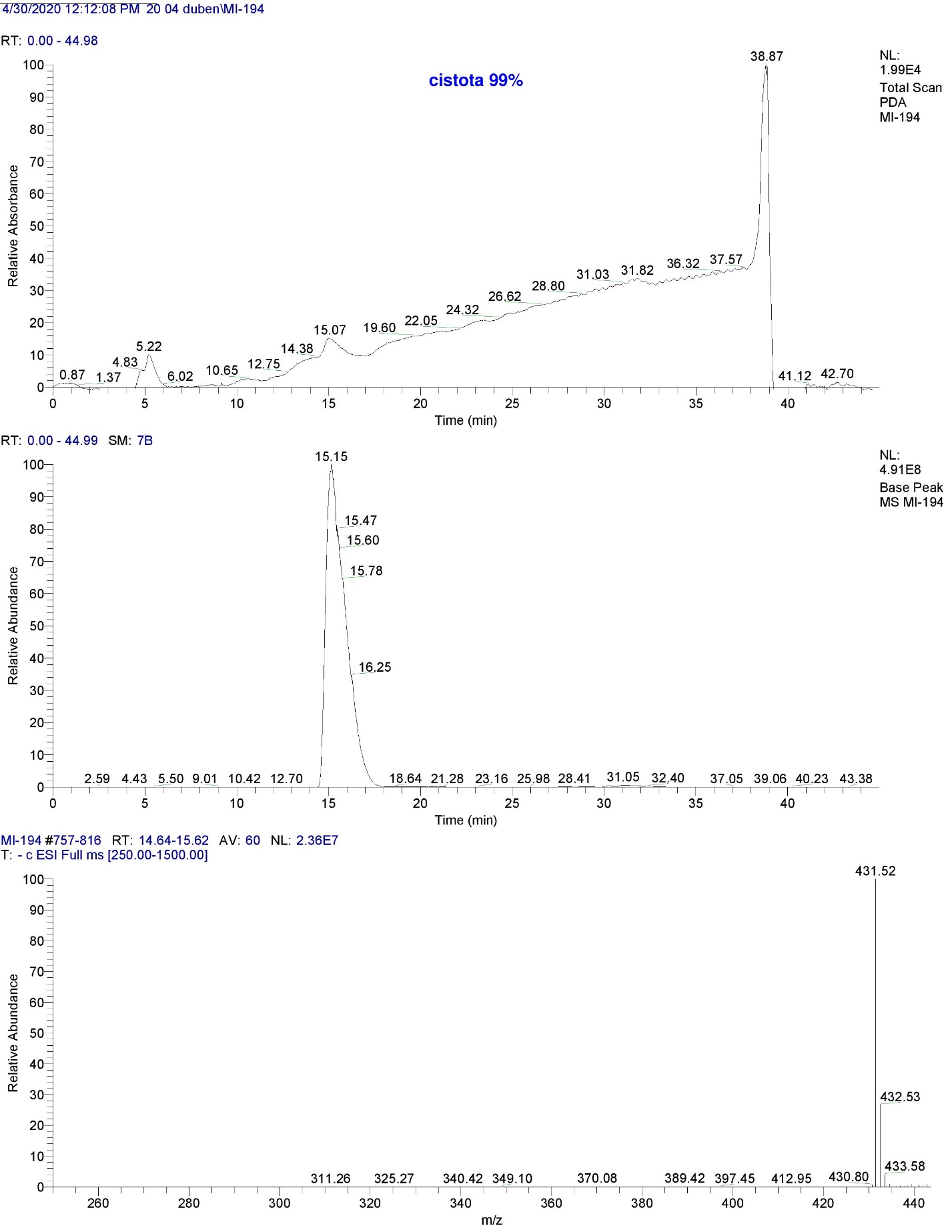


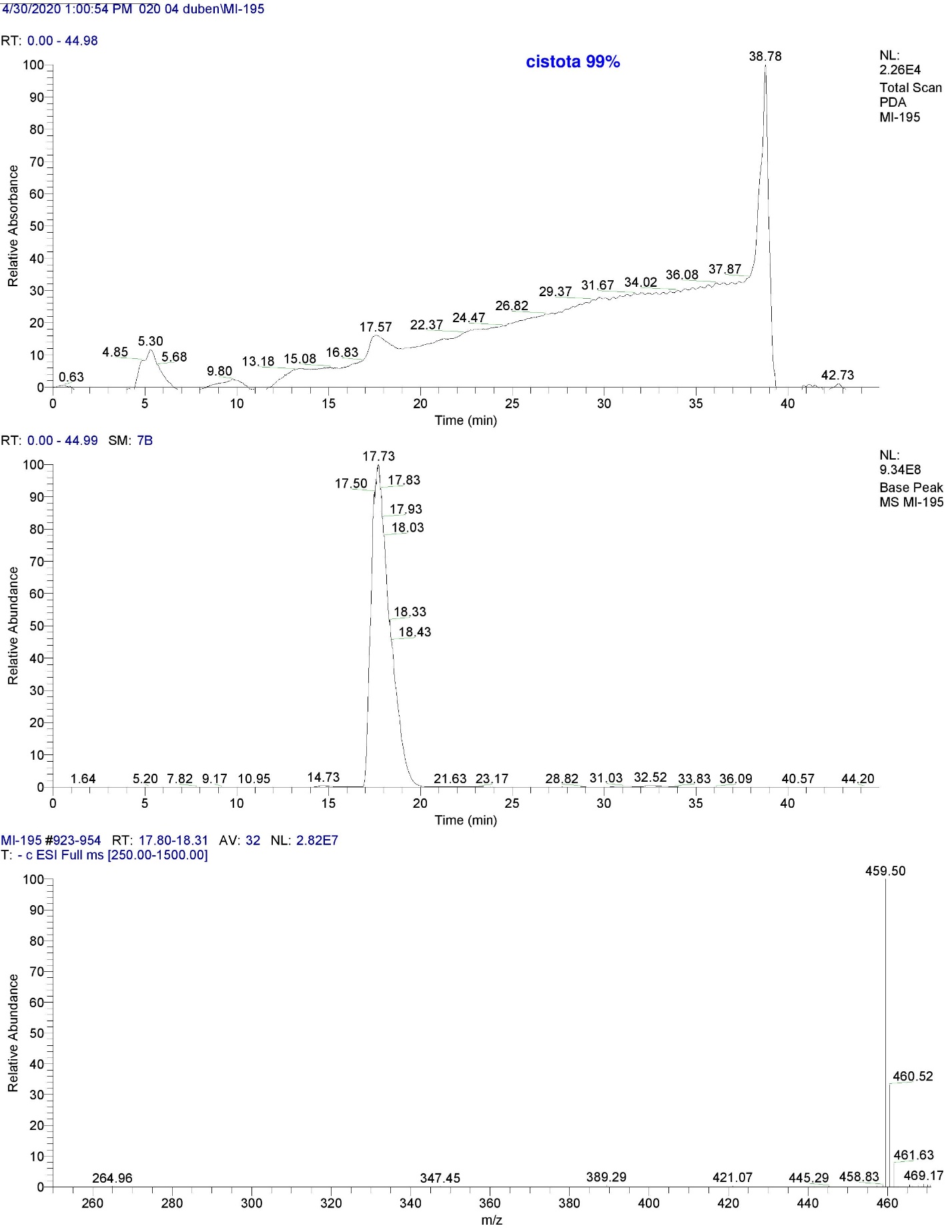


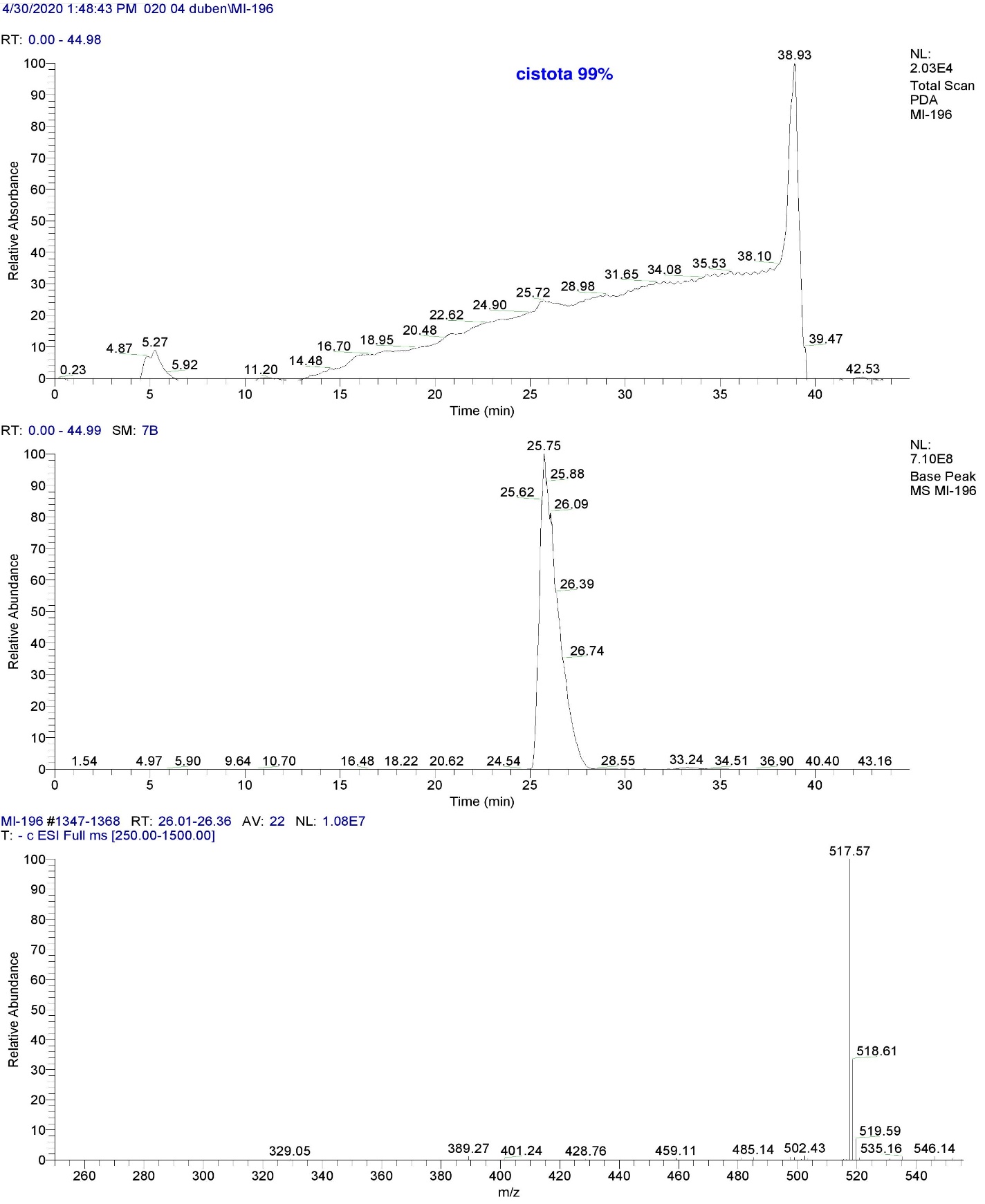


# LRMS


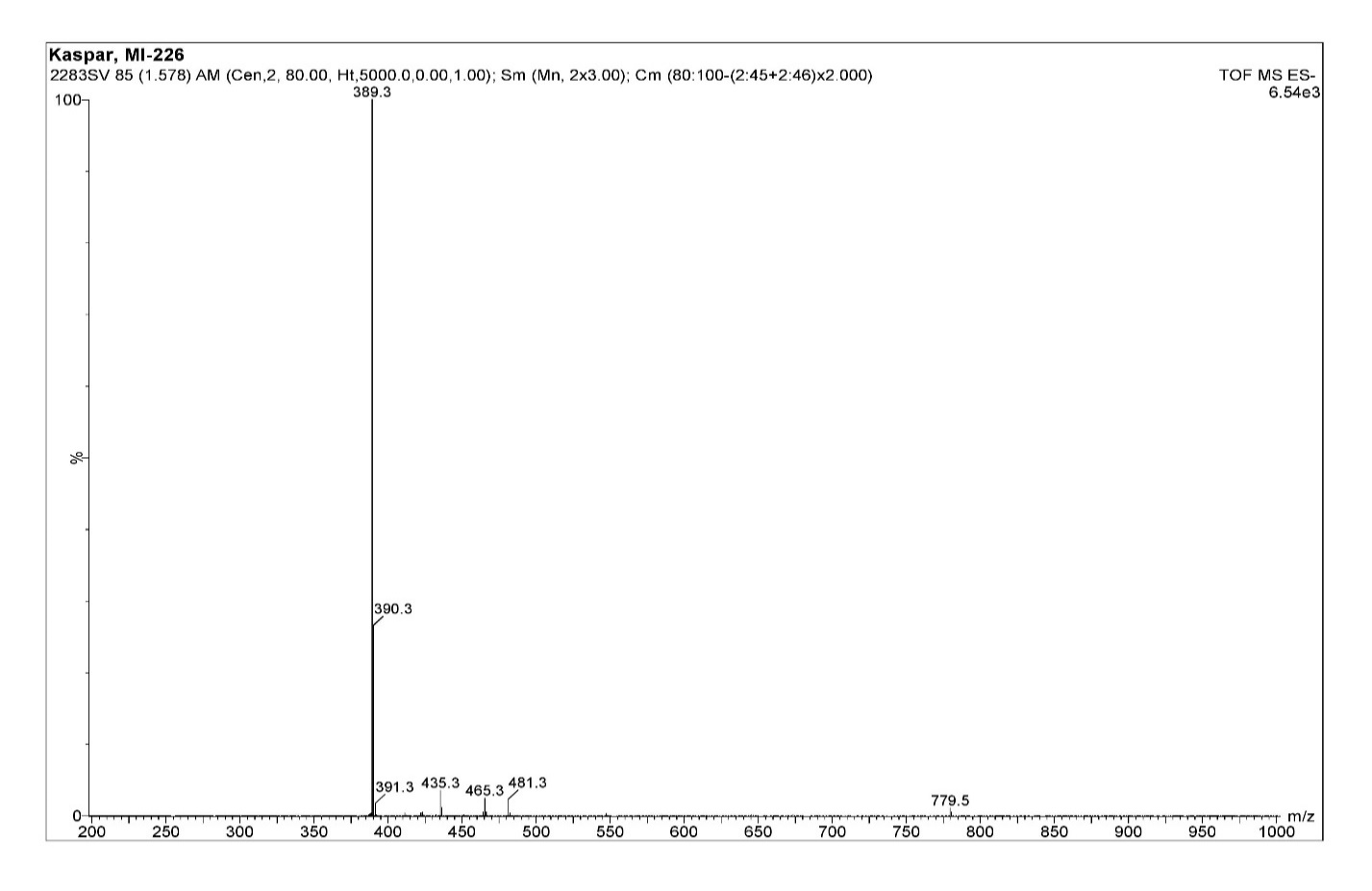


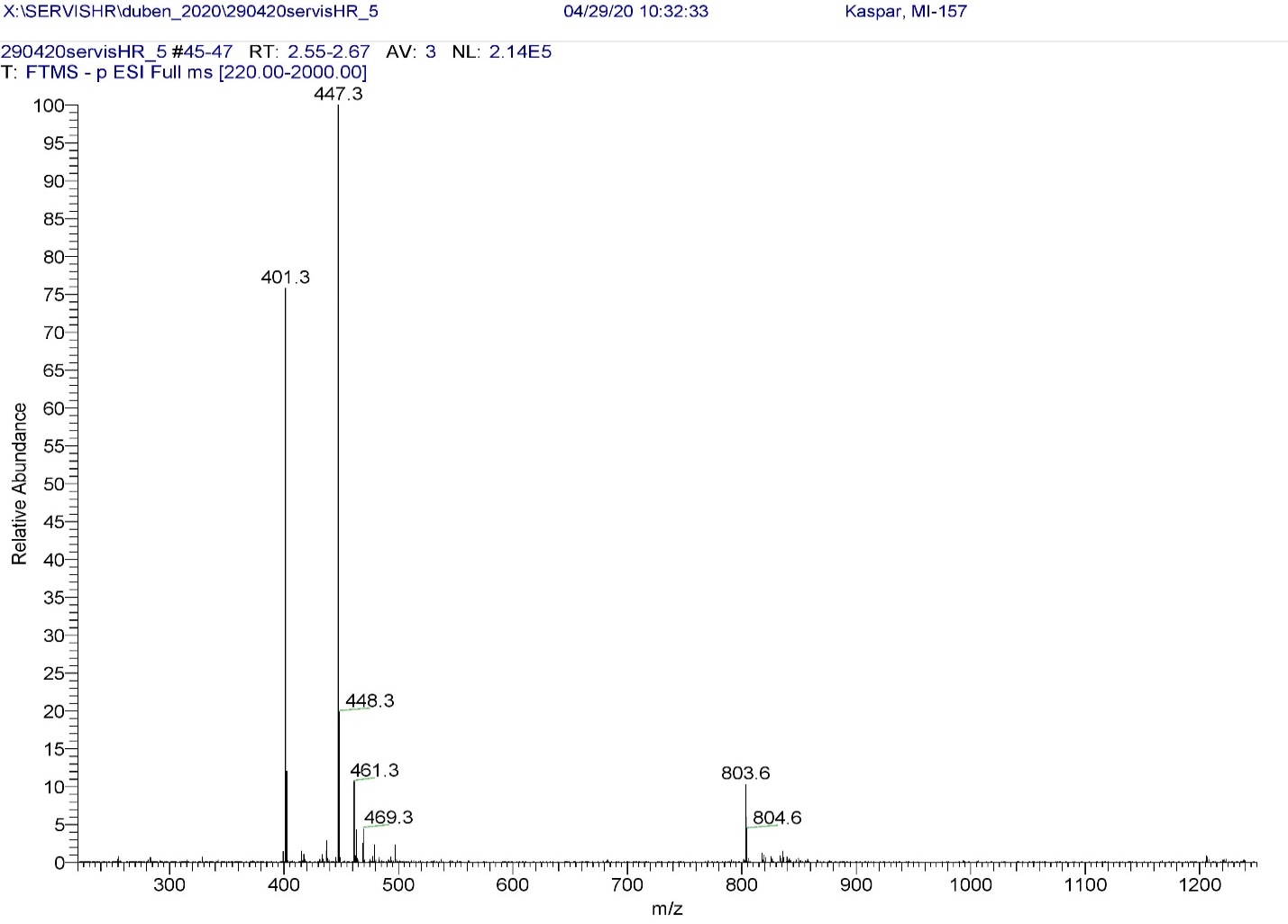


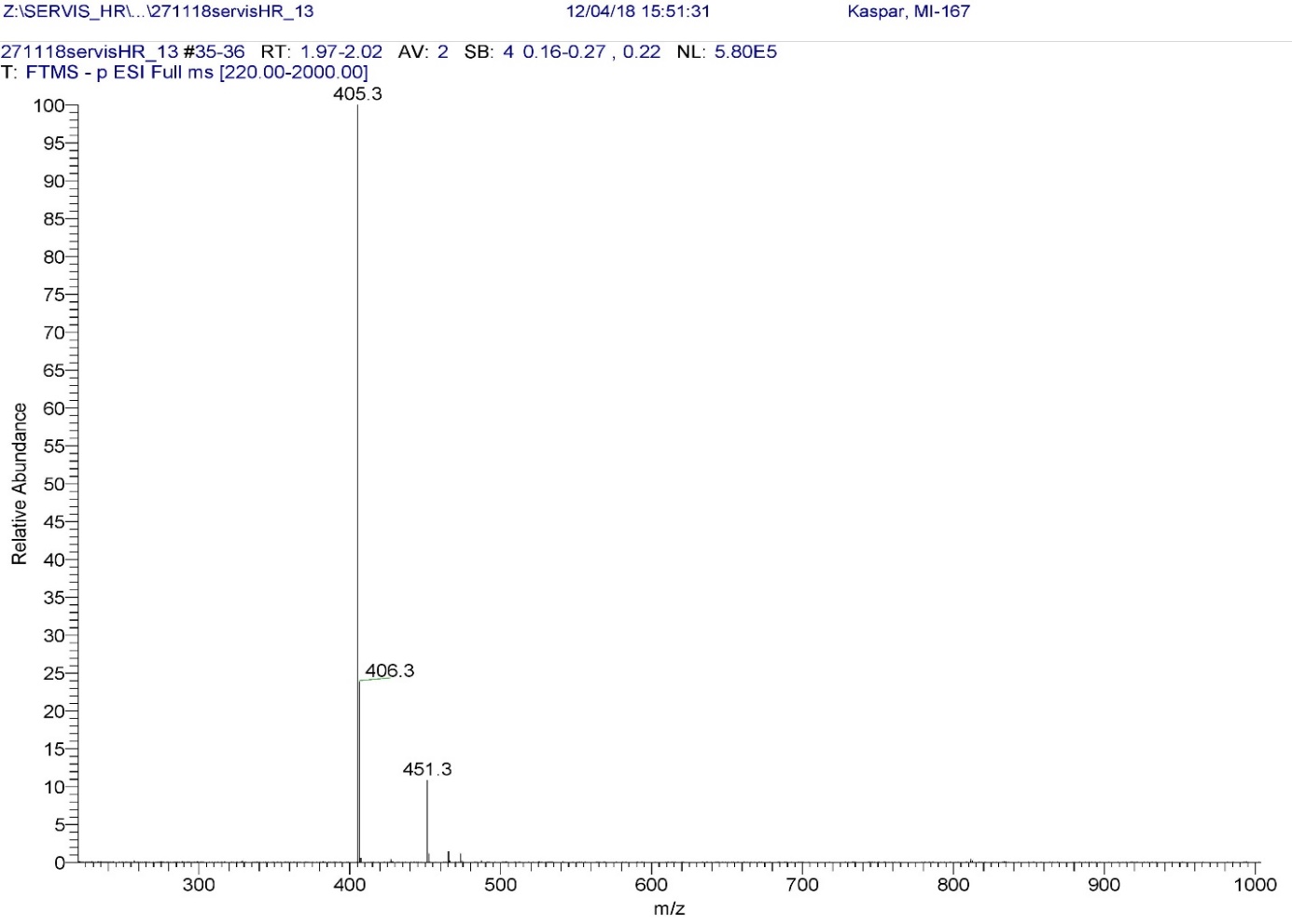


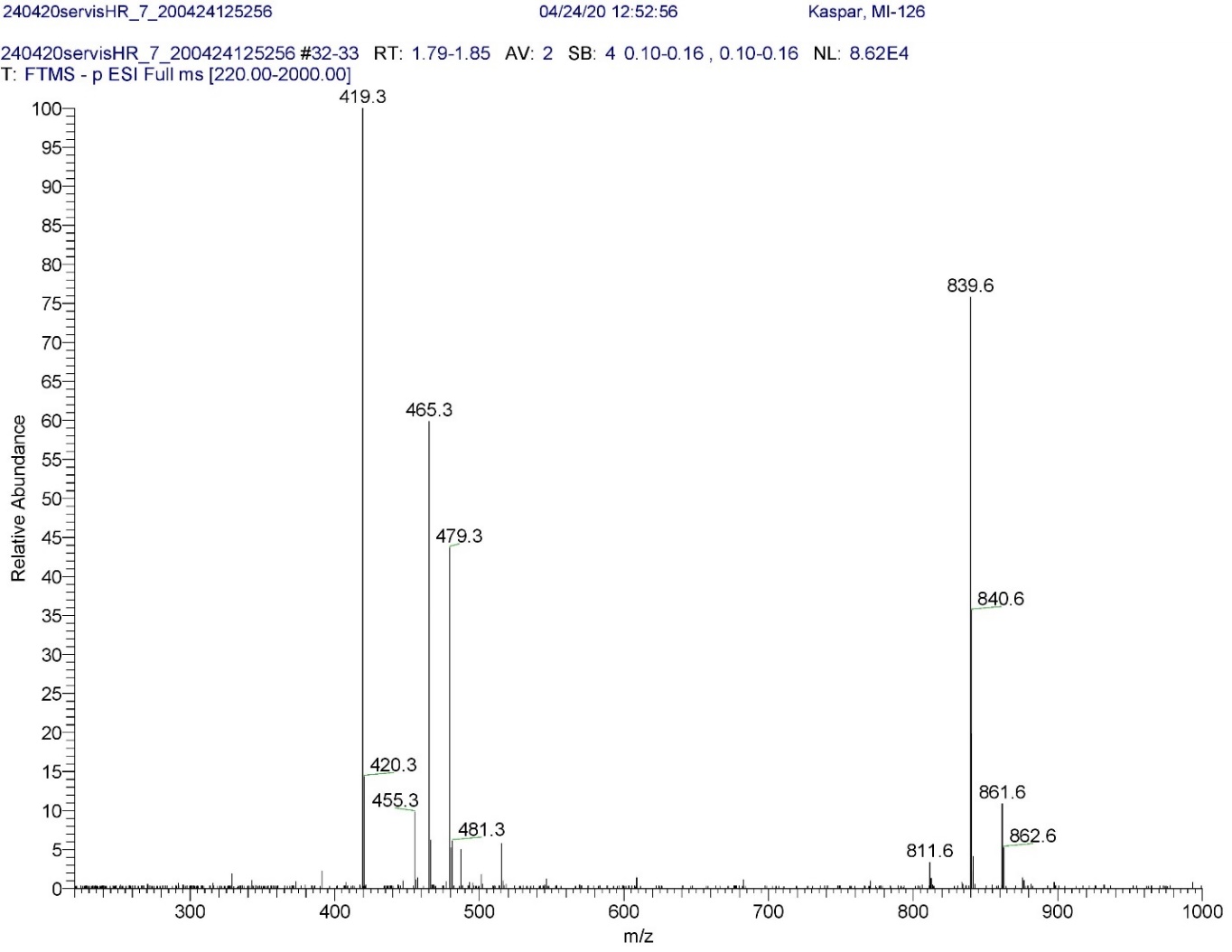


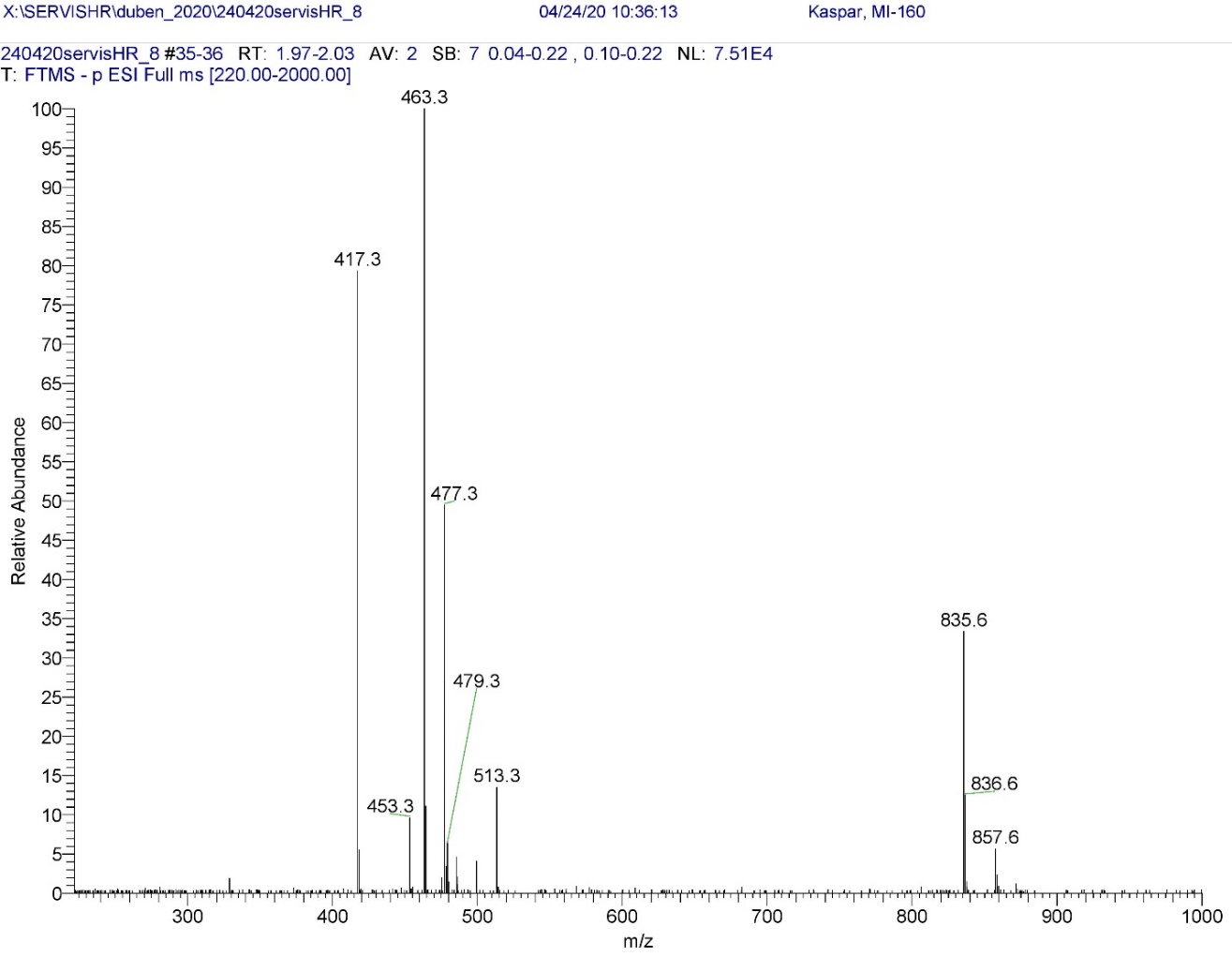


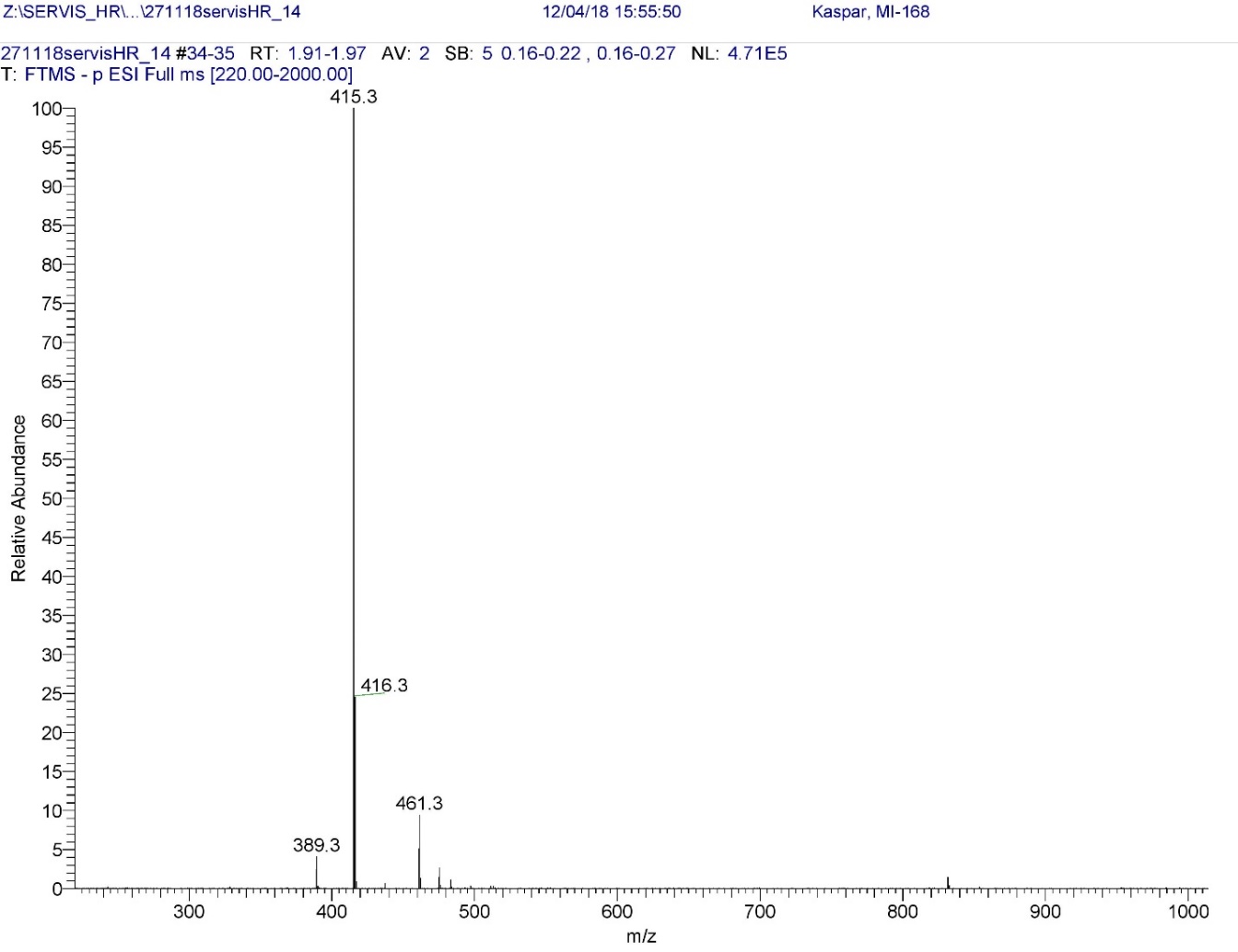


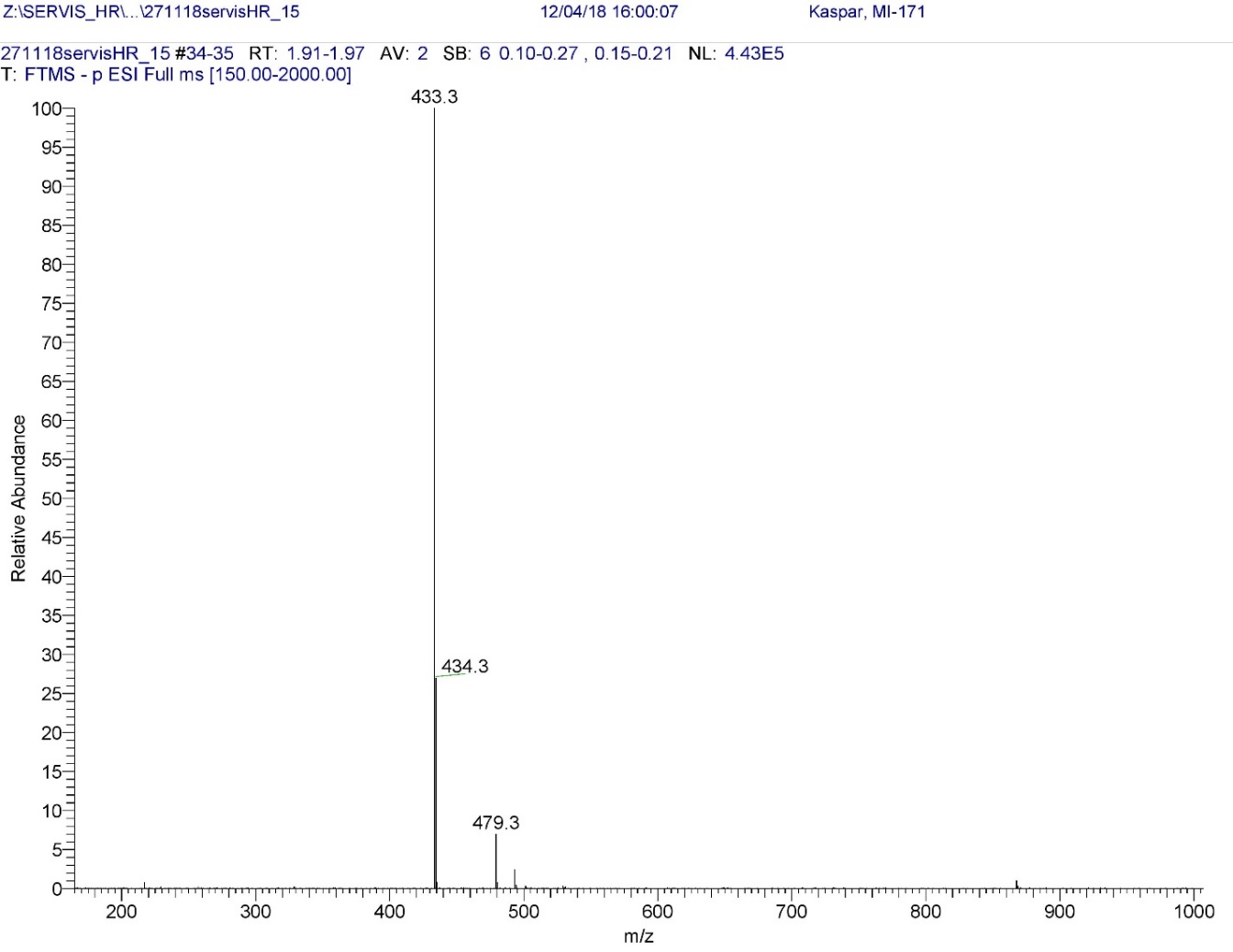


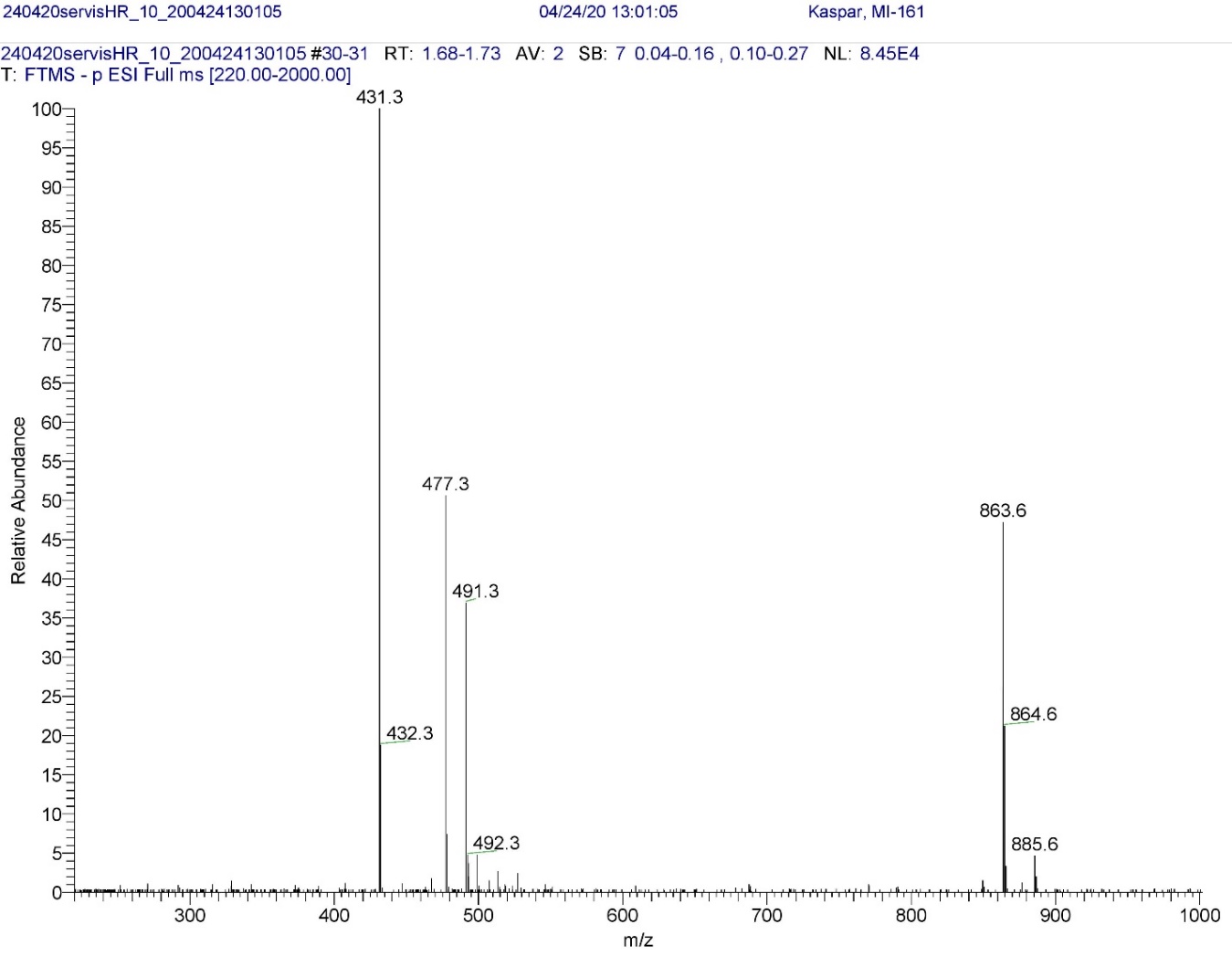


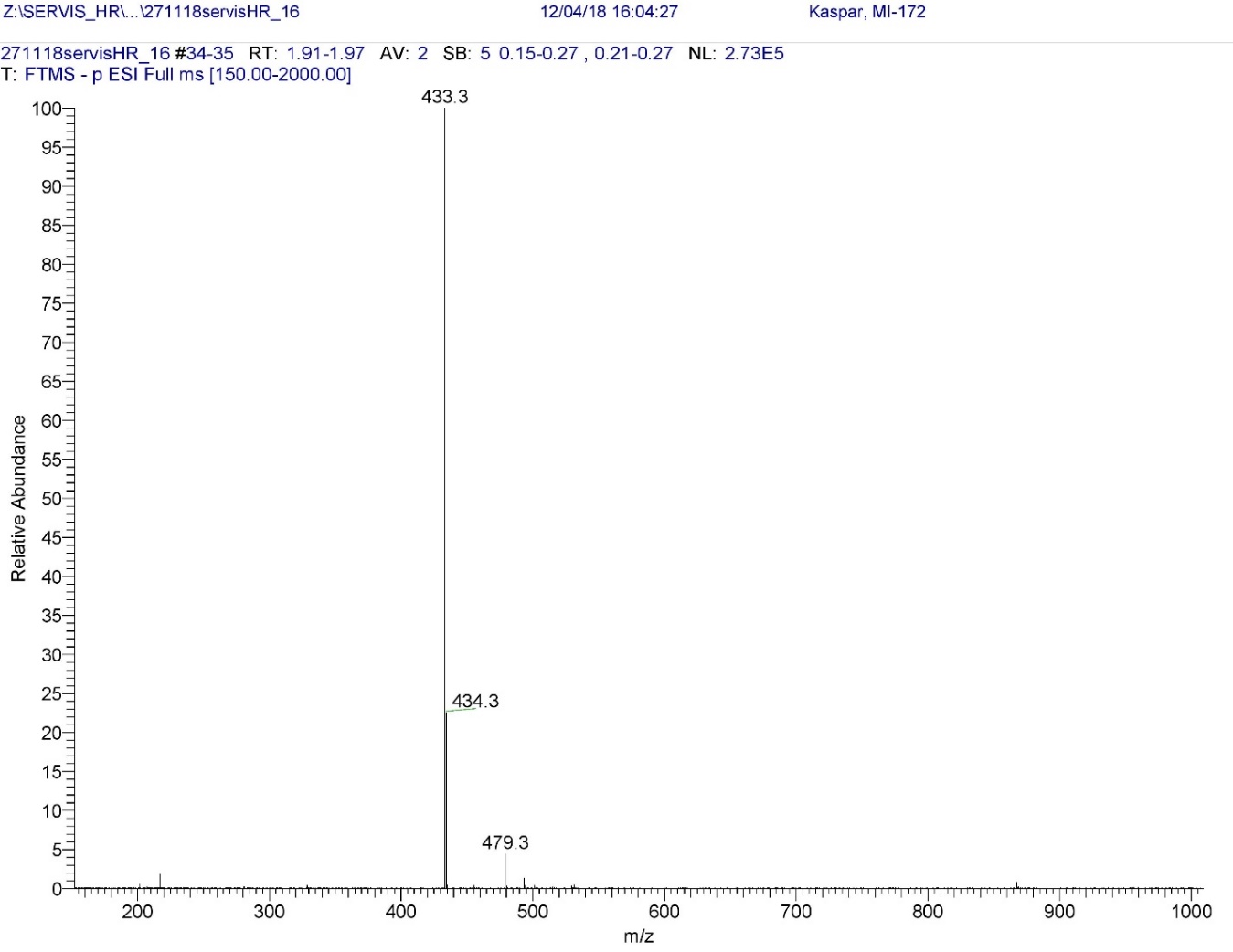


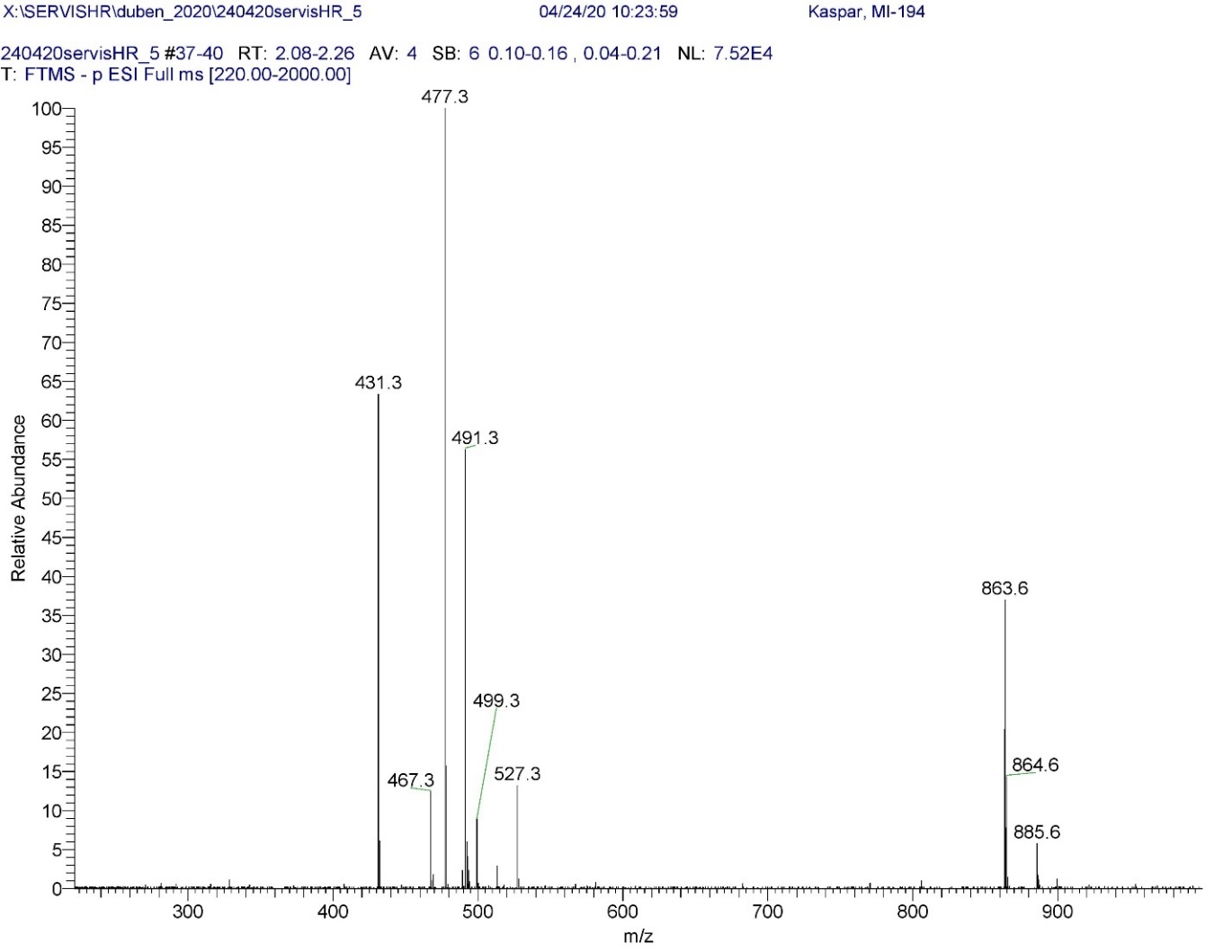


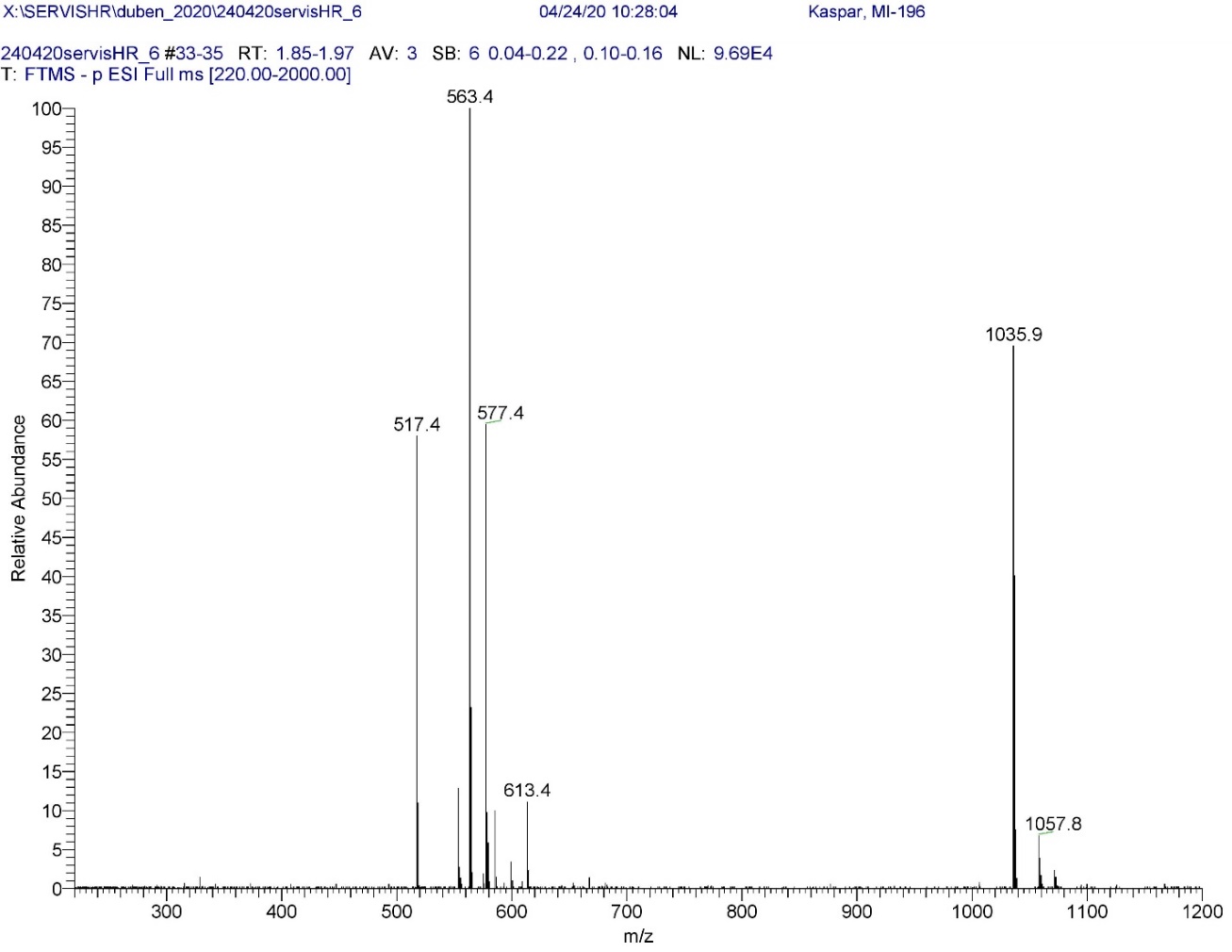

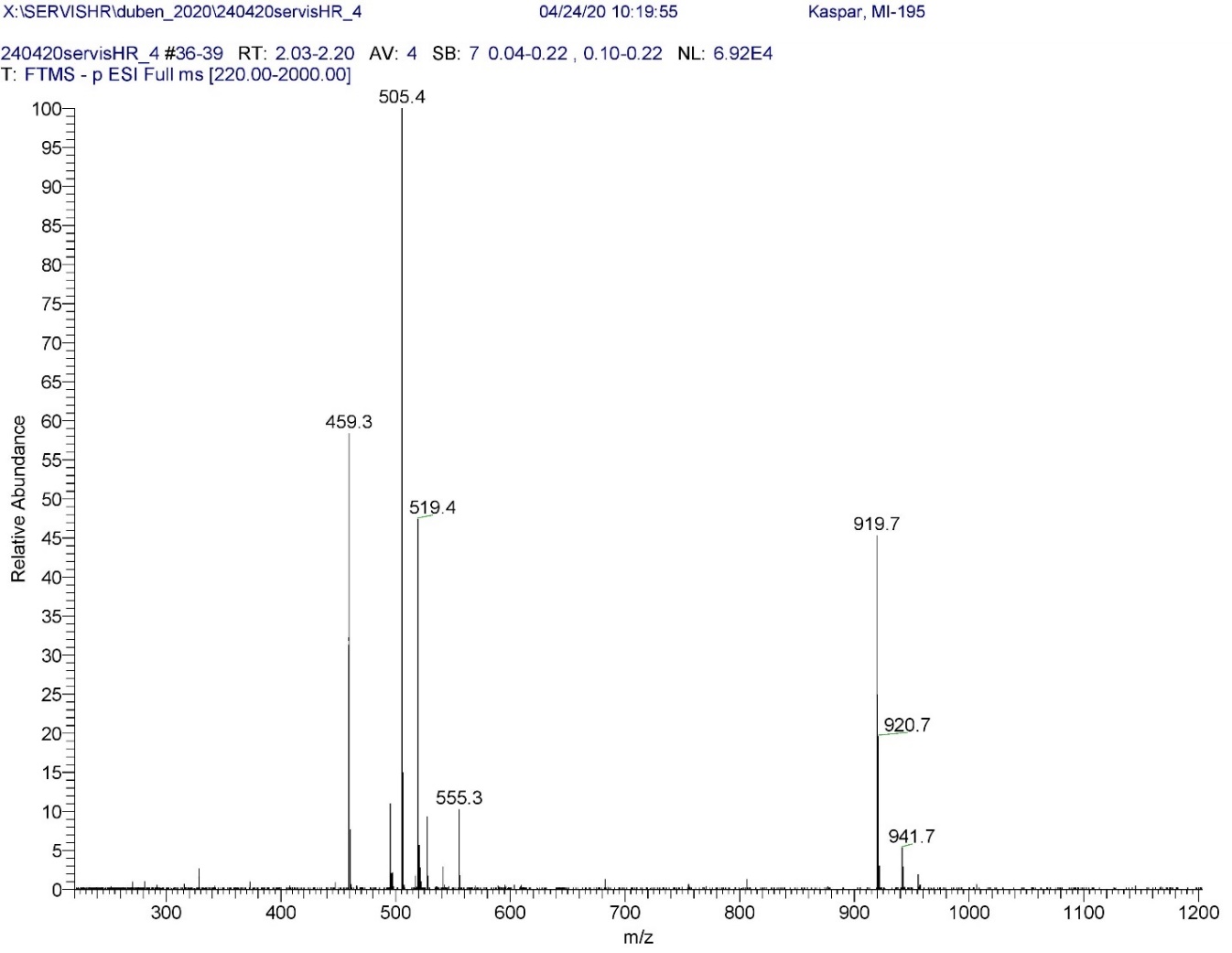

# ROESY spectrum of compounds 2a, 2d and 2g


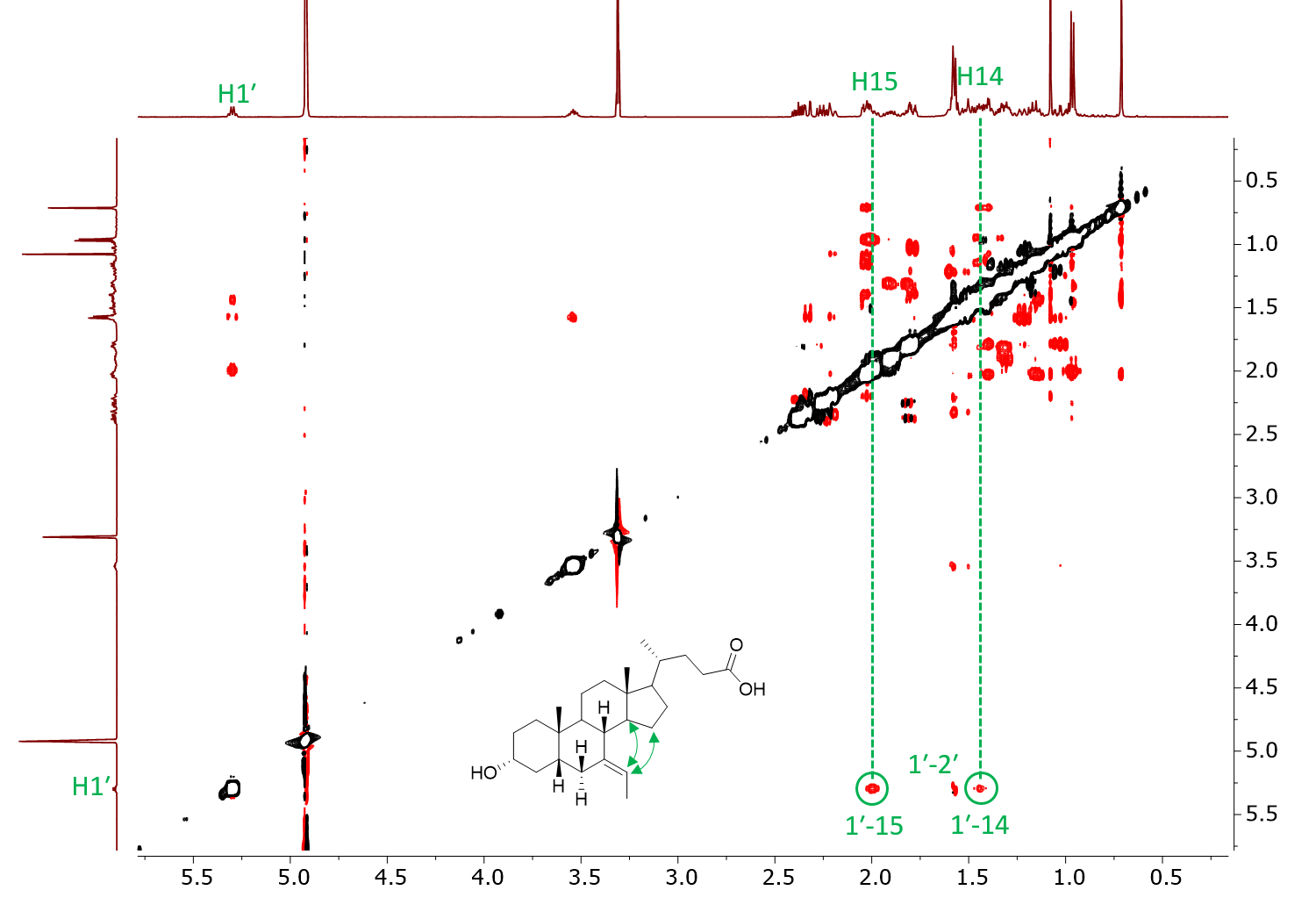


**Figure S9.** ROESY spectrum of **2a**


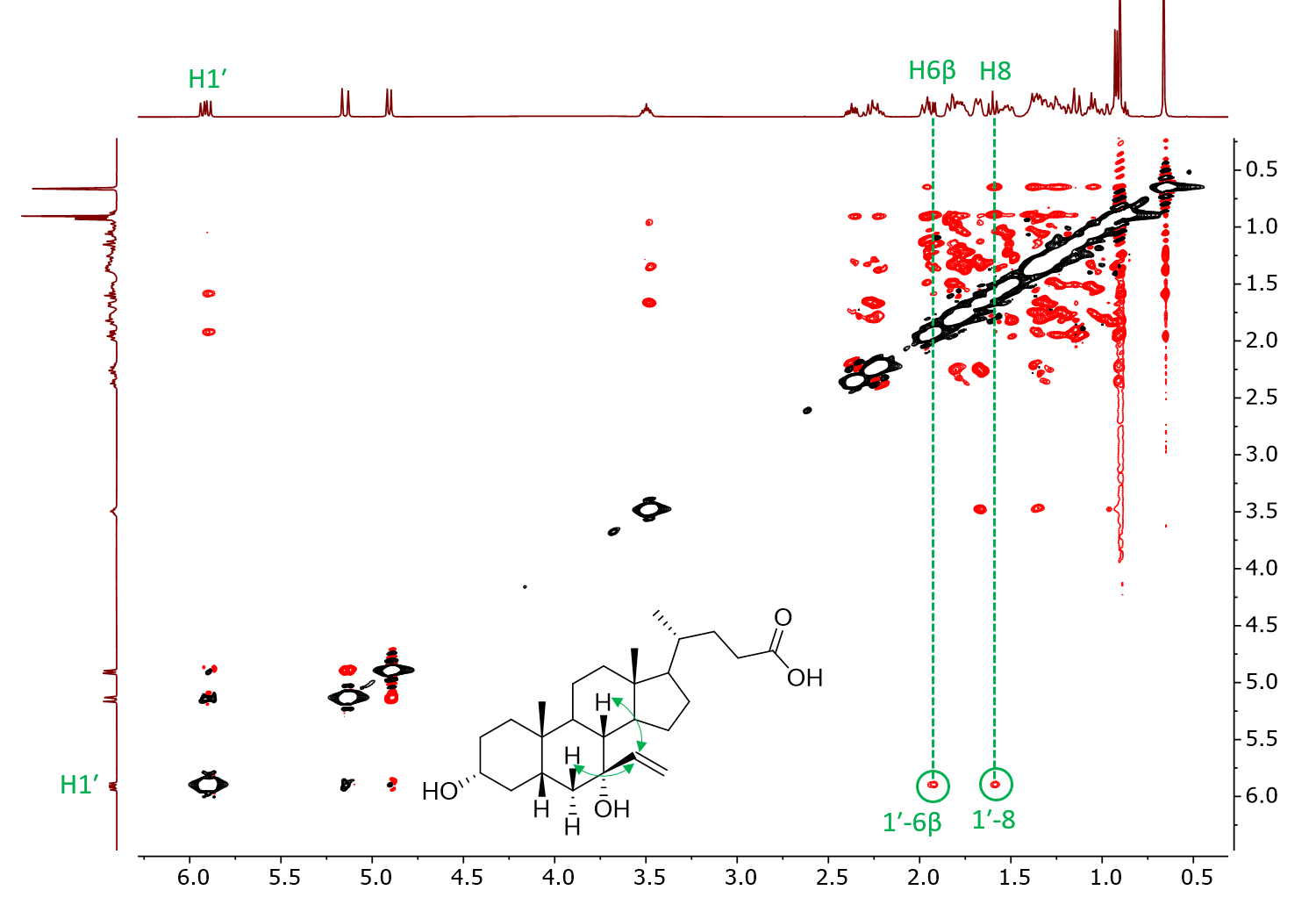


**Figure S10**. ROESY spectrum of **2d**


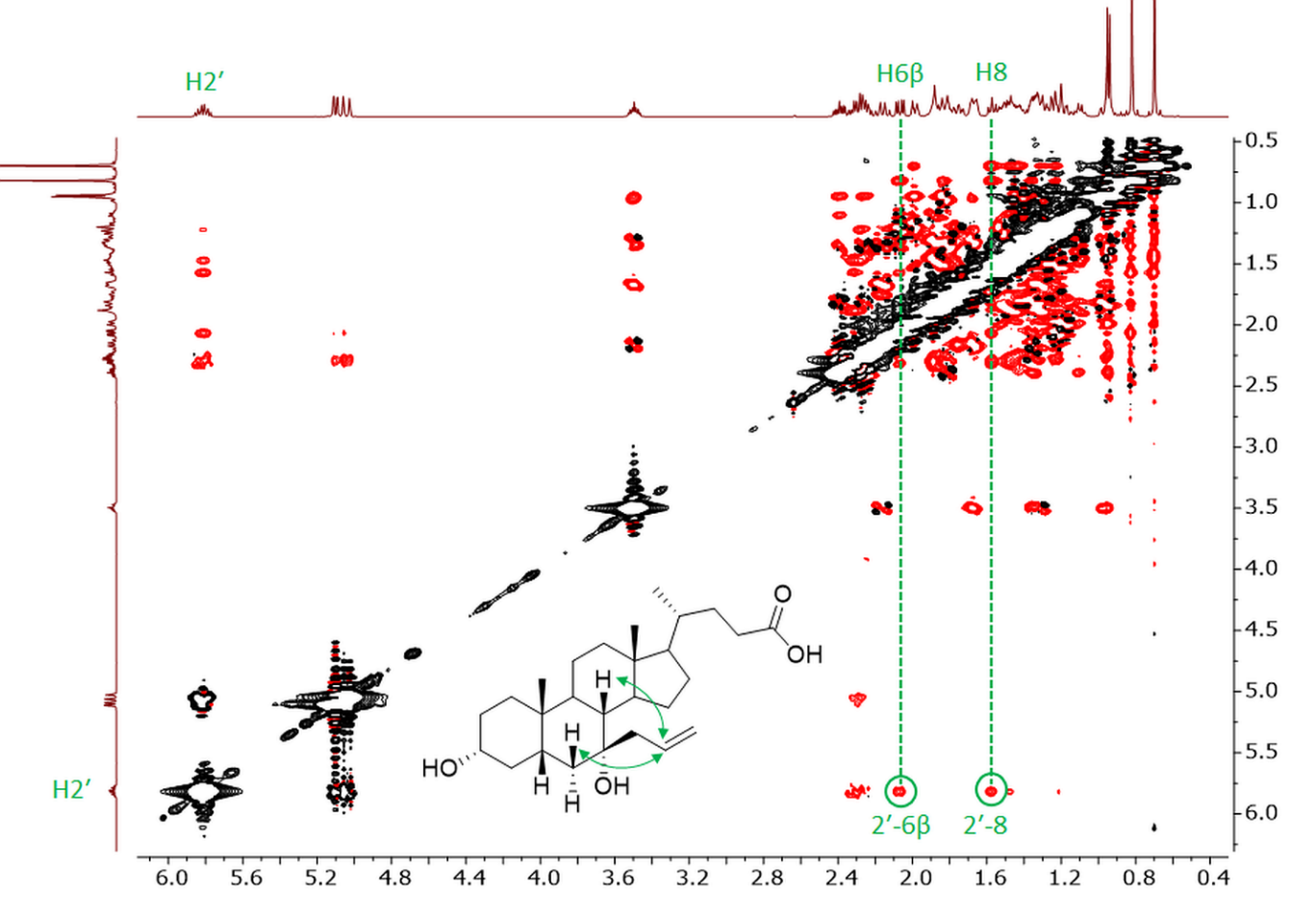


**Figure S11**. ROESY spectrum of **2g**

# The crystal data of compound 2h - data collection

The crystal data of compound **2h** were collected on an Xcalibur PX system, equipped with an Onyx CCD detector and a Cu Kα sealed tube (λ = 1.54178 Å) with an enhanced monochromator, using combined φ and ω scans at 180 K. CrysAlisProCCD(CrysAlisPro, 2002) was used for data collection, cell refinement and data reduction. The structure was solved by direct methods with SIR92(Altomare et al., 1994), and refined by full-matrix least-squares on F with CRYSTALS(Betteridge et al., 2003). The positional and anisotropic thermal parameters of all non-hydrogen atoms were refined. All hydrogen atoms were found from a Fourier difference map. Hydrogen atoms attached to carbon atoms were recalculated into idealized positions and refined with riding constraints. Those attached to oxygen atoms were refined isotropically. For detailed crystallographic data, see **Table S2**. The asymmetric unit contained two crystallographically independent molecules of **2h** and a partially occupied (75%) molecule of dichloromethane solvent, which was found to be disordered over two positions with equal occupancy.

ORTEP picture is in the full text.

**Table S2.** The crystallographic data and experimental parameters for compound **2h**.

| Parameter | Value |
| --- | --- |
| Formula | C_27_H_46_O_4_. 0.375(CH_2_Cl_)_ |
| Crystal size, mm^3^  Crystal system | 0.160 x 0.274 x 0.285  Orthorhombic |
| Space group | *P*2_1_2_1_2_1_ |
| Unit cell dimensions: |  |
| a, Å | 12.2954(4) |
| b, Å | 18.8572(6) |
| c, Å | 24.5090(7) |
| V, Å^3^ | 5682.6(3) |
| Z | 8 |
| F(000) | 2046 |
| T, K | 180 |
| μ, mm^-1^ | 1.181 |
| D_calc_, g/cm^3^ | 1.091 |
| 2Θ_max_, deg. | 133 |
| Measured reflections | 24766 |
| Independent reflections | 9978 |
| R_int_ | 0.025 |
| Obs. Reflections *I* > 2*σ*(*I*) | 8942 |
| Parameters | 623 |
| R_1_ | 0.0453 |
| wR_2_ | 0.0529 |
| S | 1.0914 |
| Flack Parameter | 0.09(3) |
| CCDC number | 2012020 |

# Acknowledgments

The authors wish to acknowledge the CSC – IT Center for Science, Finland, for the generous computational resources.

# References

Akwabi-Ameyaw A, Bass JY, Caldwell RD, Caravella JA, Chen L, Creech KL, Deaton DN, Jones SA, Kaldor I, Liu Y, Madauss KP, Marr HB, McFadyen RB, Miller AB, Navas F, III, Parks DJ, Spearing PK, Todd D, Williams SP and Wisely GB. (2008). Conformationally constrained farnesoid X receptor (FXR) agonists: Naphthoic acid-based analogs of GW 4064. Bioorg Med Chem Lett 18,4339-4343.

Altomare A, Cascarano G, G. G, A. G, C. BM, Polidori G and Camalli M. (1994). SIR92 - a program for automatic solution of crystal structures by direct methods. J Appl Cryst 27,435.

Betteridge PW, Carruthers JR, Cooper RI, Prout K and Watkin DJ. (2003). CRYSTALS version 12: software for guided crystal structure analysis. J Appl Cryst 36,1487.

CrysAlisPro. (2002). Oxford Diffraction.

Darden T, York D and Pedersen L. (1993). Particle Mesh Ewald: An N ⋅log( N ) Method for Ewald Sums in Large Systems. J Chem Phys 98,10089–10092.

Dror RO, Jensen MO, Borhani DW and Shaw DE. (2010). Exploring atomic resolution physiology on a femtosecond to millisecond timescale using molecular dynamics simulations. J Gen Physiol 135,555-562.

Feng S, Yang M, Zhang Z, Wang Z, Hong D, Richter H, Benson GM, Bleicher K, Grether U, Martin RE, Plancher JM, Kuhn B, Rudolph MG and Chen L. (2009). Identification of an N-oxide pyridine GW4064 analog as a potent FXR agonist. Bioorg Med Chem Lett 19,2595-2598.

Friesner RA, Banks JL, Murphy RB, Halgren TA, Klicic JJ, Mainz DT, Repasky MP, Knoll EH, Shelley M, Perry JK, Shaw DE, Francis P and Shenkin PS. (2004). Glide: a new approach for rapid, accurate docking and scoring. 1. Method and assessment of docking accuracy. J Med Chem 47,1739-1749.

Harder E, Damm W, Maple J, Wu C, Reboul M, Xiang JY, Wang L, Lupyan D, Dahlgren MK, Knight JL, Kaus JW, Cerutti DS, Krilov G, Jorgensen WL, Abel R and Friesner RA. (2016). OPLS3: A Force Field Providing Broad Coverage of Drug-like Small Molecules and Proteins. J Chem Theory Comput 12,281-296.

Jorgensen WL, Chandrasekhar J, Madura JD, Impey RW and Klein ML. (1983). Comparison of Simple Potential Functions for Simulating Liquid Water. J Chem Phys 79,926-935.

Kronenberger T, de Oliveira Fernades P, Drumond Franco I, Poso A and Goncalves Maltarollo V. (2019). Ligand- and Structure-Based Approaches of Escherichia coli FabI Inhibition by Triclosan Derivatives: From Chemical Similarity to Protein Dynamics Influence. ChemMedChem 14,1995-2004.

Merk D, Gabler M, Gomez RC, Flesch D, Hanke T, Kaiser A, Lamers C, Werz O, Schneider G and Schubert-Zsilavecz M. (2014). Anthranilic acid derivatives as novel ligands for farnesoid X receptor (FXR). Bioorg Med Chem 22,2447-2460.

Merk D, Sreeramulu S, Kudlinzki D, Saxena K, Linhard V, Gande SL, Hiller F, Lamers C, Nilsson E, Aagaard A, Wissler L, Dekker N, Bamberg K, Schubert-Zsilavecz M and Schwalbe H. (2019). Molecular tuning of farnesoid X receptor partial agonism. Nat Commun 10,2915.

Sastry GM, Adzhigirey M, Day T, Annabhimoju R and Sherman W. (2013). Protein and ligand preparation: parameters, protocols, and influence on virtual screening enrichments. J Comput Aided Mol Des 27,221-234.
